# Proteomic Signatures of Human Oral Epithelial Cells in HIV-Infected Subjects

**DOI:** 10.1371/journal.pone.0027816

**Published:** 2011-11-16

**Authors:** Elizabeth Yohannes, Santosh K. Ghosh, Bin Jiang, Thomas S. McCormick, Aaron Weinberg, Edward Hill, Faddy Faddoul, Mark R. Chance

**Affiliations:** 1 Center for Proteomics and Bioinformatics, Case Western Reserve University, Cleveland, Ohio, United States of America; 2 School of Dental Medicine, Case Western Reserve University, Cleveland, Ohio, United States of America; 3 Department of Dermatology, Case Western Reserve University, Cleveland, Ohio, United States of America; 4 Department of Genetics, Case Western Reserve University Cleveland, Ohio, United States of America; Rush University, United States of America

## Abstract

The oral epithelium, the most abundant structural tissue lining the oral mucosa, is an important line of defense against infectious microorganisms. HIV infected subjects on highly active antiretroviral therapy (HAART) are susceptible to comorbid viral, bacterial and fungal infections in the oral cavity. To provide an assessment of the molecular alterations of oral epithelia potentially associated with susceptibility to comorbid infections in such subjects, we performed various proteomic studies on over twenty HIV infected and healthy subjects. In a discovery phase two Dimensional Difference Gel Electrophoresis (2-D DIGE) analyses of human oral gingival epithelial cell (HOEC) lysates were carried out; this identified 61 differentially expressed proteins between HIV-infected on HAART subjects and healthy controls. Down regulated proteins in HIV-infected subjects include proteins associated with maintenance of protein folding and pro- and anti-inflammatory responses (e.g., heat-shock proteins, Cryab, Calr, IL-1RA, and Galectin-3-binding protein) as well as proteins involved in redox homeostasis and detoxification (e.g., Gstp1, Prdx1, and Ero1). Up regulated proteins include: protein disulfide isomerases, proteins whose expression is negatively regulated by Hsp90 (e.g., Ndrg1), and proteins that maintain cellular integrity (e.g., Vimentin). In a verification phase, proteins identified in the protein profiling experiments and those inferred from Ingenuity Pathway Analysis were analyzed using Western blotting analysis on separate HOEC lysate samples, confirming many of the discovery findings. Additionally in HIV-infected patient samples Heat Shock Factor 1 is down regulated, which explains the reduced heat shock responses, while activation of the MAPK signal transduction cascade is observed. Overall, HAART therapy provides an incomplete immune recovery of the oral epithelial cells of the oral cavity for HIV-infected subjects, and the toxic side effects of HAART and/or HIV chronicity silence expression of multiple proteins that in healthy subjects function to provide robust innate immune responses and combat cellular stress.

## Introduction

Oral lesions occur in a high percentage of HIV infected subjects on highly active antiretroviral therapy (HAART). These lesions are initiated at oral mucosal surfaces and are due to opportunistic co-viral, bacterial, and fungal infections that promote, periodontitis, candidiasis, salivary gland disease, apthous ulcers, and oral warts [1], [2]. Although the severity of several HIV-related oral complications has decreased, while life expectancy has increased with the advent of HAART, certain oral complications remain [3], [4]. In fact, the incidence of some oral complications including oral warts, and salivary gland diseases among HIV infected subjects appears to have increased following the introduction of HAART, while others such as candidiasis, periodontitis and caries persist [5], [6], [7], [8]. The fact that some oral sequelae are on the rise, while others persist in optimally treated HIV infected individuals poses major problems in patient management.

HAART can profoundly suppress HIV replication and reconstitutes CD4+ cell counts thus partially preventing HIV-associated oral opportunistic infections such as candidiasis [9], [10]. However, the increases in the occurrence of co-viral initiated infections such as human papilloma virus (HPV) and its associated oral warts, epstein-barr virus (EBV) initiated oral hairy leukoplakia (OHL), and salivary gland diseases with persistent use of HAART [6], [7], [11] is troubling and unexpected. There is no evidence to suggest that these viruses behave differently in HIV-infected compared to non HIV-infected individuals. However, the lesions caused by these viral co-infections persist and progress into malignant phenotypes among HIV-infected individuals [5], [12]. In fact a recent meta-analysis reported an increased risk for infection related cancers; i.e., HPV, in two immuno-compromised cohorts; HIV-infected on HAART subjects and renal transplant subjects [13]. It is therefore possible that the restoration of the immune system by HAART may be functionally incomplete and, therefore, its effectiveness in repelling different pathogenic micro-organisms may vary [14], [15], [16] or, alternatively, that toxicity associated with HAART may contribute to oncogenesis. However, this has not been explored completely [17], [18], particularly in the setting of HAART use over prolonged periods of time. Furthermore, the limited beneficial effects of HAART towards secondary lesions might also be due to damage at the molecular level that may have occurred in the host epithelium during HIV infection and/or infections prior to initiating HAART, that possibly allows the comorbid infection to initiate and persist despite CD4-cell count improvement following initiation of therapy.

HIV and/or HAART mediated changes at the molecular level in oral epithelium of HIV infected on HAART subjects are poorly understood and limited to the genome level [5]. Although mRNA expression analyses have revealed significant changes in host gene expression, it remains unclear what consequences these changes bring about at the protein level. Therefore, we pursued the opportunity to advance our molecular understanding of oral lesions in HIV-infected on HAART subjects by directly measuring proteome expression changes in human oral epithelial cells (HOECs). The present study was undertaken to elucidate the host proteome response in HOECs from HIV-infected on -HAART vs. control individuals, thereby providing a detailed understanding of likely functional changes in cellular processes that may contribute to comorbid infections. Differential protein analysis was carried out in a discovery phase using Two Dimensional Difference Gel Electrophoresis (2-D DIGE) coupled with mass spectrometry identification, and a subsequent verification analysis (using Western blotting) was carried out to identify, compare and confirm proteins that are changing in HIV-infected on -HAART subjects vs. HIV-negative individuals.

To leverage the protein abundance changes, identified targets were used as inputs for bioinformatics analysis of pathways likely involved in the disease process [19], [20]. Thus, we analyzed the targets identified by the 2-D DIGE experiment using Ingenuity Pathway Analysis (IPA) to infer potential master regulators of the processes of interest [21]. IPA analyses provided additional molecular targets relevant to disease, and some of these hypothesized targets were also confirmed by Western blotting. With this integrated approach we have identified a number of differentially expressed proteins that provide valuable mechanistic insights into the molecular changes that are precursors for comorbid oral pathogen infections in HIV-infected on HAART subjects.

## Results

### CD4+ cell count and viral load in HIV-infected subjects

A total of 11 HIV-infected male subjects averaging 46.4±8.7 years of age and 10 healthy donors averaging 22.9±6.5 years of age and including both sexes were included in this study. Although there is a systematic age difference between HIV-infected and health controls, all subjects are adults ranging from 18 to 58 years old. Data on the CD4 cell counts and viral load for the HIV-infected on HAART subjects are shown in Table S1. All HIV-infected subjects are on HAART except subjects 8 (fully HAART naïve) and 11 (3 months off HAART at time of tissue collection). CD4 cell counts at time of tissue collection compared to nadir CD4 indicate that there was a overall 4-fold recovery of CD4 after HAART treatment for HAART treated subjects. Nadir CD4 averaged ∼160 indicating that all patients suffered significant drops in immune function at some point during the course of their disease. Also, nadir CD4 was very similar for the discovery (mean ∼150 cells/mL) and validation groups (mean ∼168), showing consistency between the discovery and validation cohorts. Viral loads averaged less than 20,000 copies/mL blood for the four subjects used for discovery proteomics studies. Viral loads averaged under 3000 copies/ml of blood for the HIV-infected patient samples used for verification analysis except for the two HAART naïve patients who had much higher level of virus, as expected.

### Proteome expression profile and statistical analysis

The initial discovery experiment was designed and performed to monitor statistically significant changes in proteome expression in HIV-infected HOECs while minimizing individual patient based systematic variations by pooling samples from the two separate patient groups. Although this reduced inter-patient variation it eliminated the ability to examine patient specific responses. The average CD4+ cell count per mL of the HIV-infected group for discovery (subjects 1–4) was 517 while that for validation (subjects 5–10) was 770 (not significantly different), compared to nadir CD4 which averaged less than 170, indicating a significant response to HAART treatment. Immunological recovery is considered by many investigators to be above 700 cells/mL and accumulating evidence suggests levels of CD4+ recovery are dependent on whether treatment is started before CD4+ counts fall too low [22]. The two lowest CD4+ counts are for the two HAART naïve patients, as expected.

The oral (gingival) tissue extracted from subjects, typically during dental procedures such as molar extractions, is heterogeneous, and expansion of cells (without passage) was performed to promote overgrowth of epithelia. This provided a primary and homogenous cell population for proteomics analysis. Expansion also increased the material available for proteomics analysis, as the gingival tissue from subjects is limited. In the absence of the expansion step, proteomic results could not be attributed to a specific tissue type. Thus, HOEC samples consisted of expanded cells from 4 HIV-infected subjects and from 4 healthy controls where the cells from each group were pooled after expansion. Then, the cells from each group were divided into replicates of 4 and an internal standard composed of an equal amount of all samples was generated as well, such that 4 gels were run with 3 samples (including an internal standard) included in each (Table 1).

**Table 1:** Experimental design for 2-D DIGE proteome profiling.

Gel	Cy3	Cy5	Cy2
1	N-1	HIV-1	Pooled sample (N-1 to N4 + HIV-1 to HIV-4)
2	HIV-2	N-2	Pooled sample (N-1 to N4 + HIV-1 to HIV-4)
3	N-3	HIV-3	Pooled sample (N-1 to N4 + HIV-1 to HIV-4)
4	N-4	HIV-4	Pooled sample (N-1 to N4 + HIV-1 to HIV-4)

Biological replicate samples for each group were pooled (HIV1-4; HIV-infected on HAART, N1-4; healthy controls) and the pool was split into four 50 µg of aliquots of technical replicates that were then labeled with Cy3 or Cy5. Each gel contained the pooled standard (Pooled Std; mix of equal aliquots of each sample in all experimental groups) and two other subject samples. Thus, the 8 samples were analyzed in triplicate by running four gels. (For detail descriptions refer “Materials and Methods section.”).

Sample size for this study was estimated from power analysis that was performed using previous 2D-DIGE datasets [20], and (Yohannes et al, unpublished data). Power curves, (Figure S1), were generated per hypothesis of the two-sided t-test as a function of sample size to detect 50% change in protein expression with Type I error probability α = 0.05 or 0.001 using datasets from technical and biological replicates. It is clear from these curves that variance (technical and/or biological), significance level, and sample size have significant effect on power. For pooled samples, the within variance among replicates arises from technical noise only. Thus as sample numbers are limiting, the power curve from the technical replicate at α = 0.05 is considered and from this curve it is clear that using three technical replicates results in >80% chance of detecting a 50% change. Thus, we designed a study with pooled samples and four technical replicates (over 90% power). Accordingly spots with 1.5 and above or −1.5 and below fold changes and exhibiting statistical significance (T-test P-value<0.05), were retained as spots of interest in this study. Out of the total number of spots detected, 1413 were matched in all 12-gel images (essentially 80% of the total) and the spots deemed significant were carefully checked for correct matching in every gel. One hundred fifty three spots, which passed all these criteria, were considered to be significantly different between the two experimental groups, and were further investigated.

The subsequent FT-MS/MS analysis on a total of 153 spots of interest successfully derived sequence data from 131 spots, whereas the digest for the rest of the 22 spots either did not reveal any peptides or identified keratin. A total of 20 spots contained isoforms for 8 proteins due to either post-translational modification or proteolysis. As the overall expression patterns of the observed isoforms were similar, duplicates were omitted from the list in Table S2. Moreover, for 58 spots, more than one protein per spot passed our stringent filtering criteria and the results for these spots were also excluded from the list (Table S2) as the average ratio observed corresponds to the combination of all the proteins present for a particular spot and individual changes are not conclusive. Thus we report a total of 61 unique proteins along with the fold changes that are summarized in Table S2 and detailed identification information is provided in the Table S3.

**Figure 1:**
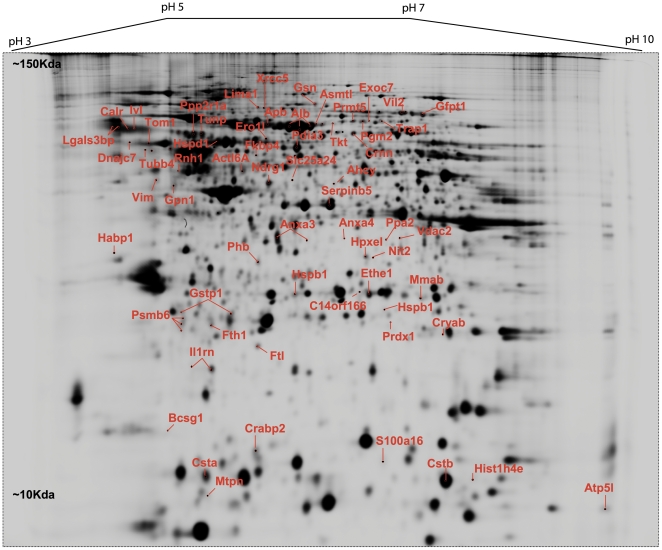
The 2D map of deep purple-labeled oral epithelial cellular proteome indicating pick location of a subset of proteins that changed in response to HIV and/or HAART. Orientation of the pH gradients is indicated on the *horizontal axes* from 3 pH units (*left*) to 10 pH unit (*right*), and approximate apparent molecular mass ranges are indicated along the vertical axes 10 kDa (*bottom*) to 200 kDa (*top*).

A 2D-map of a representative deep purple stained gel is shown in Figure 1. On this map the pick location of proteins whose expression is significantly different based on the variance of the mean change between the groups (P≤0.05) are shown. In some cases, the same protein was identified in different spots across the 2D gel, suggesting the occurrence of post-translational modifications. The protein abundance changes we report as significant (Table S2) are for the proteins that have ≥1.5 or ≤−1.5 changes in expression in the HIV-infected compared to HIV-negative control subjects. A significant portion of these proteins are down regulated in HIV-infected subjects compared to the healthy controls.

#### Principal component analysis

**Figure 2:**
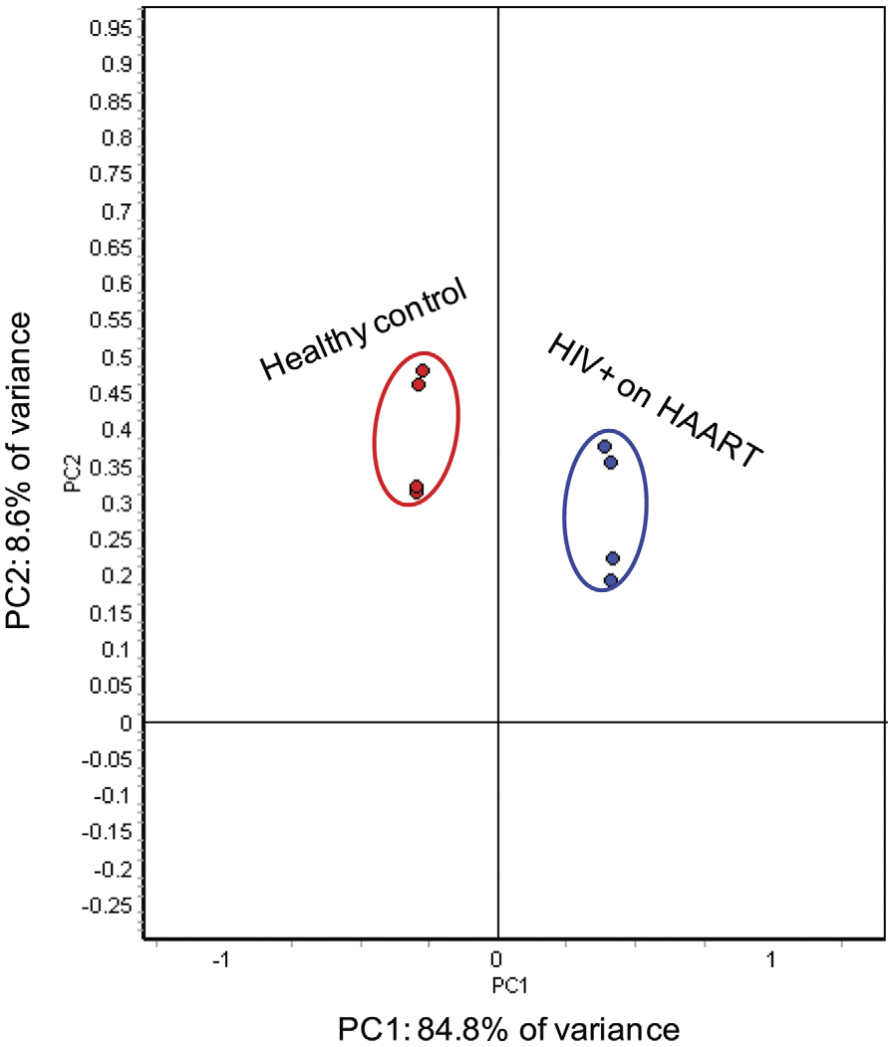
PCA of the proteins mediated by HIV and/or HAART. The protein expression profiles of experimental groups were visualized in two-dimensional Euclidian space, by using extended data analysis module of DeCyder software as described under “Materials and Methods.” The PCA distinctly clustered the 8 individual samples into two experimental groups (HIV-infected on HAART and healthy control subjects).

Global relationships among samples were visualized by performing a principal component analysis on the expression data (Figure 2). Before dimensional reduction, each spot map existed in multi-dimensional space (one dimension for each of the expression values for a spot). The spot map comparisons were plotted in two-dimensional space, corresponding to the first and second principal components of variation. The first principal component for each spot map is the weighted linear combination of intensity values that shows maximum variation, whereas the second principal component is a weighted linear combination orthogonal to the first component that has maximum variance. For the spots that are present in more than 80% of the gels, the 1^st^ principal component distinguished 84.8% of the variance with 8.6% additional variation distinguished by the second principal component. This unsupervised principal component analysis (PCA) demonstrated that the four technical replicate samples per experimental group generally segregated into two groups indicating that the variance contributed by the experiment is smaller than the variance between the two experimental groups. One would not expect the individual samples to cluster in a way our experimental groups are clustered, shown in Figure 2, if the fold changes for the individual proteins reported in Table S2 arose by chance. In this way, the PCA further demonstrated the statistical significance of the fold changes for the proteins reported in Table S2.

### Pathways related to protein abundance changes

**Figure 3:**
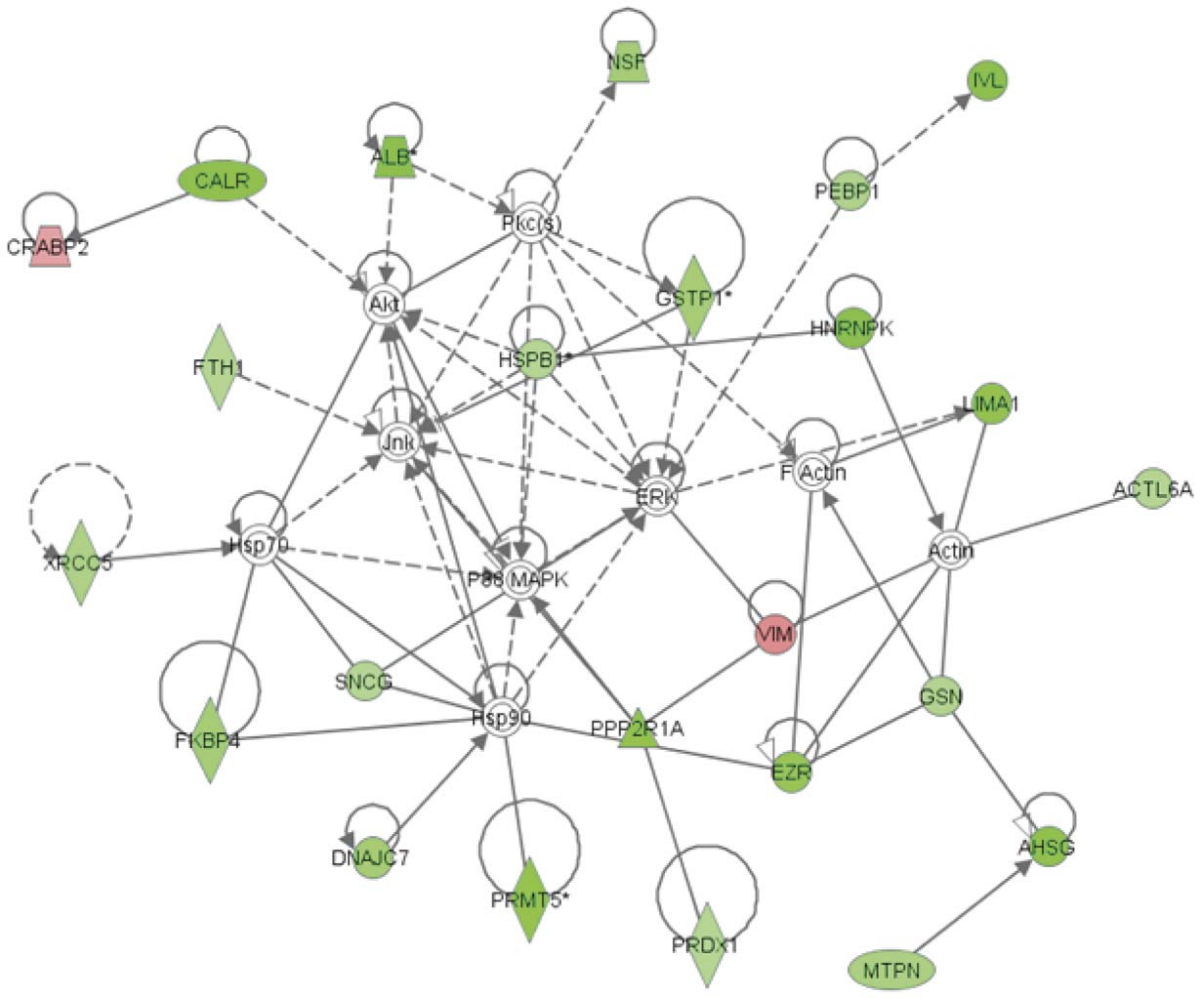
Protein networks associated with the proteins differentially expressed in response to HIV and/or HAART. The network was generated by Ingenuity pathway analysis (IPA) software using the list of differentially expressed proteins identified by 2-D DIGE/MS analysis. Individual proteins are represented as *nodes*, and the different *shapes* of the nodes represent the functional class of the proteins. The *edges* define the relationships of the nodes: the *arrowheads* indicate the direction of the interaction.

To understand the relationship of these proteins and the effects of these changes in the context of their cellular function, to identify potentially co-regulated partner proteins, and to ascertain their interactions with other proteins in known networks, the identified proteins were analyzed using a web-based entry tool developed by Ingenuity Systems, Inc. (http://www.ingenuity.com). Pathway analyses revealed that Nrf2 mediated stress response was the major pathway dysregulated in the comparison of experimental groups. Nuclear factor-erythroid 2 p45-related factor 2 (Nrf2) is a transcription factor which regulates the expression of many cyto-protective genes. In addition, lipid antigen presentation (relate to innate immunity), and acute phase response (related to inflammation) were additional major pathways dysregulated between the two groups (Table 2). These data clearly suggest that stress responses, innate immunity, and inflammation responses are dysregulated in HIV-infected on HAART HOECs, however these data lack molecular detail as to the sub-pathways that are specifically altered, which would provide specific biomarkers or molecular targets for therapy. To provide a greater degree of molecular detail, de novo network construction using IPA produced a sub-network of interactions shown in Figure 3 (with a P value of <10^−78^) that included almost 50% of the total proteins imported into IPA. The proteins in this network are annotated primarily with respect to three biological functions namely, protein folding, post-translational modification, and cell assembly and organization. On this network, nodes (proteins) with the red and green highlights are the specific proteins identified in the current study, while the nodes (proteins) not in color have been added by IPA to maximize the network connectivity. The red and the green colors represent the direction of change in protein expression, with red indicating up-regulated and green indicating down regulated proteins respectively in HIV-infected subjects as reported by 2-D DIGE. The edges with the arrowheads described the direct or indirect (solid or dotted line) nature of the interaction of these proteins. The solid line without the arrowheads refers to a known binding interaction based on the IPA database annotations. Every protein interaction in this network is based on published research articles. The interactions described by this network provide a framework for evaluating our targets, and enables us to identify significant new functional modules that are coordinately dysregulated. For instance, on the network it is clear that HOEC responses in these chronically ill HIV-infected subjects include suppression of protein modules that are essential for stress responses (such as heat shock proteins). It is also clear that IPA analysis predicts the involvement and activation of major signal transduction complexes in controlling the expression changes for many of the proteins such as the MAPK pathway. As proteins do not function in isolation, pathway analysis may help uncover the concerted changes of protein modules in revealing dysregulated host proteins that may contribute to prediction for secondary co-viral infections.

**Table 2:** Cellular pathways with greater-than-chance representation by signature proteins.

Pathways	Expectation
NRF2-mediated oxidative Stress Response	8.84E-04
Lipid Antigen Presentation by CD1	2.99E-03
Selenoamino Acid Metabolism	4.0E-03
Acute phase Response Signaling	5.68E-03
Pentose Phosphate Pathway	7.37E-03

#### Verification by Western blotting

**Figure 4:**
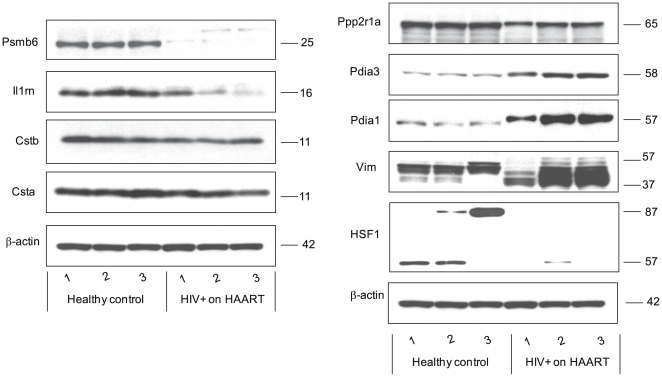
Protein abundance for representative proteins assayed by immunoblotting. Twenty micrograms of HIV-negative healthy control and HIV-infected on HAART subjects protein extracts was loaded per lane, each lane representing one of the three replicates per experimental group. The resulting blots were probed with antibodies that are specific to:- Ppp2ra1, PDIA3, PDIA1, Vim, PSMb6, IL1RN, CSTA, CSTB, and HSF1, as well as β-actin as a loading control. Relative -fold changes for these proteins in HIV-infected on HAART subjects compared to the healthy controls are given in Table 3. N1*_3*, healthy controls; H1*_3*, HIV-infected on HAART subjects (Table S1, subjects 5–7).

To verify both the direction and fold changes identified by the 2-D DIGE analysis, Western blot analysis was carried out for representative proteins on an independent set of twelve patient samples (six HIV-infected, Table S1, and six healthy controls) not used in the initial 2-D DIGE discovery experiments. The oral tissue for these experiments was also expanded to provide a homogeneous epithelial cell population (as for the discovery experiments) but the patient samples were not pooled, thus individual patient variation was assessed as well. In the first verification study using three HIV-infected subjects (Table S1, subjects 5–7) and three healthy controls, we chose to verify key pathway elements; these included protein disulfide isomerase precursor 1 and 3 (Pdia1, Pdia3), to measure responses to stress; interleukin 1 receptor antagonist isoform 1 (Il1nr), to measure inflammatory response; Cystatin A and B (Csta, Cstb), to measure protease inhibitor functions; Vimentin (Vim), to measure cytoskeleton integrity; Serine/threonine-protein phosphatase 2A 65 kDa regulatory subunit A alpha isoform (Ppp2r1a) to measure effects on signaling, cell growth and division; and proteasome subunit beta type-6 (Psmb6), to measure proteasome expression. The relative fold changes for these proteins were computed using ImageQuant-TL v2005 software and are summarized in Table 3 with statistical significance computed using student's T-test. Overall, our Western blotting results were in agreement with the directionality of the abundance changes reported by 2-D DIGE, with the exception of CstA (Figure 4 and Table 3). This protein exhibited a decrease in abundance in HIV-infected on HAART subjects as reported by 2-D DIGE analysis, but the decrease was not statistically significant with Western blotting (Table 3). Alternatively the results for CstB were in good agreement between the two approaches. For other proteins validated by Western blot, Il1rn showed close agreement by the two methods in terms of direction and fold change, while the fold changes for Ppp2r1a were lower by Western than 2D DIGE (−1.53 vs. −2.75), and the fold changes were much higher by Western for Psmb6 (−10.3 vs −2.6), although the direction of change was identical.

**Table 3:** Relative fold changes for selected proteins that were identified either by 2-D DIGE/MS and/or by network analysis as determined by verification immunoblot analysis.

Protein	HIV-infected/normal^a^	p-value (t-test)
Ppp2r1a	−1.53	0.0061
Pdia3	4.30	0.0013
Pdia1	4.46	0.0073
Psmb6	−10.3	0.0003
Il1rn	−2.30	0.0393
Vim (upper band)	3.28	0.0238
Vim (lower band)	6.93	0.0065
CstA	1.31	0.156
CstB	−1.36	0.031
HSF1	−12.5	0.0425

The mean values of the fold change and the t-test are based on the independent measurements from Figure 4.

a The fold changes is expressed as a ratio between the mean intensity of immunoblot for the HIV-infected on HAART and healthy controls after normalization to beta-actin.

In addition, the 2-D DIGE results for Pdia1, for which the spot was sequenced, were not considered conclusive and this protein was not reported in Table S2 as the relevant spot also included Tubb4. A Western blot analysis of Pdia1 identifies statistically significant four-fold induction of Pdia1 in HIV-infected on HAART subjects compared to the healthy controls. A major difference was also observed between HIV-infected on HAART subjects and healthy controls blots probed for vimentin (Figure 4). The intensities of the bands for full-length vimentin appeared to be similar for cell extracts from both HIV-infected on HAART and healthy control samples. However, very intense lower molecular weight products appeared in extracts from HOECs of HIV-infected on HAART subjects. The molecular weight ranges and the band patterns are similar to the band sizes and patterns reported for authentic primary and secondary vimentin cleavage products produced when vimentin is incubated with HIV-protease [23] or in cells transformed by sarcoma virus [24] and/or when astrocyte maturation is induced by thyroid hormone [25], or during oxidized low density lipoprotein induced macrophage apoptosis [26].

We observed decreases in the heat shock related proteins Hspb1, Hspd1, Hsp40, Hsp56 and Trap (Hsp90L) in HIV-infected subjects (Table S2) and pathway analysis suggested potential involvement of Hsp70 (Figure 3). To determine if the suppression of heat shock proteins may be mediated through a common transcriptional factor, we assessed the endogenous level of Heat shock factor protein 1 (Hsf1) in HOECs from HIV-infected and healthy controls. In eukaryotes, the key regulatory molecule in stress response is the heat shock transcriptional factor (HSF). There are multiple HSF isoforms, with HSF1 being a major regulator of heat shock protein transcription [27]. As clearly delineated in Figure 4, multiple isoforms of HSF1 (possibly unmodified, phosphorylated and/or sumolyated [28]) are observable in the normal subjects, while only the lower molecular weight form of HSF1 is seen, and only at a low level, in one of the HIV-infected subjects. The overall intensity reduction for the HIV-infected on HAART subjects with all bands integrated is 12.5 fold (p-value 0.04). The suppression of HSF1 in HIV-infected on HAART subjects compared to the healthy controls suggests that down regulation of the several heat shock proteins may be mediated through HSF1 repression. In unstressed cells, HSF1 exists as inactive monomer forming complexes with a variety of different multimeric proteins, many of which contain either HSP40/HSP70 or HSP90 [29], [30]. In response to stress or other stimuli the inactive HSF1 undergoes trimerization, and hyperphosphorylation, followed by nuclear localization and activation to elicit transcriptional activation of heat shock proteins [27]. Although HSF1 is essential to attenuate stress in chronic disease, our analysis of total HOEC lysates verifies significant losses of HSF1, reducing the cells' ability to respond to stress. The reduced levels of HSF1 may have influences beyond the heat shock/stress response of HOEC cells as HSF1 has many other non-stress targets of transcriptional activation, including cytokines and chemokines [31] highlighting the dysregulation of inflammatory pathways in the cells from HIV-infected on HAART subjects.

### Verification for Mitogen-Activated Protein Kinase pathway

**Figure 5:**
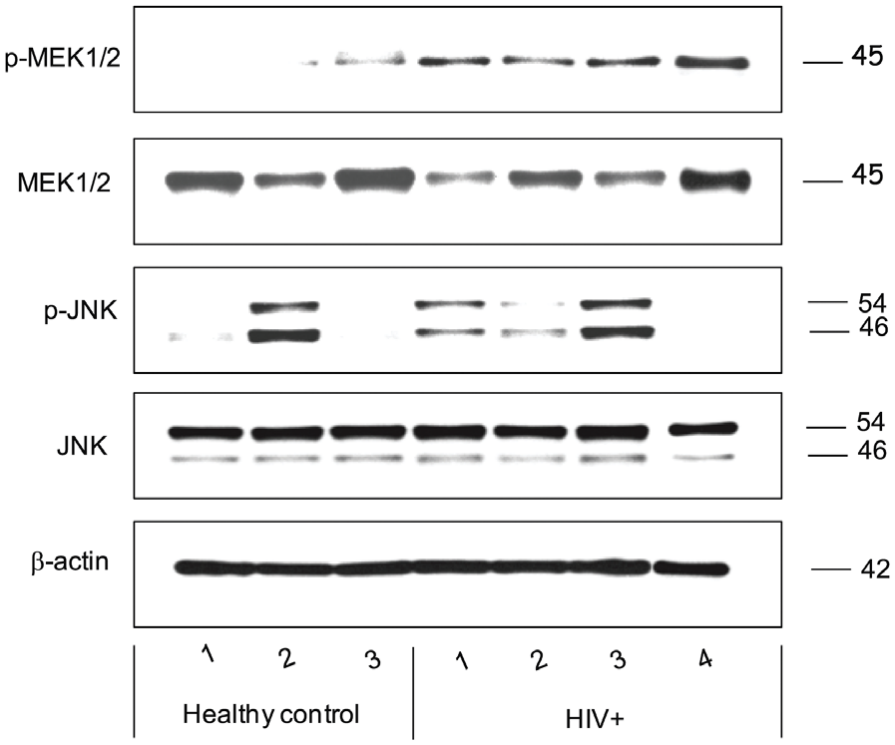
Endogenous levels of MEK1/2, JNK1/2, p-MEK1/2, and p-JNK, predicted to be activated by IPA, assayed by immunoblotting. The blots were probed with antibodies specific to MEK1/2 and JNK and phosphospecific antibodies to their phosphorylated counterparts in the total lysates indicated from left: Healthy controls (1–3); HIV+ (1–4), HIV-infected subjects, with subjects in HIV+ lanes 1 and 4 HAART naïve while 2 and 3 are on HAART (see also Table S1, subjects 8–11).

IPA predicted involvement of major signal transduction molecules that act as potential master regulators. To validate this prediction (Figure 3), we assessed the endogenous level of phospho- c-Jun N-terminal kinases (phospho-JNK), as it directly interacts with glutathione-S transferase 1 (GSTP1), one of our significantly down regulated targets, and phospho-MEK1/2 (MAP kinase kinase, the upstream activator of ERK) via Western blot on HOEC lysates from four HIV-infected patients; two on HAART (patients 9, 10; Table S1) and two HAART naïve (patients 8, 11; Table S1) and three healthy controls. Figure 5 and Table 4 show that HOEC lysates from HIV-infected subjects have 3.1-fold more phospho-MEK1/2 than those of the healthy controls (p-value 0.009) while total MEK is reduced (1.4-fold) but the change is not significant. Phospho-JNK is seen to be higher in HIV-infected patients but the change is not statistically significant while total JNK is unchanged. The ratios of p-MEK/MEK and pJNK/JNK were also examined; p-MEK/MEK increases five-fold in HIV infected patients (p value 0.05, data not shown) while the change in the ratio of p-JNK/JNK is not significant. The data from the HAART naïve patients 8 and 11 (HIV+, lane 1, 4) are not significantly different from the patients on HAART (data not shown). The p-JNK level is quite variable in the HIV-infected and control lysates and is likely influenced by other factors than purely HIV-infection.

**Table 4:** Relative fold changes assayed by Western blotting for representative proteins that were predicted to be activated by network analysis.

Protein	HIV-infected/normal^a^	p-value (t-test)
p-MEK1/2	3.13	0.0090
MEK	−1.37	0.3523
P-JNK-54	1.80	0.3446
P-JNK-46	1.24	0.7387
JNK-54	−1.03	0.6401
JNK-46	−1.09	0.4672

The mean values of the fold change and the t-test are based on the independent measurements from Figure 5.

a The fold changes is expressed as a ratio between the mean intensity of immunoblot for the HIV-infected on HAART and healthy controls after normalization to beta actin.

GSTP1 is decreased significantly in the HIV-infected discovery samples compared to controls and has been shown to bind and inhibit the activity of JNK [32], [33]. Activation of JNK would be consistent with suppression of GSTP1 in HIV-infected on HAART subjects. Usually, JNK activation is accompanied by ERK deactivation or vice versa. It has been well established that JNK plays a pro-apoptotic function in response to various cellular stress, whereas ERK is primarily involved in proliferative response [34], [35]. However, a recent study by Kins *et al* demonstrated a parallel activation of both JNK and ERK through PP2A inhibition [36]. From both 2-D DIGE and verification Western analysis on Ppp2r1a, we detected statistically significant suppression of Ppp2r1a, a regulatory sub unit of PP2A, in HIV-infected on HAART subjects compared to healthy controls. Thus, similar to the reports of Kins *et al*, phospho-MEK1/2 in diseased subjects suggests that activation of MEK1/2 could be mediated through suppression of Ppp2r1a.

## Discussion

HAART ameliorates several pathological features of HIV in human oral epithelial mucosa such as candidiasis. However, the prevalence of multiple comorbid related infections related to HIV do not necessarily appear to decrease concomitantly with HAART. An examination of molecular changes in oral tissue upon HIV infection and in the context of HAART is necessary to understand these comorbidities. In this study we have focused on proteomic expression changes in oral epithelia, to understand the possible mechanisms and/or host responses that are responsible for comorbid infections and we have identified changes in expression for multiple proteins of HIV-infected subjects compared to HIV- control individuals. Interestingly, many of these proteins have been shown to interact with HIV proteins and/or whose expression is HIV related [37], [38].

### Predominant protein down regulation themes

It is evident that a significant portion of the proteins we report in this study (Table 2), are down regulated in HIV-infected compared to HIV- control subjects. The molecular drivers of this down-regulation are of interest. The direct presence of virus in oral epithelial cells is debatable and has been addressed in only a few in *vitro* studies [39], [40], [41]. Although HIV is most likely not epitheliotropic, host infection with virus affects epithelial homeostasis and function in a number of ways. The epithelium is continually exposed to a number of oral pathogens and related stimuli. These stimuli include HIV-infected CD4^+^ T cells, monocytes, and macrophages, lymphocytes, and dendritic cells that come in contact with the epithelium. Upon host cell lysis, actively secreted HIV and HIV-induced pro-inflammatory cytokines are released. This state of chronic immune activation may mediate some or all of the changes in protein expression we see in these studies and may drive susceptibility to comorbid infections [42], [43].

At present we have no unbiased discovery proteomics data available from HOECs of HAART naive HIV-infected subjects. It is possible some of the down-regulation themes are due to the antiviral therapy [44], [45]. Thus, although the precise source of the dysregulation cannot be entirely established at this time, protein expression changes in separate HIV-infected on HAART HOEC subject samples were consistently observed. Moreover, we were able to show that for MAP kinase-kinase phosphorylation, the effects of HAART do not appear to be significant. Many of the down-regulated proteins have been linked to pro- and anti-inflammatory responses and oxidative stresses [46], [47]. The list includes, but is not limited to, proteins that are involved in protein folding and trafficking as well as in both pro- and anti- inflammatory responses (e.g., Hspb1, Dnajc7 (Hsp40), Fkbp4 (Hsp56), Hspd1 (Hsp60), Trap1 (Hsp90L), Calr, and Cryab2), proteins that are involved in redox homeostasis (e.g., Gstp1, Prdx1, and Ero1), and proteins that are involved in limiting the inflammatory responses (e.g.; IL-1RA, Galectin-3-binding protein, annexin III, and VI). Thus, the ability of oral epithelial cells of HIV-infected on HAART subjects to respond to stress is significantly attenuated compared to HIV- control subjects.

Heat shock proteins (Hsps) play an essential role as molecular chaperones by assisting the correct folding, assembly, and transport of proteins, as well as mediating degradation of polypeptides that are damaged due to various types of stress. Depending on the nature of the Hsp itself, the source of the accompanying stress related antigen, the local concentration of the Hsp, the nature and concentrations of other Hsps and the microenvironment in which the Hsp is encountered, these proteins can mediate both pro- and anti-inflammatory immune responses [46], suggesting that these molecules play a key role in the maintenance of immunological homeostasis. Heat shock proteins exhibit pro-inflammatory functions when they are expressed and released from cells where damage occurs and are exported either to the cell surface or to extra-cellular body fluids including blood [47]. Under normal physiological conditions, Hsps induce pro-inflammatory adaptive immune responses. A current report by Kebba *et al*, has shown significantly higher surface expression of heat-shock protein receptor CD91 on monocytes of HIV infected, long-term non progressors, suggesting that HIV antigen uptake and cross-presentation mediated by CD91 may contribute to host anti-HIV defenses and play a role in protection against HIV infection [48]. The concurrent suppression of a number of heat shock proteins, heat shock factor protein 1(HSF1), and known anti-inflammatory responses of the immune system, such as interleukin 1 receptor antagonist, and galectin-3-binding protein in oral epithelium of HIV chronically infected subjects, suggest that a sustained presence of HIV infection eventually leads the immune system into a state of immunological tolerance towards multiple antigens. A similar pattern of protein expression change is found in HIV-infected on -HAART subject samples used for verification experiments. This result supports the notion that HAART only provides partial immune recovery, especially to the peripheral but not to the local immune system, in HIV-infected individuals. The observed suppression of these immune modulators could also be caused by prolonged usage of HAART. In this regard, it will be interesting to examine the expression status of heat shock proteins in HAART naive HIV-infected subjects. Furthermore, our results also point to the crucial role for local, oral mucosa-specific, innate-immune functions in controlling comorbid infections.

Suppression of proteins that are involved in limiting inflammation, adds to the theme of chronic inflammation and immune activation. For example, decreased calreticulin precursor (Calr) and annexin III and IV play into pro-inflammation, as less Calr mediates less nuclear export of nuclear receptor subfamily 3, group C, member 1 (NR3C1); an important receptor that glucocorticoids bind to, in order to dampen inflammation [49]. Moreover, suppression of annexin III and IV, further support this theme, as annexins suppress phospholipase A2 which suppresses arachidonic acid release, resulting in less prostaglandin and leukotriene expression [50], [51]. Therefore, less annexin means more prostaglandin and leukotriene expression leading to increased inflammation. Overall, we see multiple examples of protein abundance changes consistent with a higher baseline inflammation level in HIV-infected on HAART HOECs than in controls.

In addition to heat shock proteins and anti-inflammatory proteins being suppressed in the HIV-infected subjects' oral epithelium, proteins associated with oxidative stress such as Gstp1, Prdx1, and Ero1 are also down regulated. The first two are antioxidant enzymes; i.e., they prevent the buildup of reactive oxygen species (ROS). Interestingly, it was recently reported that individuals on HAART have increased levels of ROS, such as glutathione peroxidase (Gpx), in their circulation, with concomitant decreased levels of antioxidants such as glutathione reductase, over time [52]; a clinical finding that supports our conclusions. Reduced levels of antioxidant enzymes and/or oxidative stress, in general, are known to up-regulate inflammatory cytokine activities in individuals receiving HAART [53]. The observed suppression of oxidoreductase enzymes known to be induced by hypoxia, such as Erol1 [54], [55], as we report herein, suggests an increased presence of ROS in oral epithelium, which is consistent with a significant increase in ROS in the circulation of HIV infected individuals on HAART. It is generally accepted that ROS are a source of oxidative stress that can adversely affect the immune response and activate certain latent viruses [56], [57], thereby predisposing the epithelium to secondary infections.

GSTP1, in addition to being associated with phase II detoxification of the products of oxidative stress, has recently been implicated in the regulation of cell proliferation and apoptosis through direct interaction of c-Jun N-terminal kinase, (JNK) [32], [58]. Furthermore, MAPK and upstream MAPK kinase dephosphorylation has been attributed to PP2A [36], [59], [60], in addition to a unique family of dual specific MAP kinase phosphatases [35]. In agreement with these reports, the suppression of Ppp2r1a in HIV-infected subjects may regulate the catalytic activity of PP2Ac, which in turn may contribute to the activation of not only JNK but also MEK1/2, a kinase for ERK. Although such a mechanism is consistent with the data, it represents only one plausible mechanism linking the possible pathways that are common for the activation of both JNK and ERK.

### Protein up regulation themes

The proteins that are up regulated in HIV-infected subjects include proteins that are involved in redox homeostasis such as protein disulfide-isomerase A1 precursor (Pdia1), and protein disulfide-isomerase A3 precursor (Pdia3). Although endoplasmic reticulum-resident proteins, protein disulfide isomerases (PDIs) are commonly detected at cell surfaces, and changes in the expression of intracellular PDIs are reflected in the their expression on cell surfaces [61]. These increases in Pdia1 and Pdia3, with a corresponding decrease in ER oxidoreductin (Ero1l alpha) are consistent with antioxidant imbalances associated with HIV-infection and a potential prolonged usage of HAART [52]. The decrease in the level of Ero1l alpha in HIV-infected subjects is probably attributed to the increases in the ROS in the ER. The Ero1l alpha enzyme is a flavoprotein that can couple the introduction of disulphide into the PDI with the reduction of oxygen to liberate hydrogen peroxide. For every disulphide introduction into PDI, one molecule of hydrogen peroxide can theoretically be produced. The production of this source of reactive oxygen species leads to ER-generated oxidative stress. On the other hand, PDIs have a direct role in the antioxidant system and mainly participate in regenerating peroxiredoxins that are oxidized by peroxides [62]. Thus, the induction of both Pdia1 and Pdia3 in HIV-infected on HAART subjects could have a protective role by avoiding the deleterious accumulation of ROS in the ER, and maintaining a physiological balance between the redox systems. Therefore, the increase in the expression of PDIs and a concomitant decrease in the expression of Erol is consistent with a protective response to the oxidative stress associated with the down-regulated families of antioxidant enzymes that play an important role in redox regulation and detoxification in these cells.

Another notable finding is accumulation of vimentin degradation products in HOECs of HIV-infected individuals. Western blot analysis of this protein (Figure 4), identified both quantitative and qualitative changes for vimentin in HIV-infected subjects, in particular the appearance of vimentin degradation bands. Muller *et al* reported major cleavage products (spanning from 46–57 Kda and 29, and 26 Kda) of vimentin during oxidized low density lipoprotein induced macrophage apoptosis [26]. Since HAART has been associated with increased levels of LDL and ROS in HIV-infected on HAART subjcets, in parallel with down regulation of a group of proteins that decrease apoptosis induced by ROS; i.e., Prx1, Gstp1, Cryab Hspd1, Hpb1, Lgals3bp, Ox-LDL mediated apoptosis of HOECs, may lead to vimentin fragmentation. Of note, HIV protease fragments vimentin both *in vivo* [63] and *in vitro* [23], and the number and size of vimentin fragments reported are also very similar to our Western blot results. While HIV may not infect epithelial cells efficiently (e.g. abortive infection may be a feature of the disease related effects), we cannot entirely rule out that HIV protease is present in the local epithelial environment during HIV permissive cell lysis.

Although our study identifies multiple pathways and proteins dysregulated in HIV-infected on -HAART subjects, some caveats must be admitted as this research is still in its early stages. First, the study only comprises a total of twenty-one subjects, with only eight individuals in the discovery phase. In addition, there is a systematic difference in the average age of the HIV-infected and control groups, as age-matched HIV negative volunteers undergoing the same surgical procedures were quite difficult to enroll. Thus, it is entirely possible that this sub-group of subjects may not broadly reflect the molecular alterations of a larger patient population and that important HIV related pathways may have easily been overlooked. Also, some of the differences may be attributable to age differences alone. However, the subjects are all adults from 18–58 years, which is an age range unlikely to be biased by senescence.

Despite the potential limited discovery set, the verification experiments, which were conducted on individual (un-pooled) patient samples, entirely confirmed the pathways and proteins identified in the initial discovery phase. While intact oral mucosa is composed of multiple cell types; these experiments utilized a homogeneous epithelial cell population, in order to provide results that could be unambiguously attributed to this cell type. Thus, additional studies, either by Western or preferably by immuno-histochemistry, performed on primary oral tissue, will be of interest to fully understand the protein dysregulation of the oral mucosa associated with HIV-infection on HAART. Furthermore, additional studies on HIV-infected, HAART naive subjects are needed to dissect the effects of HIV from those of HAART as we have on-HAART vs HAART naïve data only for MAP kinase kinase phosphorylation and for only two patients in each of these groups. Lastly, these studies suggest the potential for epigenetic variation, if not silencing in the oral epithelia, mediated by HIV or HAART related chronic effects. This is related to the interesting phenomena that despite the primary cellular expansion of the oral epithelial cells, the resultant cell populations represented by the HIV-infected vs. non infected groups have considerable variation in proteome expression. Silencing of oral cells in DNA damage and cancer is a theme of increasing interest, as is epigenetic silencing in general [64], [65], and future experiments to examine histone modifications or promoter methylation changes are certainly of interest to further explore the molecular basis of these differences.

In summary, our study has identified over 60 candidate proteins whose expression is dysregulated in HOECs of HIV-infected on HAART subjects and changes in the expression levels of these proteins could negatively impact local epithelial immunity. These proteins represent potential biomarkers for diagnostic and prognostic purposes and the dysregulated pathways are potential targets for novel therapies to combat co-infection risks. In addition, these biomarker proteins may be detectible in saliva, which is likely to contain significant quantities of oral tissue related proteins. Salivary biomarkers may reflect changes in oral pathology related to these findings and may represent an accessible oral fluid suitable for more routine diagnostic analysis.

Overall, the data suggests that local immune dysregulation in HIV-infected individuals involves down regulation of proteins which are necessary to attenuate cellular stress, to induce pro-and anti-inflammatory responses, and proteins which regulate ROS. The down regulation of these important clusters of proteins and pathways identified in this study may represent global epithelial defects that may contribute to increased susceptibility to infection observed in chronically ill HIV-infected subjects. While evaluation of the proteome expression in the two experimental groups does not distinguish protein expression patterns that may be due to HAART from those that are associated with chronic HIV infection, it does provide leads to establish association between the observed protein changes and epithelium susceptibility towards comorbid infections.

## Materials and Methods

### Reagents

The majority of chemicals in this study were obtained from GE Healthcare (Piscataway, NJ), Thermo scientific (Rockford, IL), and Invitrogen (Carlsbad, California) and used without further purification unless otherwise stated. Antibodies to Ppp2r1a, Psmb6, and β-actin, loading control (Abcam, Cambridge, MA) and antibodies to Pdia1, Pdia3, Vim, Il1ra, HSF1, CstA, and CstB (Sigma Aldrich, St. Louis, MO), MEK1/2, Phospho-MEK1/2, JNK, Phospho-JNK (Cell Signaling Technology, Danvers, MA) were purchased from the indicated vendors.

### Ethics Statement

Volunteers have been used as the source of material for the described work outlined in this manuscript. Human gingival tissue overlying impacted third molars from HIV-infected and healthy control subjects were collected after written informed consent was provided by study participants and/or their legal guardians. University Hospital Case Medical Center Institutional Review Board (IRB) Protocol #: 19981017 approved this study.

### Clinical Samples

Human gingival tissue overlying impacted third molars were obtained from HIV-infected subjects (48.0±8.7 years) and healthy controls (22.9±6.5 years). Both pretreatment CD4 T-cells data (nadir CD4) and CD4 T-cells after HAART at the closest date to tissue collection, as well as viral load per ml of blood from the HIV-infected subjects were determined (Table S1). Also, no diagnosis of gingivitis; i.e., inflammation of the gingival tissue, or periodontitis; i.e., alveolar bone loss, was observed in the biopsy sites from healthy or HIV-infected subjects.

### Human Oral Epithelial Cell (HOEC) preparation

Primary human oral epithelial cells (HOECs) were isolated and expanded in serum free keratinocyte growth medium with supplements as previously described by Krisanaprakornkit *et al* [66], [67]. Briefly, the epithelial layer was separated from the underlying fibrous connective tissue with dispase. A single cell suspension was prepared from the epithelial sheets by trypsinization and repeated pipetting. Cells were suspended in serum-free EpiLife media (Cascade Biologics Inc, Portland, OR), and plated on 10 cm petri dishes and grown to near-confluence. After reaching >80% confluence, cells were detached from the petri dish, pelleted, frozen and stored in liquid nitrogen until used for expression proteomics and verification analyses. Samples for the original 2-D DIGE analyses were pooled HOECs from 4 HIV-infected (Table S1, subjects 1–4) and 4 HIV- negative individuals respectively, while confirmation analyses were conducted on separate HOECs from HIV-infected (Table S1, subjects 5–11) and healthy control subjects.

### Sample size and Power analysis

To estimate sample size for this study power analysis was performed using two datasets from previous 2D-DIGE experiments [20], and (Yohannes et al, unpublished data). One of the datasets is from technical replicate gel images that were run using the same sample but labeled with either Cy3, or Cy5. The second dataset is from biological replicate gel images. Standardized volume of protein spots were exported into excel using XML Toolbox within DeCyder software. Once spots that are present across all the gel images were filtered, the within group variance for each spot followed by upper quartile variances were computed on a log transformed spot standardized volumes. Power for different sample size at 0.05 or 0.001 significant level, and using upper quartile variance and 50% change in expression was computed via Power and Sample Size Calculator Version 3.0.43 (a free package: http://biostat.mc.vanderbilt.edu/twiki/bin/view/main/PowerSampleSize). The relationship between power and sample size was plotted in Microsoft Excel.

### Protein extraction and fluorescence dye labeling

Sample preparation was conducted as previously described [20]. Briefly, total protein was harvested from the respective HOEC samples. Protein clean up and concentration of each sample was determined using a 2D cleanup and quant kits (GE Healthcare), respectively. For 2D DIGE experiments, four biological replicates from control and four from HIV-infected subjects were pooled in order to minimize interpersonal variation as well as meet the minimum total protein sample size required to carry out the experiment. The samples were then split into four 50 µg aliquots. An additional sample, a pool of 100 µg protein from each group was collected from HIV-infected and normal samples (200 µg total) and split into four 50 µg aliquots and used as an internal standard. Thus, samples from either HIV-infected or healthy control were labeled with Cy3 or Cy5 cyanine dyes alternatively while the internal standard samples were labeled with Cy2 dye by the addition of 400 pmol of Cy dye in 1 µL of anhydrous N,N dimethylformamide per 50 µg of protein. A dye-swapping scheme, as shown in Table 1 was used such that the four samples for any condition were variously labeled with Cy3 or Cy5 to control for any dye-specific labeling artifacts. Labeling was performed for 30 min on ice in the dark; the reaction was then quenched with 10 mM lysine and additionally incubated for 10 min.

### Gel electrophoresis

The quenched Cy3 and Cy5-labeled samples, to be partitioned in the same gel according to the experimental design in Table 1, were then combined and mixed with an aliquot of Cy2-labeled internal standard and an equal volume of 2× sample buffer (8 M urea, 4% w/v CHAPS, 2% w/v dithiothreitol (DTT), 2% v/v Pharmalytes 3–10 NL) was added. Prior to isoelectric focusing (IEF), an additional 350 µg of unlabeled protein was added (for later spot picking and protein identification) and the mixtures were brought up to 450 µl with 1× rehydration buffer. The mixed samples were then partitioned according to their isoelectric point (pI) and molecular weight, in two dimensional gels, as previously described [20].

### Image acquisition and spot quantification

Gel images, acquisition and spot quantification were carried out as previously described [20]. Briefly, data analysis was carried out using a total of 12 gel images consisting of four replicate images from healthy control, four replicates of HIV-infected and four from the internal standards, which were pooled mixtures of equal aliquots of each experimental sample. The pick gel image was also processed with the rest of the gel images but not included in the analysis. In order to compare protein spots across the eight gel images, image analyses were conducted in two steps using DeCyder v6.5 2D differential analysis software (GE Healthcare). In the first step, the set of three images from a single gel were loaded into differential in-gel analysis (DIA) module within the DeCyder software to detect and quantify intra-gel spots. For the subsequent inter- gel differential analysis, the DIA workspaces for all the gels were saved and loaded into the biological variation analysis (BVA). In the BVA module, the image with the largest number of protein spots was assigned as the master image. Sample (Cy3 or Cy5 labeled) spot maps were assigned into healthy control, HIV-infected and all internal standard and the pick spot maps were assigned into standard and pick folders, respectively, in the experimental design view of the BVA modules. Once the spots from the common standards were matched across the analytical gel images and with the pick gel image, the standardized volume ratio for each standard image from the different gels was set to the value 1.0 in order to compare ratios between matched protein spots in the different gels (groups). Thus, the ratios of the log standardized protein spot abundances (differences in expression) between the groups were computed.

To test for significant differences in expression of proteins between the two experimental groups, T-test was performed at a significance level of 0.05; thus, for every hundred spots tested, five false positives would be expected. Specifically, the data were filtered using the average volume ratios of 1.5 and above or −1.5 and below fold differences in expression and with false discovery rate adjusted (FDR) T-test p value of 0.05 or less and assigned as a spot of interest. For the spots that displayed significant differences in expression between the groups, a pick list with pick location and coordinates were generated. The pick list along with the post-stained pick gel was transferred to the automated Ettan spot picker and excised gel plugs were placed into a 96-well plate for the subsequent in-gel digestion and mass spectrometry analysis of the peptide for protein identification.

Multivariate analyses were performed on the expression data derived from the BVA using the DeCyder extended data analysis (EDA) software. The gel images were grouped such that the 4-technical replicates for each experimental condition formed a group. Once the BVA was imported into EDA, the data was filtered so that only 153 spot features exhibiting statistically significant (T-test p<0.05) changes and present in more than 80% of the spot maps were considered. The global relationships among spot maps were visualized by performing a principal component analysis (PCA) on these spot features.

### In gel digestion and protein identification

The proteins in the gel plugs were digested with trypsin (Promega) using a modified protocol adapted from Shevchenko *et al.* [68]. Tryptic digests were extracted from the gel matrix, concentrated by SpeedVac avoiding complete drying, and reconstituted in 0.1% formic acid. The protein digests were then trapped onto a pre-column (C18, PepMap100, 300 µm ×5 mm, 5 µm particle size, 100 Å, Dionex) and desalted on-line in mobile phase A (0.1% formic acid in 5% acetonitrile) at 10 µL/minute for 10 minutes using a Dionex Ultimate 3000 capillary LC system on line with an LTQ- Fourier Transform (FT) mass spectrometer (Thermo Electron Corp., Bremen, Germany). Each sample was subsequently loaded onto a Dionex C18 PepMap 75 µm×15 cm reversed phase column with 5% mobile phase B (0.1% formic acid in 85% acetonitrile). Separation of peptides was obtained with a linear gradient of 2% per min, starting with 100% of mobile phase A. Subsequently, the peptides were infused at a flow rate of 300 nL/min via PicoTip emitter (New Objective Inc., Woburn, MA) a voltage of 1.8 kV into LTQ-FT mass spectrometer to acquire mass spectrum data (alternating full MS scan and MS/MS scans). Survey data was acquired from m/z of 400 to 1600 and the precursors were interrogated by MS/MS per switch. The switch into MS/MS was based on precursor intensity and 5 scans were acquired for every precursor interrogated.

The tandem mass spectra were annotated and peak list files were generated, commonly referred to as. DTA files, by running SEQUEST extract_msn algorithm in Bioworks version 3.2 (Thermo Electron, Bremen, Germany). The mass range m/z 400–3500 from full scan data, with an absolute threshold of 100, a minimum ion count of 12 and a precursor ion tolerance of 1.4 Da were used to generate the .DTA file. Prior to searching, non-redundant databases of 3,893,302 sequences (release 07/04/06) were downloaded in FASTA format via file transfer protocol (FTP) from the website of the NCBInr. These databases were stored locally and a subset human database was created and indexed to produce faster search times. The resulting peak list (.DTA) files were then used to interrogate sequences present in an indexed human subset database (137,607 sequences stored locally) by running SEQUEST SEARCH algorithm of Bioworks software version 3.2. SEQUEST searches were performed with maximum peptide and fragment ion mass tolerance of 2.5 and 1.0 Da respectively, with partial methionine oxidation, and complete carbamidomethylation of cysteine. Two missed cleavage sites were also allowed in the search parameters. The criteria for each protein identification was a minimum of two peptides with a significant peptide expectation (P<0.001), peptide Xcorr 1.9, 2.7, and 3.5 for the charge states and +1, +2, and +3 respectively, and a minimum Delta CN (Delta correlation) of 0.1. The correlation of theoretical molecular weight and pI with the gel region were also generally considered. In addition all the MS/MS spectra identified by SEQUEST were manually verified for spectral quality and matching y and b ion series (A complete protein list and annotation criteria are included in the Table S3).

### Network Analysis

The data set with a list of regulated proteins identified by 2-D DIGE was analyzed further using the network building tool Ingenuity Pathway Analysis (Ingenuity Systems, Inc.). The Ingenuity Pathways Knowledge Base is the largest curated database of previously published findings on mammalian biology from the public literature (Ingenuity Systems). The databases consist of millions of relationships between proteins that are derived from published data on proteins and small molecules. The relationships include direct protein interaction, transcriptional regulation, binding, enzyme-substrate and other structural or functional relationships. The networks can be visualized graphically as nodes (proteins) and edges (the relationship between proteins) alongside empirical expression patterns. The data set from our study was imported into the IPA systems. Hypothetical networks of proteins from our experiment and proteins from the Ingenuity systems database were then built using the *de novo* network building algorithm, one of the several algorithms integrated within Ingenuity systems.

### Western blotting

Cell lysates were prepared from HOECs obtained from an independent set of biological replicates (individuals not used previously for the initial 2-D DIGE experiment) from healthy controls and HIV-infected on -HAART or HAART naïve subjects. Twenty micrograms of protein extracts from all subjects was loaded per lane, each lane representing one of the three or four replicates per experimental group. The proteins were then resolved by electrophoresis on denatured and reduced 4–12% polyacrylamide gels (Invitrogen) and transferred onto nitrocellulose membranes for western blotting. The membranes were blocked with 5% skim milk or 5% BSA for 1 hr at room temperature with gentle shaking, washed, and incubated overnight with specific primary antibodies against Pdia1 (0.5 ng/µl), Pdia3 (0.130 ng/µl), Il1nr (0.120 ng/µl), Cstb (0.130 ng/µl), CstA (0.080 ng/µl), Vim (0.170 ng/µl), Ppp2r1a (1.000 ng/µl), Psmb6 (1.000 ng/µl), total JNKs (0.180 ng/µl) or dual phosphor-JNKs, (0.150 ng/µl), MEK1/2 (0.017 ng/µl) or phosphor-MEK1/2 (0.066 ng/µl), HSF1 (0.080 ng/µl) and β-actin at (1.000 ng/µl). The membrane was washed and then incubated for 1 hr at room temperature with horseradish peroxidase (HRP)-conjugated secondary antibody (2 ng/µl). After three washes, the blot was incubated for 5 minutes in chemiluminescent developing substrate, prepared according to the manufacturers' instructions (Pierce Biotechnology; Rockford, IL or Cell Signaling Technology; Danvers, MA). The membrane was removed from the substrates and placed in plastic sheet protectors and the chemiluminescence was visualized by exposing the blot to Kodak X-OMAT. The bands on the films were scanned quantified using ImageQuant-TL v2005 software (GE Healthcare, Piscataway, NJ). Equal loading of the protein samples was demonstrated by re-probing the blots for β-actin (Figure 4 and 5). In data analysis the intensity of each band was normalized using the β-actin intensity.

## Supporting Information

Figure S1 **The relationship between power and sample size at α = 0.05 or 0.001 for 50% change in protein expression.** Two datasets from technical replicates and biological replicates encompassing technical noise only and both technical and biological noises respectively were used to estimate the upper quartile variances and to compute power at various sample size.
(TIF)

Table S1 **Clinical data of the HIV-infected subjects used for 2-D DIGE and/or validation experiments.** The samples from the first 4 subjects, who are HIV-infected on HAART, were pooled for the 2-D DIGE analysis. The subject samples 5–11 were used for the verification analysis. Samples from HIV-infected on HAART subjects 5, 6,and 7 were presented in the Western blot analyses of Figure 4. Samples from subjects 8, 9, 10, and 11 were used for the Western blot analyses presented in Figure 5. Patients 8 and 11 are HAART naïve while 9 and 10 are on HAART. ^1^The mean viral load for subjects 5–11 does not include subjects 8 and 11, who were HAART naïve.
(DOC)

Table S2 **Differential proteome profile of oral epithelial form HIV-infected on HAART subjects.**
(DOC)

Table S3 **Database Search** **Results** **of spectra acquired on a Fourier Transform LTQ Mass Spectrometry.** The tandem mass spectra were annotated and generated peak list files (.DTA), by running SEQUEST extract_msn algorithm in Bioworks version 3.2 (Thermo Electron, Bremen, Germany). The resulting peptide mass lists were then used to interrogated sequences present in an indexed human subset database (137,607 sequences), that was created from NCBInr 3,893,302 sequences (release 07/04/06) and stored locally, by running SEQUEST SEARCH algorithm of Bioworks software version 3.2. SEQUEST searching were performed with maximum peptide and fragment ion mass tolerance of 2.5 and 1.0 Da respectively, and with partial methionine oxidation (M)and complete carbamidomethylation of cysteine (C), and 2 missed cleavage sites were also allowed in the search parameter. For each protein identification, a minimum of two peptides with a significant peptide expectation (P<0.001), peptide Xcorr 1.9, 2.7, and 3.5 for the charge states and +1, +2, and +3 respectively, minimum Delta CN (Delta correlation) of 0.1.
(DOC)

## References

[1] Greenspan D, Greenspan JS (1996). HIV-related oral disease.. Lancet.

[2] Leao JC, Ribeiro CM, Carvalho AA, Frezzini C, Porter S (2009). Oral complications of HIV disease.. Clinics (Sao Paulo).

[3] Nicolatou-Galitis O, Velegraki A, Paikos S, Economopoulou P, Stefaniotis T (2004). Effect of PI-HAART on the prevalence of oral lesions in HIV-1 infected patients. A Greek study.. Oral Dis.

[4] Bedimo R (2008). Non-AIDS-defining malignancies among HIV-infected patients in the highly active antiretroviral therapy era.. Curr HIV/AIDS Rep.

[5] Baumgarth N, Szubin R, Dolganov GM, Watnik MR, Greenspan D (2004). Highly tissue substructure-specific effects of human papilloma virus in mucosa of HIV-infected patients revealed by laser-dissection microscopy-assisted gene expression profiling.. Am J Pathol.

[6] Greenspan D, Canchola AJ, MacPhail LA, Cheikh B, Greenspan JS (2001). Effect of highly active antiretroviral therapy on frequency of oral warts.. Lancet.

[7] Greenspan D, Gange SJ, Phelan JA, Navazesh M, Alves ME (2004). Incidence of oral lesions in HIV-1-infected women: reduction with HAART.. J Dent Res.

[8] Hodgson TA, Greenspan D, Greenspan JS (2006). Oral lesions of HIV disease and HAART in industrialized countries.. Adv Dent Res.

[9] Cauda R, Tacconelli E, Tumbarello M, Morace G, De Bernardis F (1999). Role of protease inhibitors in preventing recurrent oral candidosis in patients with HIV infection: a prospective case-control study.. J Acquir Immune Defic Syndr.

[10] De Bernardis F, Tacconelli E, Mondello F, Cataldo A, Arancia S (2004). Anti-retroviral therapy with protease inhibitors decreases virulence enzyme expression in vivo by Candida albicans without selection of avirulent fungus strains or decreasing their anti-mycotic susceptibility.. FEMS Immunol Med Microbiol.

[11] Volter C, He Y, Delius H, Roy-Burman A, Greenspan JS (1996). Novel HPV types present in oral papillomatous lesions from patients with HIV infection.. Int J Cancer.

[12] Chaturvedi AK, Madeleine MM, Biggar RJ, Engels EA (2009). Risk of human papillomavirus-associated cancers among persons with AIDS.. J Natl Cancer Inst.

[13] Grulich AE, van Leeuwen MT, Falster MO, Vajdic CM (2007). Incidence of cancers in people with HIV/AIDS compared with immunosuppressed transplant recipients: a meta-analysis.. Lancet.

[14] Besson C, Goubar A, Gabarre J, Rozenbaum W, Pialoux G (2001). Changes in AIDS-related lymphoma since the era of highly active antiretroviral therapy.. Blood.

[15] Strickler HD, Burk RD, Fazzari M, Anastos K, Minkoff H (2005). Natural history and possible reactivation of human papillomavirus in human immunodeficiency virus-positive women.. J Natl Cancer Inst.

[16] Clifford GM, Goncalves MA, Franceschi S (2006). Human papillomavirus types among women infected with HIV: a meta-analysis.. Aids.

[17] Carter MM, Torres SM, Cook DL, McCash CL, Yu M (2007). Relative mutagenic potencies of several nucleoside analogs, alone or in drug pairs, at the HPRT and TK loci of human TK6 lymphoblastoid cells.. Environ Mol Mutagen.

[18] Torres SM, Walker DM, Carter MM, Cook DL, McCash CL (2007). Mutagenicity of zidovudine, lamivudine, and abacavir following in vitro exposure of human lymphoblastoid cells or in utero exposure of CD-1 mice to single agents or drug combinations.. Environ Mol Mutagen.

[19] Nibbe RK, Markowitz S, Myeroff L, Ewing R, Chance MR (2009). Discovery and scoring of protein interaction subnetworks discriminative of late stage human colon cancer.. Mol Cell Proteomics.

[20] Yohannes E, Chang J, Christ GJ, Davies KP, Chance MR (2008). Proteomics analysis identifies molecular targets related to diabetes mellitus-associated bladder dysfunction.. Mol Cell Proteomics.

[21] Calvano SE, Xiao W, Richards DR, Felciano RM, Baker HV (2005). A network-based analysis of systemic inflammation in humans.. Nature.

[22] Kulkarni H, Okulicz JF, Grandits G, Crum-Cianflone NF, Landrum ML Early Post-Seroconversion CD4 Cell Counts Independently Predict CD4 Cell Count Recovery in HIV-1-Postive Subjects Receiving Antiretroviral Therapy.. J Acquir Immune Defic Syndr.

[23] Shoeman RL, Honer B, Stoller TJ, Kesselmeier C, Miedel MC (1990). Human immunodeficiency virus type 1 protease cleaves the intermediate filament proteins vimentin, desmin, and glial fibrillary acidic protein.. Proc Natl Acad Sci U S A.

[24] Singh B, Arlinghaus RB (1989). Vimentin phosphorylation by p37mos protein kinase in vitro and generation of a 50-kDa cleavage product in v-mos-transformed cells.. Virology.

[25] Paul S, Gharami K, Das S, Sarkar PK (1999). Thyroid hormone-induced maturation of astrocytes is associated with the expression of new variants of vimentin and their phosphorylation.. J Neurochem.

[26] Muller K, Dulku S, Hardwick SJ, Skepper JN, Mitchinson MJ (2001). Changes in vimentin in human macrophages during apoptosis induced by oxidised low density lipoprotein.. Atherosclerosis.

[27] Sarge KD, Murphy SP, Morimoto RI (1993). Activation of heat shock gene transcription by heat shock factor 1 involves oligomerization, acquisition of DNA-binding activity, and nuclear localization and can occur in the absence of stress.. Mol Cell Biol.

[28] Holmberg CI, Hietakangas V, Mikhailov A, Rantanen JO, Kallio M (2001). Phosphorylation of serine 230 promotes inducible transcriptional activity of heat shock factor 1.. EMBO J.

[29] Zou J, Guo Y, Guettouche T, Smith DF, Voellmy R (1998). Repression of heat shock transcription factor HSF1 activation by HSP90 (HSP90 complex) that forms a stress-sensitive complex with HSF1.. Cell.

[30] Bharadwaj S, Ali A, Ovsenek N (1999). Multiple components of the HSP90 chaperone complex function in regulation of heat shock factor 1 In vivo.. Mol Cell Biol.

[31] Inouye S, Izu H, Takaki E, Suzuki H, Shirai M (2004). Impaired IgG production in mice deficient for heat shock transcription factor 1.. J Biol Chem.

[32] Adler V, Yin Z, Fuchs SY, Benezra M, Rosario L (1999). Regulation of JNK signaling by GSTp.. EMBO J.

[33] Kim YJ, Lee WS, Ip C, Chae HZ, Park EM (2006). Prx1 suppresses radiation-induced c-Jun NH2-terminal kinase signaling in lung cancer cells through interaction with the glutathione S-transferase Pi/c-Jun NH2-terminal kinase complex.. Cancer Res.

[34] Shen HM, Liu ZG (2006). JNK signaling pathway is a key modulator in cell death mediated by reactive oxygen and nitrogen species.. Free Radic Biol Med.

[35] Kyriakis JM, Avruch J (2001). Mammalian mitogen-activated protein kinase signal transduction pathways activated by stress and inflammation.. Physiol Rev.

[36] Kins S, Kurosinski P, Nitsch RM, Gotz J (2003). Activation of the ERK and JNK signaling pathways caused by neuron-specific inhibition of PP2A in transgenic mice.. Am J Pathol.

[37] Fu W, Sanders-Beer BE, Katz KS, Maglott DR, Pruitt KD (2009). Human immunodeficiency virus type 1, human protein interaction database at NCBI.. Nucleic Acids Res.

[38] Ptak RG, Fu W, Sanders-Beer BE, Dickerson JE, Pinney JW (2008). Cataloguing the HIV type 1 human protein interaction network.. AIDS Res Hum Retroviruses.

[39] Chow YH, Yu D, Zhang JY, Xie Y, Wei OL (2002). gp120-Independent infection of CD4(−) epithelial cells and CD4(+) T-cells by HIV-1.. J Acquir Immune Defic Syndr.

[40] Pang S, Yu D, An DS, Baldwin GC, Xie Y (2000). Human immunodeficiency virus Env-independent infection of human CD4(−) cells.. J Virol.

[41] Liu X, Zha J, Chen H, Nishitani J, Camargo P (2003). Human immunodeficiency virus type 1 infection and replication in normal human oral keratinocytes.. J Virol.

[42] Moir S, Chun TW, Fauci AS Pathogenic mechanisms of HIV disease.. Annu Rev Pathol.

[43] Challacombe SJ, Naglik JR (2006). The effects of HIV infection on oral mucosal immunity.. Adv Dent Res.

[44] Rawat P, Mitra D Cellular heat shock factor 1 positively regulates human immunodeficiency virus-1 gene expression and replication by two distinct pathways.. Nucleic Acids Res.

[45] Kocsis J, Prohaszka Z, Biro A, Fust G, Banhegyi D (2003). Elevated levels of antibodies against 70 kDa heat shock proteins in the sera of patients with HIV infection.. J Med Virol.

[46] Nicchitta CV (2003). Re-evaluating the role of heat-shock protein-peptide interactions in tumour immunity.. Nat Rev Immunol.

[47] Henderson B, Allan E, Coates AR (2006). Stress wars: the direct role of host and bacterial molecular chaperones in bacterial infection.. Infect Immun.

[48] Kebba A, Stebbing J, Rowland S, Ingram R, Agaba J (2005). Expression of the common heat-shock protein receptor CD91 is increased on monocytes of exposed yet HIV-1-seronegative subjects.. J Leukoc Biol.

[49] Holaska JM, Black BE, Rastinejad F, Paschal BM (2002). Ca2+-dependent nuclear export mediated by calreticulin.. Mol Cell Biol.

[50] Hirata F, Hirata A (1990). Biology of phospholipase inhibitory proteins.. Adv Exp Med Biol.

[51] Miele L (2003). New weapons against inflammation: dual inhibitors of phospholipase A2 and transglutaminase.. J Clin Invest.

[52] Sundaram M, Saghayam S, Priya B, Venkatesh KK, Balakrishnan P (2008). Changes in antioxidant profile among HIV-infected individuals on generic highly active antiretroviral therapy in southern India.. Int J Infect Dis.

[53] Muller F, Svardal AM, Nordoy I, Berge RK, Aukrust P (2000). Virological and immunological effects of antioxidant treatment in patients with HIV infection.. Eur J Clin Invest.

[54] Gess B, Hofbauer KH, Wenger RH, Lohaus C, Meyer HE (2003). The cellular oxygen tension regulates expression of the endoplasmic oxidoreductase ERO1-Lalpha.. Eur J Biochem.

[55] May D, Itin A, Gal O, Kalinski H, Feinstein E (2005). Ero1-L alpha plays a key role in a HIF-1-mediated pathway to improve disulfide bond formation and VEGF secretion under hypoxia: implication for cancer.. Oncogene.

[56] Li X, Feng J, Sun R Oxidative stress induces reactivation of Kaposi's sarcoma-associated herpesvirus and death of primary effusion lymphoma cells.. J Virol.

[57] Perluigi M, Giorgi A, Blarzino C, De Marco F, Foppoli C (2009). Proteomics analysis of protein expression and specific protein oxidation in human papillomavirus transformed keratinocytes upon UVB irradiation.. J Cell Mol Med.

[58] Wang T, Arifoglu P, Ronai Z, Tew KD (2001). Glutathione S-transferase P1-1 (GSTP1-1) inhibits c-Jun N-terminal kinase (JNK1) signaling through interaction with the C terminus.. J Biol Chem.

[59] Tanaka T, Zhong J, Iqbal K, Trenkner E, Grundke-Iqbal I (1998). The regulation of phosphorylation of tau in SY5Y neuroblastoma cells: the role of protein phosphatases.. FEBS Lett.

[60] Chung H, Brautigan DL (1999). Protein phosphatase 2A suppresses MAP kinase signalling and ectopic protein expression.. Cell Signal.

[61] Zai A, Rudd MA, Scribner AW, Loscalzo J (1999). Cell-surface protein disulfide isomerase catalyzes transnitrosation and regulates intracellular transfer of nitric oxide.. J Clin Invest.

[62] Konig J, Baier M, Horling F, Kahmann U, Harris G (2002). The plant-specific function of 2-Cys peroxiredoxin-mediated detoxification of peroxides in the redox-hierarchy of photosynthetic electron flux.. Proc Natl Acad Sci U S A.

[63] Honer B, Shoeman RL, Traub P (1991). Human immunodeficiency virus type 1 protease microinjected into cultured human skin fibroblasts cleaves vimentin and affects cytoskeletal and nuclear architecture.. J Cell Sci.

[64] Joshi R, Tawfik A, Edeh N, McCloud V, Looney S (2010). Dentin sialophosphoprotein (DSPP) gene-silencing inhibits key tumorigenic activities in human oral cancer cell line, OSC2.. PLoS One.

[65] Ji JH, Jang YJ (2009). Cellular effects of genotoxic stress and gene silencing of the checkpoint kinases in human oral cells.. J Oral Pathol Med.

[66] Krisanaprakornkit S, Kimball JR, Weinberg A, Darveau RP, Bainbridge BW (2000). Inducible expression of human beta-defensin 2 by Fusobacterium nucleatum in oral epithelial cells: multiple signaling pathways and role of commensal bacteria in innate immunity and the epithelial barrier.. Infect Immun.

[67] Krisanaprakornkit S, Weinberg A, Perez CN, Dale BA (1998). Expression of the peptide antibiotic human beta-defensin 1 in cultured gingival epithelial cells and gingival tissue.. Infect Immun.

[68] Shevchenko A, Wilm M, Vorm O, Mann M (1996). Mass spectrometric sequencing of proteins silver-stained polyacrylamide gels.. Anal Chem.

