# Supplementary figures and images for: Proteomic Signatures of Human Oral Epithelial Cells in HIV-Infected Subjects
Source: PLoS One. 2011 Nov 16;6(11):e27816. doi: 10.1371/journal.pone.0027816 (PMC3218055; doi:10.1371/journal.pone.0027816)

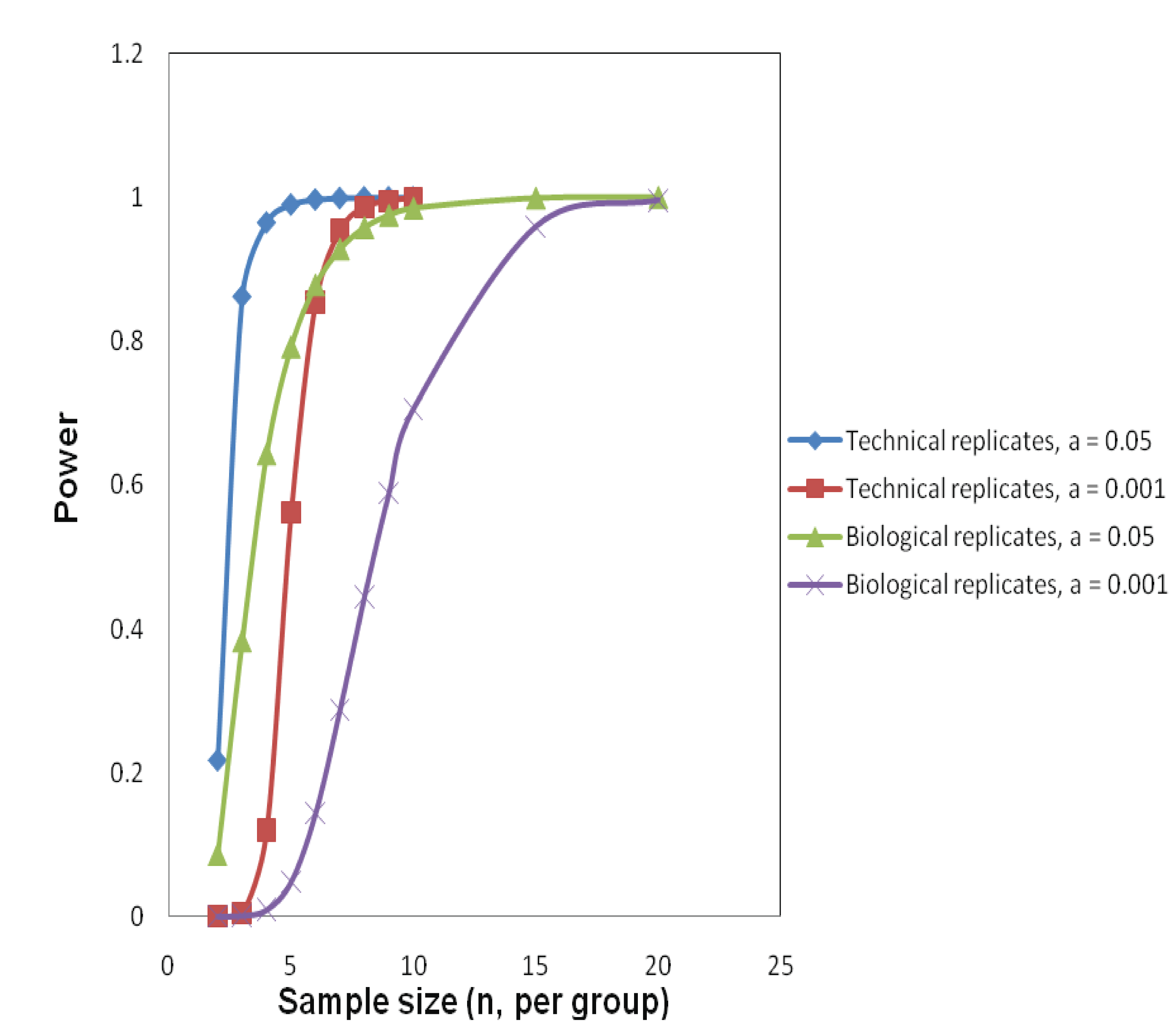

Supplement: Figure S1 — The relationship between power and sample size at α = 0.05 or 0.001 for 50% change in protein expression. Two datasets from technical replicates and biological replicates encompassing technical noise only and both technical and biological noises respectively were used to estimate the upper quartile variances and to compute power at various sample size. (TIF) [file pone.0027816.s001.tif]
