# Supplementary material for: Proteomic Signatures of Human Oral Epithelial Cells in HIV-Infected Subjects
Source: PLoS One. 2011 Nov 16;6(11):e27816. doi: 10.1371/journal.pone.0027816 (PMC3218055; doi:10.1371/journal.pone.0027816)
Supplement: Table S1 — Clinical data of the HIV-infected subjects used for 2-D DIGE and/or validation experiments. The samples from the first 4 subjects, who are HIV-infected on HAART, were pooled for the 2-D DIGE analysis. The subject samples 5–11 were used for the verification analysis. Samples from HIV-infected on HAART subjects 5, 6,and 7 were presented in the Western blot analyses of Figure 4. Samples from subjects 8, 9, 10, and 11 were used for the Western blot analyses presented in Figure 5. Patients 8 and 11 are HAART naïve while 9 and 10 are on HAART. 1The mean viral load for subjects 5–11 does not include subjects 8 and 11, who were HAART naïve. (DOC) [file pone.0027816.s002.doc]

**Table S1**: Clinical data of the HIV-infected subjects used for 2-D DIGE and/or validation experiments. The samples from the first 4 subjects, who are HIV-infected on HAART, were pooled for the 2-D DIGE analysis. The subject samples 5-11 were used for the verification analysis. Samples from HIV-infected on HAART subjects 5, 6,and 7 were presented in the Western blot analyses of Figure 4. Samples from subjects 8, 9, 10, and 11 were used for the Western blot analyses presented in Figure 5. Patients 8 and 11 are HAART naïve while 9 and 10 are on HAART. ^1^The mean viral load for subjects 5-11 does not include subjects 8 and 11, who were HAART naïve.

| Patient | Age | Years positive | CD4 cell (cells/mL) during tissue collection | Viral Load (virus/mL) | Nadir CD4 (cells/mL) |
| --- | --- | --- | --- | --- | --- |
| 1 | 43 | 3 | 700 | 50 | 6 |
| 2 | 43 | 1 | 120 | 65000 | 8 |
| 3 | 50 | 6 | 650 | 8870 | 447 |
| 4 | 47 | 13 | 600 | 60 | 140 |
| 5 | 50 | 23 | 800 | 80 | 210 |
| 6 | 48 | 11 | 250 | 12000 | 137 |
| 7 | 43 | 13 | 800 | 100 | 312 |
| 8 | 24 | 5 | 150 | 202879 | 126 |
| 9 | 51 | 20 | 770 | 48 | 210 |
| 10 | 53 | 10 | 1730 | 48 | 63 |
| 11 | 58 | 8 | 225 | 20192 | 168 |
| Mean for subjects 1-4 |  |  | 517 ± 268 | 18495 | 150 ± 148 |
| Mean for subjects 8-11^1^ |  |  | 770 ± 550 | 2455^1^ | 168 ± 79 |
