# Supplementary material for: Proteomic Signatures of Human Oral Epithelial Cells in HIV-Infected Subjects
Source: PLoS One. 2011 Nov 16;6(11):e27816. doi: 10.1371/journal.pone.0027816 (PMC3218055; doi:10.1371/journal.pone.0027816)
Supplement: Table S2 — Differential proteome profile of oral epithelial form HIV-infected on HAART subjects. (DOC) [file pone.0027816.s003.doc]

**Table S2:** Differential proteome profile of oral epithelial form HIV-infected on HAART subjects

| Gene name | Protein identity | Fold Change | | Probability T-test |
| --- | --- | --- | --- | --- |
| *Actl6a* | actin-like 6A isoform 1 | -1.53 | 2.2E-05 | |
| *Ahcy* | S-adenosylhomocysteine hydrolase | -2.7 | 8.4E-05 | |
| *Alb* | serum albumin precursor | -3.15 | 6.2E-09 | |
| *Anxa3* | annexin III | -1.63 | 3.6E-03 | |
| *Anxa4* | annexin IV | -2.5 | 2.2E-07 | |
| *Apb* | aminopeptidase B | -2.08 | 4.1E-07 | |
| *Asmtl* | **N-acetylserotonin O-methyltransferase-like protein** | -2.26 | 3.4E-04 | |
| *Atp5l* | ATP synthase, H+ transporting, mitochondrial F0 complex, subunit G | -1.62 | 1.1E-03 | |
| *Bcsg1* | BCSG1 protein | -1.5 | 1.9E-03 | |
| *C14orf166* | homeobox prox 1 | -1.5 | 2.0E-04 | |
| *Calr* | calreticulin precursor | -2.93 | 4.6E-09 | |
| *Crabp2* | cellular retinoic acid binding protein 2 | 1.93 | 4.5E-06 | |
| *Crnn* | cornulin | 1.72 | 2.3E-06 | |
| *Cryab* | Crystallin, alpha B | -1.66 | 9.5E-06 | |
| *Csta* | cystatin A | -2.18 | 6.4E-06 | |
| *Cstb* | cystatin B (stefin B) | -1.65 | 1.3E-04 | |
| *Dnajc7* | DnaJ (Hsp40) homolog, subfamily C, member 7 | -2.03 | 2.5E-05 | |
|  |  |  |  | |
| *Ero1l* | **ERO1-like protein alpha** | -1.97 | 1.3E-04 | |
| *Ethe1* | ethylmalonic encephalopathy 1 | 1.52 | 2.5E-04 | |
| *Exoc7* | exocyst complex component 7 | -2.03 | 9.8E-04 | |
| *Fkbp4* | FK506-binding protein 4 | -1.99 | 3.8E-03 | |
| *Fth1* | ferritin, heavy polypeptide 1 | -1.55 | 1.9E-03 | |
| *Ftl* | ferritin, light polypeptide | -2.29 | 3.5E-05 | |
| *Gfpt1* | glucosamine-fructose-6-phosphate aminotransferase | -2.88 | 3.8E-09 | |
| *Gpn1* | GPN-loop GTPase 1 | -1.72 | 1.1E-05 | |
| *Gsn* | gelsolin isoform a precursor | -1.6 | 1.4E-05 | |
| *Gstp1* | glutathione S-transferase | -1.99 | 4.7E-08 | |
| *Habp1/* C1qbp | complement component 1, q subcomponent binding protein | -1.67 | 4.2E-04 | |
| *Hist1h4e* | histone , H4 | -3.55 | 6.20E-06 | |
| *Hpxel* | peroxisomal enoyl-CoA hydratase-like protein | 1.74 | 7.5E-03 | |
| *Hspb1* | heat shock 27kDa protein 1 | -1.52 | 2.5E-04 | |
| *Hspd1* | heat shock protein 60 | -2.1 | 4.80E-05 | |
| *Il1rn* | interleukin 1 receptor antagonist isoform 1 | -2.55 | 7.5E-07 | |
| *Ivl* | Involucrin | -3.0 | 2.9E-10 | |
| *Lgals3bp* | galectin-3-binding protein | -2.22 | 1.2E-05 | |
| *Lima1* | LIM domain and actin binding 1 | -2.7 | 7.9E-07 | |
| *Mmab* | cob(I)alamin adenosyltransferase precursor | -1.5 | 3.10E-07 | |
| *Mtpn* | myotrophin | -1.81 | 3.1E-06 | |
| *Ndrg1* | N-myc downstream-regulated gene 1 protein | 2.1 | 3.4E-07 | |
| *Nit2* | nitrilase family, member 2 | -1.54 | 2.0E-06 | |
| *Pdia3* | protein disulfide-isomerase A3 precursor | 1.57 | 7.5E-07 | |
| *Pgm2* | phosphoglucomutase 2 variant | -2.48 | 5.9E-05 | |
| *Phb* | prohibitin | -1.53 | 2.2E-05 | |
| *Ppa2* | inorganic pyrophosphatase 2 | 4.42 | 3.6E-05 | |
| *Ppp2r1a* | protein phosphatase 2, regulatory subunit A, alpha isoform | -2.75 | 1.4E-06 | |
| *Prdx1* | peroxiredoxin 1 | -1.53 | 2.5E-03 | |
| *Prmt5* | protein arginine methyltransferase 5 isoform b | -1.87 | 7.3E-04 | |
| *Psmb6* | proteasome beta 6 subunit | -2.64 | 3.7E-07 | |
| *Rnh1* | ribonuclease/angiogenin inhibitor | -1.65 | 5.6E-04 | |
| *S100a16* | S100 calcium binding protein A16 | -3.36 | 3.6E-07 | |
| *Serpinb5* | proteinase inhibitor, clade B (ovalbumin), member 5 | -2.72 | 3.4E-06 | |
| *Slc25a24* | calcium-binding mitochondrial carrier protein SCaMc-1 isoform 1 | -2.3 | 3.5E-06 | |
| *Tkt* | transketolase | -2.66 | 6.9E-07 | |
| *Tom1* | target of myb1 | -1.96 | 6.5E-03 | |
| *Trap1/Hsp90L* | TNF receptor-associated protein 1/mitochondrial Hsp90 | -1.8 | 1.8E-06 | |
| *Tubb4* | tubulin, beta 4 | -2.8 | 2.7E-06 | |
| *Tunp* | transformation upregulated nuclear protein or HNRNPK heterogeneous nuclear ribonucleoprotein K | -3.17 | 2.8E-07 | |
| *Vil2/ Ezr* | Villin 2 **(Ezrin)** | -2.51 | 4.1E-04 | |
| *Vdac2* | voltage-dependent anion channel 2, isoform CRA_b | -1.6 | 6.5E-04 | |
| *Vim* | Vimentin | 2.42 | 4.0E-04 | |
|  |  |  |  | |
| *Xrcc5* | ATP-dependent DNA helicase II | -1.73 | 3.3E-04 | |
