# Supplementary material for: Proteomic Signatures of Human Oral Epithelial Cells in HIV-Infected Subjects
Source: PLoS One. 2011 Nov 16;6(11):e27816. doi: 10.1371/journal.pone.0027816 (PMC3218055; doi:10.1371/journal.pone.0027816)
Supplement: Table S3 — Database Search Results of spectra acquired on a Fourier Transform LTQ Mass Spectrometry. The tandem mass spectra were annotated and generated peak list files (.DTA), by running SEQUEST extract_msn algorithm in Bioworks version 3.2 (Thermo Electron, Bremen, Germany). The resulting peptide mass lists were then used to interrogated sequences present in an indexed human subset database (137,607 sequences), that was created from NCBInr 3,893,302 sequences (release 07/04/06) and stored locally, by running SEQUEST SEARCH algorithm of Bioworks software version 3.2. SEQUEST searching were performed with maximum peptide and fragment ion mass tolerance of 2.5 and 1.0 Da respectively, and with partial methionine oxidation (M)and complete carbamidomethylation of cysteine (C), and 2 missed cleavage sites were also allowed in the search parameter. For each protein identification, a minimum of two peptides with a significant peptide expectation (P<0.001), peptide Xcorr 1.9, 2.7, and 3.5 for the charge states and +1, +2, and +3 respectively, minimum Delta CN (Delta correlation) of 0.1. (DOC) [file pone.0027816.s004.doc]

**Table S3**. Database Search Results of spectra acquired on a Fourier Transform LTQ Mass Spectrometry. The tandem mass spectra were annotated and generated peak list files (.DTA), by running SEQUEST extract_msn algorithm in Bioworks version 3.2 (Thermo Electron, Bremen, Germany). The resulting peptide mass lists were then used to interrogated sequences present in an indexed human subset database (137,607 sequences), that was created from NCBInr 3,893,302 sequences (release 07/04/06) and stored locally, by running SEQUEST SEARCH algorithm of Bioworks software version 3.2. SEQUEST searching were performed with maximum peptide and fragment ion mass tolerance of 2.5 and 1.0 Da respectively, and with partial methionine oxidation (M)and complete carbamidomethylation of cysteine (C), and 2 missed cleavage sites were also allowed in the search parameter. For each protein identification, a minimum of two peptides with a significant peptide expectation (P<0.001), peptide Xcorr 1.9, 2.7, and 3.5 for the charge states and +1, +2, and +3 respectively, minimum Delta CN (Delta correlation) of 0.1.

| Pos. | Gene name |  | LC-MS/MS | | | | | | | |
| --- | --- | --- | --- | --- | --- | --- | --- | --- | --- | --- |
|  |  | Accession number | P (Pro.)  P (pep.) | % seq. cove. | Score  Xc, z | ΔCn | Measured mass (M + H) | ΔM | Peptide Sequence | Modification |
| 1 | *Actl6a* | gi4757718 | 1.83E-10 | 22.60 | 90.32 |  |  |  |  |  |
|  |  |  | 5.07E-06 |  | 3.65, 2 | 0.46 | 1130.61646 | 0.00298 | K.LIANNTTVER.R |  |
|  |  |  | 4.42E-06 |  | 2.82, 2 | 0.37 | 1596.70574 | 0.01415 | R.DDGSTLM#EIDGDKGK.Q | Oxidation (M) |
|  |  |  | 1.46E-07 |  | 2.70, 2 | 0.33 | 1580.71082 | 0.01372 | R.DDGSTLMEIDGDKGK.Q |  |
|  |  |  | 6.67E-10 |  | 3.98, 3 | 0.54 | 2040.98074 | 0.01789 | K.SEASLHPVLM#SEAPWNTR.A | Oxidation (M) |
|  |  |  | 2.30E-07 |  | 3.95, 2 | 0.56 | 2040.98074 | 0.02004 | K.SEASLHPVLM#SEAPWNTR.A | Oxidation (M) |
|  |  |  | 1.45E-09 |  | 3.90, 2 | 0.61 | 1511.72863 | -0.02143 | K.SPLAGDFITM#QC*R.E | Carbamidomethylation (C) |
|  |  |  | 9.27E-09 |  | 3.72, 2 | 0.63 | 1568.77039 | 0.00725 | K.QGGPTYYIDTNALR.V |  |
|  |  |  | 1.55E-07 |  | 3.03, 2 | 0.42 | 1556.86829 | 0.01299 | R.LKIPEGLFDPSNVK.G |  |
|  |  |  | 3.58E-07 |  | 3.83, 2 | 0.52 | 1449.74061 | 0.01249 | K.VDFPTAIGM#VVER.D | Oxidation (M) |
|  |  |  | 1.83E-10 |  | 4.18, 2 | 0.54 | 1433.74573 | 0.01189 | K.VDFPTAIGMVVER.D |  |
| 2 | *Ahcy* | gi30584089 | 1.82E-10 | 39.30 | 228.29 |  |  |  |  |  |
|  |  |  | 3.38E-04 |  | 3.20, 2 | 0.25 | 1061.57715 | 0.00896 | K.RATDVMIAGK.V |  |
|  |  |  | 1.39E-05 |  | 2.79, 2 | 0.30 | 921.47098 | 0.00722 | R.ATDVM#IAGK.V | Oxidation (M) |
|  |  |  | 2.91E-05 |  | 2.77, 2 | 0.26 | 1068.61597 | 0.00994 | K.VNIKPQVDR.Y |  |
|  |  |  | 3.87E-05 |  | 2.92, 2 | 0.29 | 905.47607 | 0.00762 | R.ATDVMIAGK.V |  |
|  |  |  | 1.21E-08 |  | 5.45, 3 | 0.59 | 1380.74817 | 0.01316 | K.KLDEAVAEAHLGK.L |  |
|  |  |  | 2.64E-09 |  | 3.74, 2 | 0.35 | 138.74817 | 0.01531 | K.KLDEAVAEAHLGK.L |  |
|  |  |  | 2.71E-08 |  | 4.54, 2 | 0.46 | 1252.65320 | 0.01494 | K.LDEAVAEAHLGK.L |  |
|  |  |  | 3.93E-05 |  | 3.57, 3 | 0.53 | 1252.65320 | 0.00919 | K.LDEAVAEAHLGK.L |  |
|  |  |  | 1.91E-06 |  | 3.59, 2 | 0.47 | 1259.61427 | -0.01913 | K.SKFDNLYGC*R.E | Carbamidomethylation (C) |
|  |  |  | 3.88E-07 |  | 4.79, 3 | 0.46 | 1719.84039 | 0.01286 | R.KALDIAENEM#PGLM#R.M | Oxidation (M) |
|  |  |  | 4.41E-09 |  | 3.48, 2 | 0.55 | 1719.84039 | 0.02392 | R.KALDIAENEM#PGLM#R.M | Oxidation (M) |
|  |  |  | 9.27E-08 |  | 4.36, 2 | 0.56 | 1648.81775 | 0.01409 | R.GISEETTTGVHNLYK.M |  |
|  |  |  | 2.96E-05 |  | 3.68, 2 | 0.49 | 1040.65747 | 0.00701 | R.RIILLAEGR.L |  |
|  |  |  | 5.73E-07 |  | 3.14, 1 | 0.41 | 1134.61536 | 0.02063 | K.VAVVAGYGDVGK.G |  |
|  |  |  | 7.28E-09 |  | 4.60, 2 | 0.49 | 1134.61536 | 0.01079 | K.VAVVAGYGDVGK.G |  |
|  |  |  | 1.71E-08 |  | 2.82, 2 | 0.48 | 1591.74543 | 0.01927 | K.ALDIAENEM#PGLM#R.M | Oxidation (M) |
|  |  |  | 6.04E-08 |  | 3.65, 3 | 0.43 | 1591.74543 | 0.01364 | K.ALDIAENEM#PGLM#R.M | Oxidation (M) |
|  |  |  | 2.34E-06 |  | 3.30, 2 | 0.40 | 1156.65137 | 0.01286 | K.YPVGVHFLPK.K |  |
|  |  |  | 5.29E-05 |  | 2.78, 2 | 0.40 | 884.55640 | 0.00721 | R.IILLAEGR.L |  |
|  |  |  | 1.86E-04 |  | 2.31, 2 | 0.58 | 1575.75053 | 0.02296 | K.ALDIAENEMPGLM#R.M | Oxidation (M) |
|  |  |  | 1.53E-09 |  | 3.48, 2 | 0.23 | 1575.75053 | 0.02162 | K.ALDIAENEM#PGLMR.M | Oxidation (M) |
|  |  |  | 1.82E-10 |  | 3.77, 2 | 0.53 | 1256.68445 | 0.01726 | K.VPAINVNDSVTK.S |  |
|  |  |  | 6.29E-06 |  | 2.90, 2 | 0.40 | 1056.62000 | 0.00994 | K.YPQLLPGIR.G |  |
|  |  |  | 2.10E-04 |  | 1.81, 1 | 0.31 | 1056.62000 | 0.02258 | K.YPQLLPGIR.G |  |
|  |  |  | 2.81E-08 |  | 3.63, 2 | 0.58 | 1559.75562 | 0.02532 | K.ALDIAENEMPGLMR.M |  |
|  |  |  | 1.44E-06 |  | 3.64, 2 | 0.34 | 1128.61597 | 0.01201 | K.VADIGLAAWGR.K |  |
| 3 | *Alb* | **P02768** | 1.47E-09 | 12.80 | 60.18 |  |  |  |  |  |
|  |  |  | 3.08E-04 |  | 1.86, 1 | 0.41 | 1138.55903 | -0.03522 | K.C*C*TESLVNR.R | Carbamidomethylation (C) |
|  |  |  | 6.72E-04 |  | 2.81, 2 | 0.29 | 927.49347 | 0.00359 | K.YLYEIAR.R |  |
|  |  |  | 3.34E-04 |  | 2.75, 2 | 0.33 | 1138.55903 | -0.04482 | K.C*C*TESLVNR.R | Carbamidomethylation (C) |
|  |  |  | 8.00E-07 |  | 2.83, 2 | 0.60 | 1657.78318 | -0.00958 | K.QNC*ELFEQLGEYK.F |  |
|  |  |  | 1.47E-09 |  | 3.64, 2 | 0.53 | 2260.05321 | 0.00104 | K.EFNAETFTFHADIC*TLSEK.E | Carbamidomethylation (C) |
|  |  |  | 1.29E-05 |  | 3.59, 3 | 0.36 | 2045.09534 | 0.02427 | K.VFDEFKPLVEEPQNLIK.Q |  |
|  |  |  | 1.78E-07 |  | 2.80, 2 | 0.45 | 1639.78248 | 0.01298 | K.DVFLGM#FLYEYAR.R | Oxidation (M) |
| 4 | *Anxa3* | **P12429** | 6.66E-15 | 59.10 | 200.27 |  |  |  |  |  |
|  |  |  | 4.47E-09 |  | 5.28, 3 | 0.61 | 1539.81250 | 0.01419 | R.RDESLKVDEHLAK.Q |  |
|  |  |  | 1.01E-09 |  | 4.32, 2 | 0.54 | 1539.81250 | 0.00945 | R.RDESLKVDEHLAK.Q |  |
|  |  |  | 5.46E-06 |  | 2.42, 1 | 0.11 | 929.43634 | 0.01385 | K.EYQAAYGK.E |  |
|  |  |  | 1.55E-05 |  | 1.98, 1 | 0.26 | 978.52545 | 0.00653 | K.QDAQILYK.A |  |
|  |  |  | 4.34E-09 |  | 2.54, 2 | 0.42 | 1429.74744 | 0.01323 | K.KHYGYSLYSAIK.S |  |
|  |  |  | 7.69E-04 |  | 5.29, 3 | 0.50 | 1429.74744 | 0.00962 | K.KHYGYSLYSAIK.S |  |
|  |  |  | 3.31E-06 |  | 3.62, 2 | 0.43 | 1057.63647 | 0.00725 | R.KALLTLADGR.R |  |
|  |  |  | 2.05E-08 |  | 3.56, 2 | 0.45 | 1383.71143 | 0.01091 | R.DESLKVDEHLAK.Q |  |
|  |  |  | 1.87E-12 |  | 5.20, 3 | 0.53 | 1841.88757 | 0.01499 | K.KSLGDDISSETSGDFRK.A |  |
|  |  |  | 4.35E-05 |  | 4.32, 2 | 0.53 | 1841.88757 | 0.02654 | K.KSLGDDISSETSGDFRK.A |  |
|  |  |  | 1.56E-07 |  | 3.17, 1 | 0.41 | 1301.65247 | 0.02380 | K.HYGYSLYSAIK.S |  |
|  |  |  | 1.29E-08 |  | 3.49, 2 | 0.53 | 1301.65247 | 0.00224 | K.HYGYSLYSAIK.S |  |
|  |  |  | 1.56E-10 |  | 3.80, 2 | 0.57 | 1713.79260 | 0.01872 | K.SLGDDISSETSGDFRK.A |  |
|  |  |  | 3.73E-06 |  | 2.78, 2 | 0.44 | 929.54144 | 0.00383 | K.ALLTLADGR.R |  |
|  |  |  | 2.03E-07 |  | 2.45, 1 | 0.39 | 1222.60620 | 0.01306 | K.GIGTDEFTLNR.I |  |
|  |  |  | 4.56E-09 |  | 1.92, 1 | 0.37 | 1585.69763 | 0.03833 | K.SLGDDISSETSGDFR.K |  |
|  |  |  | 3.94E-05 |  | 2.43, 1 | 0.26 | 1350.65759 | 0.02063 | K.DISQAYYTVYK.K |  |
|  |  |  | 4.97E-05 |  | 2.02, 1 | 0.33 | 1441.70569 | 0.02039 | K.SDTSGDYEITLLK.I |  |
|  |  |  | 3.51E-08 |  | 2.46, 1 | 0.46 | 1781.82288 | 0.03271 | R.DYPDFSPSVDAEAIQK.A |  |
|  |  |  | 3.66E-05 |  | 3.52, 2 | 0.35 | 1073.58374 | 0.00237 | R.SEIDLLDIR.T |  |
|  |  |  | 1.13E-04 |  | 2.54, 1 | 0.35 | 1073.58374 | 0.00513 | R.SEIDLLDIR.T |  |
|  |  |  | 1.31E-11 |  | 4.18, 2 | 0.49 | 1882.93091 | -0.00813 | R.WGTDEDKFTEILC*LR.S |  |
|  |  |  | 4.94E-05 |  | 3.09, 2 | 0.25 | 1091.61289 | 0.01362 | K.M#LISILTER.S |  |
|  |  |  | 1.06E-04 |  | 3.08, 1 | 0.40 | 1673.87048 | 0.04333 | K.GAGTNEDALIEILTTR.T |  |
|  |  |  | 1.17E-05 |  | 4.16, 3 | 0.49 | 2551.30127 | 0.06357 | K.GDLSGHFEHLMVALVTPPAVFDAK.Q |  |
|  |  |  | 1.94E-09 |  | 4.11, 2 | 0.42 | 1673.87048 | 0.02703 | K.GAGTNEDALIEILTTR.T |  |
|  |  |  | 7.18E-13 |  | 3.79, 2 | 0.61 | 1585.69763 | 0.01775 | K.SLGDDISSETSGDFR.K |  |
|  |  |  | 5.87E-07 |  | 3.68, 3 | 0.52 | 1882.93091 | -0.01218 | R.WGTDEDKFTEILC*LR.S |  |
|  |  |  | 5.58E-06 |  | 3.42, 3 | 0.43 | 1673.87048 | 0.01322 | K.GAGTNEDALIEILTTR.T |  |
|  |  |  | 2.69E-07 |  | 3.16, 2 | 0.50 | 1350.65759 | 0.00847 | K.DISQAYYTVYK.K |  |
|  |  |  | 4.49E-08 |  | 3.20, 2 | 0.42 | 1222.60620 | 0.00615 | K.GIGTDEFTLNR.I |  |
|  |  |  | 6.66E-15 |  | 2.88, 2 | 0.45 | 1781.82288 | 0.01531 | R.DYPDFSPSVDAEAIQK.A |  |
|  |  |  | 1.98E-07 |  | 2.88, 2 | 0.46 | 1441.70569 | 0.01548 | K.SDTSGDYEITLLK.I |  |
| 5 | *Anxa4* | gi4502105 | 3.98E-10 |  | 80.23 |  |  |  |  |  |
|  |  |  | 3.65E-08 |  | 3.37, 2 | 0.43 | 1371.66516 | 0.02764 | R.ISQTYQQQYGR.S |  |
|  |  |  | 3.98E-10 |  | 3.09. 2 | 0.55 | 1597.72748 | 0.02391 | K.AASGFNAM#EDAQTLR.K | Oxidation (M) |
|  |  |  | 3.75E-06 |  | 3.26, 2 | 0.35 | 1174.60620 | 0.01457 | K.GLGTDDNTLIR.V |  |
|  |  |  | 3.10E-08 |  | 3.35, 2 | 0.47 | 1581.73254 | 0.01836 | K.AASGFNAMEDAQTLR.K |  |
|  |  |  | 1.95E-07 |  | 1.81, 1 | 0.27 | 1060.56486 | -0.00443 | K.VLLVLC*GGDD | Carbamidomethylation (C) |
|  |  |  | 6.44E-07 |  | 3.53, 2 | 0.57 | 1661.84685 | -0.00610 | K.GAGTDEGC*LIEILASR.T | Carbamidomethylation (C) |
|  |  |  | 7.08E-10 |  | 4.01, 2 | 0.57 | 1692.88025 | 0.01872 | K.GLGTDEDAIISVLAYR.N |  |
|  |  |  | 1.21E-09 |  | 3.05, 2 | 0.57 | 1666.85339 | 0.02373 | K.SETSGSFEDALLAIVK.C |  |
| 6 | *Apb* | **Q9H4A4** | 1.93E-08 | 26.80 | 150.25 |  |  |  |  |  |
|  |  |  | 7.75E-04 |  | 4.11, 3 | 0.35 | 1641.75370 | 1.10205 | R.QHM#DITGEENPLNK.L | Oxidation (M) |
|  |  |  | 1.93E-08 |  | 5.01, 3 | 0.57 | 1925.99402 | 1.19284 | R.RPLHSAQAVDVASASNFR.A |  |
|  |  |  | 2.02E-07 |  | 2.84, 2 | 0.49 | 1199.58032 | 1.00859 | R.AEFGPPGPGAGSR.G |  |
|  |  |  | 5.29E-07 |  | 4.27, 3 | 0.41 | 1625.75879 | 0.98661 | R.QHMDITGEENPLNK.L |  |
|  |  |  | 1.31E-06 |  | 2.85, 2 | 0.48 | 1293.64331 | 0.84563 | K.LGDTYPSISNAR.N |  |
|  |  |  | 1.75E-05 |  | 4.27, 2 | 0.46 | 1325.75879 | -0.40864 | R.QHMDITGEENPLNK.L |  |
|  |  |  | 2.64E-07 |  | 4.01, 3 | 0.47 | 2309.08203 | 1.13657 | R.VKIEPGVDPDDTYNETPYEK.G |  |
|  |  |  | 6.17E-08 |  | 3.28, 2 | 0.64 | 2081.91870 | 0.25078 | K.IEPGVDPDDTYNETPYEK.G |  |
|  |  |  | 1.03E-05 |  | 4.07, 3 | 0.48 | 2313.12534 | 0.63855 | K.YTLPLYHAM#M#GGSEVAQTLAK.E | Oxidation (M) |
|  |  |  | 3.55E-04 |  | 3.64, 2 | 0.51 | 1101.62622 | 0.42656 | R.GLSGTAVLDLR.C |  |
|  |  |  | 3.05E-06 |  | 3.18, 2 | 0.50 | 1184.66736 | 0.01702 | K.AIEAVAISPWK.T |  |
|  |  |  | 1.73E-06 |  | 2.81, 2 | 0.37 | 1094.57813 | 0.64604 | K.LFGPYVWGR.Y |  |
|  |  |  | 1.21E-06 |  | 3.10, 2 | 0.54 | 1289.67761 | -0.23115 | K.TYQLVYFLDK.I |  |
| 7 | *Asmtl* | **O95671** | 2.23E-07 | 10.90 | 40.24 |  |  |  |  |  |
|  |  |  | 5.26E-06 |  | 2.91, 2 | 0.53 | 1131.52051 | 0.40862 | R.VSEFYEETK.V |  |
|  |  |  | 7.35E-04 |  | 3.30, 2 | 0.44 | 1402.75769 | 0.39372 | K.VFDLLKDEAPQK.A |  |
|  |  |  | 2.23E-07 |  | 3.80, 2 | 0.34 | 1694.84514 | 0.92090 | R.ALM#QSLNM#LVQTEGK.E | Oxidation (M) |
|  |  |  | 5.60E-04 |  | 4.70, 3 | 0.45 | 2730.21265 | 1.40195 | K.HDSIPAADTFEDLSDVEGGGSEPTQR.D |  |
| 8 | *Atp5l* | gi47682994 | 1.96E-07 | 47.60 | 60.21 |  |  |  |  |  |
|  |  |  | 7.19E-05 |  | 2.74, 2 | 0.50 | 1279.70044 | 0.87969 | K.KIVNSAQTGSFK.Q |  |
|  |  |  | 2.19E-07 |  | 3.30, 2 | 0.59 | 1151.60547 | 1.40727 | K.IVNSAQTGSFK.Q |  |
|  |  |  | 4.18E-06 |  | 3.52, 3 | 0.50 | 1587.88538 | 1.14469 | K.TPALVNAAVTYSKPR.L |  |
|  |  |  | 4.88E-07 |  | 3.16, 2 | 0.54 | 1587.88538 | 0.49755 | K.TPALVNAAVTYSKPR.L |  |
|  |  |  | 1.96E-07 |  | 4.05, 2 | 0.59 | 1334.73145 | 0.52886 | K.TPALVNAAVTYSK.P |  |
|  |  |  | 7.48E-05 |  | 2.84, 2 | 0.46 | 1417.80493 | 0.90245 | K.VELVPPTPAEIPR.A |  |
|  |  |  | 2.37E-05 |  | 2.76, 2 | 0.49 | 1162.59314 | 0.10954 | R.LATFWYYAK.V |  |
| 9 | *Bcsg1* | gi2281474 | 1.93E-11 | 48.80 | 70.26 |  |  |  |  |  |
|  |  |  | 1.17E-09 |  | 4.43, 3 | 0.50 | 1869.93005 | 0.02481 | R.KEDLRPSAPQQEGEASK.E |  |
|  |  |  | 5.89E-10 |  | 3.48, 2 | 0.47 | 1869.93005 | 0.04314 | R.KEDLRPSAPQQEGEASK.E |  |
|  |  |  | 1.13E-07 |  | 4.37, 2 | 0.62 | 1477.62891 | 0.01665 | K.EKEEVAEEAQSGGD |  |
|  |  |  | 2.23E-09 |  | 3.55, 2 | 0.60 | 1741.83508 | 0.03166 | K.EDLRPSAPQQEGEASK.E |  |
|  |  |  | 3.18E-06 |  | 2.80, 2 | 0.58 | 1220.49133 | 0.01299 | K.EEVAEEAQSGGD |  |
|  |  |  | 3.17E-06 |  | 4.21, 3 | 0.57 | 1618.86462 | 0.01749 | K.TKENVVQSVTSVAEK.T |  |
|  |  |  | 5.90E-10 |  | 5.10, 2 | 0.51 | 1618.86462 | 0.01665 | K.TKENVVQSVTSVAEK.T |  |
|  |  |  | 4.95E-05 |  | 3.62, 2 | 0.55 | 1389.72205 | 0.01567 | K.ENVVQSVTSVAEK.T |  |
|  |  |  | 1.93E-11 |  | 3.32, 2 | 0.51 | 1676.87048 | 0.01982 | K.TVEEAENIAVTSGVVR.K |  |
| 10 | *C14orf166* | gi47682994 | 1.33E-14 | 46.30 | 120.24 |  |  |  |  |  |
|  |  |  | 4.00E-07 |  | 2.84, 2 | 0.48 | 1178.60522 | 0.01775 | K.YKDLVPDNSK.T |  |
|  |  |  | 1.28E-07 |  | 3.15, 2 | 0.54 | 1149.56086 | 0.00767 | R.HDDYLVM#LK.A | Oxidation (M) |
|  |  |  | 1.33E-14 |  | 4.31, 3 | 0.56 | 2498.17103 | 0.01980 | K.LTALDYHNPAGFNC*KDETEFR.N | Carbamidomethylation (C) |
|  |  |  | 2.60E-06 |  | 3.26, 2 | 0.37 | 1133.56592 | 0.01628 | R.HDDYLVMLK.A |  |
|  |  |  | 1.19E-11 |  | 3.98, 2 | 0.53 | 1812.91260 | 0.02019 | K.NAEPLINLDVNNPDFK.A |  |
|  |  |  | 2.87E-07 |  | 3.83, 2 | 0.41 | 1513.85189 | 0.02181 | K.AGVM#ALANLLQIQR.H | Oxidation (M) |
|  |  |  | 2.94E-06 |  | 3.28, 2 | 0.38 | 1291.66809 | 0.01238 | R.NFIVWLEDQK.I |  |
|  |  |  | 1.33E-10 |  | 4.77, 2 | 0.62 | 1664.95813 | 0.01860 | K.INEAIVAVQAIIADPK.T |  |
|  |  |  | 7.62E-07 |  | 4.15, 3 | 0.49 | 1664.95813 | 0.01474 | K.INEAIVAVQAIIADPK.T |  |
|  |  |  | 7.77E-07 |  | 3.28, 2 | 0.41 | 1497.85693 | 0.01848 | K.AGVMALANLLQIQR.H |  |
| 11 | *Calr* | gi4757900 | 6.06E-06 | 6.0 | 20.16 |  |  |  |  |  |
|  |  |  | 3.65E-05 |  | 3.28, 2 | 0.59 | 1476.68414 | -0.02613 | K.HEQNIDC*GGGYVK.L | Carbamidomethylation (C) |
|  |  |  | 6.06E-06 |  | 2.94, 2 | 0.49 | 1410.62842 | 0.00322 | K.EQFLDGDGWTSR.W |  |
|  |  |  |  |  |  |  |  |  |  |  |
|  |  |  |  |  |  |  |  |  |  |  |
| 12 | *Crabp2* | gi4503029 | 5.64E-11 | 57.28 | 100.29 |  |  |  |  |  |
|  |  |  | 5.64E-11 |  | 3.88, 3 | 0.48 | 1495.92065 | 0.01245 | R.KIAVAAASKPAVEIK.Q |  |
|  |  |  | 3.06E-10 |  | 5.17, 2 | 0.46 | 1495.92065 | 0.02165 | R.KIAVAAASKPAVEIK.Q |  |
|  |  |  | 1.58E-07 |  | 4.08, 2 | 0.36 | 1367.82569 | 0.01726 | K.IAVAAASKPAVEIK.Q |  |
|  |  |  | 5.91E-11 |  | 3.09, 2 | 0.48 | 1879.87960 | -0.00443 | K.VGEEFEEQTVDGRPC*K.S | Carbamidomethylation (C) |
|  |  |  | 9.97E-08 |  | 3.84, 2 | 0.48 | 1494.67065 | 0.01995 | K.VGEEFEEQTVDGR.P |  |
|  |  |  | 3.84E-10 |  | 3.65, 3 | 0.52 | 1879.87960 | -0.00555 | K.VGEEFEEQTVDGRPC*K.S | Carbamidomethylation (C) |
|  |  |  | 2.45E-04 |  | 2.38, 2 | 0.29 | 1128.69214 | 0.01323 | K.VLGVNVMLRK.I |  |
|  |  |  | 1.04E-10 |  | 4.62, 3 | 0.42 | 2449.33374 | 0.05362 | K.IAVAAASKPAVEIKQEGDTFYIK.T |  |
|  |  |  | 3.03E-05 |  | 3.19, 2 | 0.33 | 1016.59210 | 0.00994 | K.VLGVNVM#LR.K | Oxidation (M) |
|  |  |  | 7.14E-05 |  | 2.92, 2 | 0.16 | 1108.55212 | 0.00896 | R.SENFEELLK.V |  |
|  |  |  | 1.23E-05 |  | 3.42, 2 | 0.31 | 1000.59717 | 0.00932 | K.VLGVNVMLR.K |  |
|  |  |  | 5.88E-08 |  | 3.67, 3 | 0.51 | 2382.14684 | 0.02415 | R.ELTNDGELILTM#TADDVVC*TR.V | Oxidation (M) |
|  |  |  | 8.36E-07 |  | 3.31, 2 | 0.57 | 2382.14684 | 0.05707 | R.ELTNDGELILTM#TADDVVC*TR.V | Oxidation (M) |
| 13 | *Crnn* | gi7706635 | 9.65E-10 | 21.40 | 50.22 |  |  |  |  |  |
|  |  |  | 4.33E-08 |  | 4.15, 3 | 0.56 | 2853.33569 | 0.05960 | R.SQTSQAVTGGHTQIQAGSHTETVEQDR.S |  |
|  |  |  | 6.65E-10 |  | 3.50, 3 | 0.47 | 2500.15454 | 0.05191 | R.LDQGNLHTSVSSAQGQDAAQSEEK.R |  |
|  |  |  | 8.31E-09 |  | 2.34, 2 | 0.42 | 1785.93408 | 0.01946 | R.ISPQIQLSGQTEQTQK.A |  |
|  |  |  | 1.41E-08 |  | 4.39, 3 | 0.61 | 2550.20801 | 0.06443 | R.GQNRPGVQTQGQATGSAWVSSYDR.Q |  |
|  |  |  | 3.77E-08 |  | 2.99, 2 | 0.48 | 1753.81396 | 0.03301 | R.QPTVVGEEWVDDHSR.E |  |
| 14 | *Cryab* | gi13937813 | 1.20E-10 | 34.60 | 70.21 |  |  |  |  |  |
|  |  |  | 4.44E-04 |  | 2.64, 1 | 0.32 | 754.44574 | 0.00885 | K.PAVTAAPK.K |  |
|  |  |  | 2.32E-08 |  | 3.82, 2 | 0.56 | 1165.65759 | 0.01262 | K.VLGDVIEVHGK.H |  |
|  |  |  | 9.43E-04 |  | 2.83, 2 | 0.18 | 1374.70654 | 0.00994 | R.RPFFPFHSPSR.L |  |
|  |  |  | 9.16E-06 |  | 4.00, 3 | 0.30 | 1375.70654 | 0.01267 | R.RPFFPFHSPSR.L |  |
|  |  |  | 5.33E-05 |  | 2.58, 2 | 0.28 | 1192.63208 | 0.01250 | K.DRFSVNLDVK.H |  |
|  |  |  | 8.32E-07 |  | 2.14, 2 | 0.59 | 1512.67874 | 0.02834 | R.APSWFDTGLSEM#R.L | Oxidation (M) |
|  |  |  | 1.20E-10 |  | 3.52, 2 | 0.60 | 1496.68384 | 0.01897 | R.APSWFDTGLSEMR.L |  |
|  |  |  | 9.23E-05 |  | 2.91, 2 | 0.33 | 986.49420 | 0.00884 | K.HFSPEELK.V |  |
|  |  |  |  |  |  |  |  |  |  |  |
| 15 | *Csta* | gi4885165 | 6.75E-13 | 59.20 | 50.21 |  |  |  |  |  |
|  |  |  | 8.34E-04 |  | 2.34, 2 | 0.15 | 850.46692 | 0.00969 | K.LEAVQYK.T |  |
|  |  |  | 3.51E-07 |  | 4.13, 2 | 0.56 | 1356.71582 | 0.01677 | K.TQVVAGTNYYIK.V |  |
|  |  |  | 1.33E-07 |  | 2.76, 2 | 0.69 | 1038.47388 | 0.01335 | K.NKDDELTGF |  |
|  |  |  | 2.36E-06 |  | 3.12, 2 | 0.44 | 1339.71033 | 0.01653 | K.PATPEIQEIVDK.V |  |
|  |  |  | 6.75E-13 |  | 2.83, 2 | 0.43 | 1975.99707 | 0.03594 | K.SLPGQNEDLVLTGYQVDK.N |  |
| 16 | *Cstb* | **Q76LA1** | 1.22E-14 | 45.50 | 40.27 |  |  |  |  |  |
|  |  |  | 2.93E-08 |  | 3.68, 2 | 0.57 | 1278.66882 | 0.01531 | K.PLTLSNYQTNK.A |  |
|  |  |  | 1.22E-14 |  | 5.21, 2 | 0.53 | 2458.27246 | 0.03252 | R.VFQSLPHENKPLTLSNYQTNK.A |  |
|  |  |  | 2.98E-10 |  | 4.38, 3 | 0.63 | 1422.71240 | 0.01111 | K.VHVGDEDFVHLR.V |  |
|  |  |  | 6.13E-11 |  | 3.69, 2 | 0.63 | 1422.71240 | 0.01787 | K.VHVGDEDFVHLR.V |  |
|  |  |  | 8.79E-07 |  | 3.33, 1 | 0.21 | 1326.73520 | 0.02405 | K.SQVVAGTNYFIK.V |  |
|  |  |  | 2.36E-07 |  | 3.62, 2 | 0.46 | 1326.70520 | 0.00957 | K.SQVVAGTNYFIK.V |  |
|  |  |  | 1.25E-12 |  | 4.21, 3 | 0.48 | 2458.27246 | 0.02225 | R.VFQSLPHENKPLTLSNYQTNK.A |  |
| 17 | *Dnajc7; Hsp40* | gi40225850 | 1.14E-09 | 12.30 | 60.20 |  |  |  |  |  |
|  |  |  | 9.57E-07 |  | 2.82, 2 | 0.51 | 1173.58582 | 0.01152 | R.EALGDAQQSVR.L |  |
|  |  |  | 1.14E-09 |  | 3.90, 2 | 0.46 | 1591.79375 | -0.01575 | R.KLDDAIEDC*TNAVK.L | Carbamidomethylation (C) |
|  |  |  |  |  |  |  |  |  |  |  |
|  |  |  | 7.56E-08 |  | 3.71, 2 | 0.49 | 1371.62089 | 0.01649 | R.M#DSTNADALYVR.G | Oxidation (M) |
|  |  |  | 5.41E-09 |  | 3.40, 2 | 0.45 | 1355.62598 | 0.01641 | R.MDSTNADALYVR.G |  |
|  |  |  | 3.85E-07 |  | 2.89, 2 | 0.54 | 1443.60632 | 0.01738 | K.DYNEAYNYYTK.A |  |
|  |  |  | 4.33E05 |  | 2.78, 2 | 0.54 | 1405.72095 | 0.02873 | K.EVGEAFTILSDPK.K |  |
| 18 | *Ero1l* | **Q96HE7** | 1.22E-14 | 38.20 | 140.25 |  |  |  |  |  |
|  |  |  | 1.04E-11 |  | 4.43, 3 | 0.53 | 2074.03027 | -0.05380 | R.RDC*AVKPC*QSDEVPDGIK.S | Carbamidomethylation (C) |
|  |  |  | 1.31E-04 |  | 2.88, 2 | 0.51 | 1285.62122 | 0.00749 | R.YTGYKGPDAWK.I |  |
|  |  |  | 2.78E-07 |  | 2.78, 2 | 0.50 | 1917.92916 | -0.04642 | R.DC*AVKPC*QSDEVPDGIK.S | Carbamidomethylation (C) |
|  |  |  | 7.04E-10 |  | 3.96, 2 | 0.53 | 1505.73291 | 0.00139 | R.LGAVDESLSEETQK.A |  |
|  |  |  | 6.73E-04 |  | 1.80, 1 | 0.23 | 851.47339 | 0.00366 | R.NLLQNIH.- |  |
|  |  |  | 9.79E-08 |  | 3.04, 2 | 0.42 | 1290.63245 | 0.00298 | R.FDGILTEGEGPR.R |  |
|  |  |  | 3.82E-12 |  | 4.46, 3 | 0.52 | 2078.00114 | 0.00549 | K.LIANM#PESGPSYEFHLTR.Q | Oxidation (M) |
|  |  |  | 2.67E-08 |  | 3.30, 2 | 0.52 | 2087.00114 | 0.01331 | K.LIANM#PESGPSYEFHLTR.Q | Oxidation (M) |
|  |  |  | 1.16E-08 |  | 3.64, 2 | 0.59 | 2083.91784 | -0.02106 | K.YSEEANNLIEEC*EQAER.L | Carbamidomethylation (C) |
|  |  |  | 4.90E-07 |  | 3.71, 2 | 0.36 | 1450.79407 | 0.00713 | R.YLLQETWLEKK.W |  |
|  |  |  | 1.13E-10 |  | 3.54, 3 | 0.54 | 2062.00635 | 0.00821 | K.LIANMPESGPSYEFHLTR.Q |  |
|  |  |  | 1.22E-14 |  | 2.83, 2 | 0.49 | 2062.00635 | 0.00810 | K.LIANMPESGPSYEFHLTR.Q |  |
|  |  |  | 2.86E-06 |  | 3.07, 2 | 0.38 | 1322.69910 | 0.00456 | R.YLLQETWLEK.K |  |
|  |  |  | 1.72E-07 |  | 2.75, 2 | 0.54 | 1857.84424 | 0.01152 | K.SFPLHFDENSFFAGDK.K |  |
|  |  |  | 2.81E-04 |  | 3.42, 2 | 0.30 | 1367.79667 | 0.00404 | K.M#LLLEILHEIK.S | Oxidation (M) |
|  |  |  | 1.22E-09 |  | 3.47, 2 | 0.30 | 1380.72705 | 0.00261 | R.QEIVSLFNAFGR.I |  |
| 19 | *Ethe1* | gi14198377 | 2.64E-11 | 40.60 | 90.22 |  |  |  |  |  |
|  |  |  | 2.44E-05 |  | 3.04, 2 | 0.36 | 1054.49276 | -0.01920 | R.TDFQQGC*AK.T | Carbamidomethylation (C) |
|  |  |  | 1.90E-04 |  | 2.79, 2 | 0.31 | 940.55743 | 0.00633 | R.GGSGAPILLR.Q |  |
|  |  |  | 2.64E-11 |  | 4.25, 3 | 0.62 | 1796.87732 | 0.01719 | R.LSGAQADLHIEDGDSIR.F |  |
|  |  |  | 3.84E-11 |  | 4.17, 2 | 0.59 | 1796.87732 | 0.02593 | R.LSGAQADLHIEDGDSIR.F |  |
|  |  |  | 2.32E-10 |  | 3.77, 2 | 0.52 | 1502.74201 | 0.01866 | K.PQQIDFAVPANM#R.C | Oxidation (M) |
|  |  |  | 2.22E-04 |  | 3.11, 2 | 0.28 | 999.5655 | 0.00798 | K.IMGNLNLPK.P |  |
|  |  |  | 9.08E-07 |  | 3.09, 2 | 0.42 | 1316.72963 | -0.01975 | R.SLLPGC*QSVISR.L | Carbamidomethylation (C) |
|  |  |  | 5.90E-06 |  | 2.92, 2 | 0.39 | 1225.64384 | -0.02233 | R.LTLSC*EEFVK.I | Carbamidomethylation (C) |
|  |  |  | 1.47E-09 |  | 4.42, 2 | 0.56 | 1522.84753 | 0.01543 | R.EAVLIDPVLETAPR.D |  |
| 20 | *Exoc7* | gi173911098 | 1.17E-07 | 22.60 | 110.25 |  |  |  |  |  |
|  |  |  | 2.09E-04 |  | 2.76, 2 | 0.38 | 938.48291 | 0.56835 | K.VTDYIAEK.N |  |
|  |  |  | 4.93E-05 |  | 2.74, 2 | 0.48 | 1577.78052 | 0.36089 | K.SSSSSGVPYSPAIPNK.R |  |
|  |  |  | 1.84E-04 |  | 4.54, 3 | 0.39 | 1948.99744 | 0.93552 | K.QTKPEFDQVLQGTAASTK.N |  |
|  |  |  | 1.97E-07 |  | 3.88, 3 | 0.12 | 1688.92512 | 0.37039 | K.NKLPGLITSM#ETIGAK.A | Oxidation (M) |
|  |  |  | 2.05E-06 |  | 2.86, 3 | 0.35 | 2483.13948 | 1.08315 | K.ALEDFADNIKNDPDKEYNM#PK.D | Oxidation (M) |
|  |  |  | 9.14E-04 |  | 3.52, 3 | 0.29 | 1492.80054 | 0.22550 | K.LKQEEETLSFIR.D |  |
|  |  |  | 7.04E-05 |  | 4.18, 3 | 0.46 | 1952.88721 | 0.06809 | K.AVEYFQDNSPDSPELNK.V |  |
|  |  |  | 8.00E-07 |  | 4.63, 2 | 0.43 | 1952.88721 | -0.12984 | K.AVEYFQDNSPDSPELNK.V |  |
|  |  |  | 9.81E-05 |  | 3.98, 2 | 0.51 | 1606.73184 | 0.53457 | R.NQDFM#NVYYQIR.S | Oxidation (M) |
|  |  |  | 3.66E-07 |  | 3.98, 2 | 0.56 | 1328.77844 | 0.38005 | K.SELIQLVAVTQK.T |  |
|  |  |  | 1.17E-07 |  | 3.30, 2 | 0.43 | 1385.67293 | -0.33084 | K.NM#VSILSSFESR.L | Oxidation (M) |
|  |  |  | 2.07E-06 |  | 3.93, 2 | 0.53 | 1311.79944 | 0.42851 | K.VLGNLQLNLLSK.S |  |
| 21 | *Fkbp4; Hsp56* | gi4503729 | 4.04E-10 | 12.60 | 40.20 |  |  |  |  |  |
|  |  |  | 3.13E-07 |  | 2.63, 2 | 0.57 | 1408.61865 | -0.00020 | K.SNTAGSQSQVETEA |  |
|  |  |  | 1.06E-04 |  | 3.92, 2 | 0.57 | 1438.73003 | -0.02795 | K.LQAFSAAIESC*NK.A | Carbamidomethylation (C) |
|  |  |  | 4.04E-10 |  | 3.18, 2 | 0.45 | 1532.70752 | 0.00273 | K.GEDLTEEEDGGIIR.R |  |
|  |  |  |  |  | 2.80, 2 | 0.51 | 1950.94434 | 0.01213 | R.FEIGEGENLDLPYGLER.A |  |
| 22 | *Fth1* | gi|76779199| | 5.54E-08 | 22.00 | 50.22 |  |  |  |  |  |
|  |  |  | 5.54E-08 |  | 3.98, 3 | 0.58 | 1939.90454 | 0.02445 | K.YFLHQSHEEREHAEK.L |  |
|  |  |  | 2.84E-07 |  | 3.59, 3 | 0.45 | 1345.62842 | 0.01639 | K.YFLHQSHEER.E |  |
|  |  |  | 5.78E-07 |  | 2.93, 2 | 0.37 | 1345.62842 | 0.01885 | K.YFLHQSHEER.E |  |
|  |  |  | 1.26E-06 |  | 4.05, 3 | 0.49 | 1545.70410 | 0.01712 | R.QNYHQDSEAAINR.Q |  |
|  |  |  | 4.61E-06 |  | 2.73, 2 | 0.52 | 1545.70410 | 0.02849 | R.QNYHQDSEAAINR.Q |  |
|  |  |  | 4.62E-07 |  | 3.71, 2 | 0.46 | 1294.71143 | 0.01286 | K.NVNQSLLELHK.L |  |
|  |  |  | 1.27E-05 |  | 3.46, 2 | 0.19 | 1643.76214 | 0.04846 | K.M#GAPESGLAEYLFDK.H | Oxidation (M) |
| 23 | *Ftl* | gi|20149498| | 1.44E-14 | 43.20 | 120.31 |  |  |  |  |  |
|  |  |  | 9.48E-05 |  | 2.76, 2 | 0.41 | 1209.57930 | 0.00217 | K.M#GDHLTNLHR.L | Oxidation (M) |
|  |  |  | 2.51E-04 |  | 3.57, 3 | 0.31 | 1312.67932 | 0.01291 | K.KMGDHLTNLHR.L |  |
|  |  |  | 1.18E-04 |  | 3.22, 2 | 0.21 | 1193.58435 | 0.01103 | K.MGDHLTNLHR.L |  |
|  |  |  | 3.77E-05 |  | 3.63, 2 | 0.51 | 1718.80540 | 0.03510 | K.KPAEDEWGKTPDAM#K.A | Oxidation (M) |
|  |  |  | 1.53E-04 |  | 3.78, 3 | 0.58 | 1718.80540 | 0.02447 | K.KPAEDEWGKTPDAM#K.A | Oxidation (M) |
|  |  |  | 6.05E-07 |  | 4.25, 3 | 0.51 | 1702.81055 | 0.02689 | K.KPAEDEWGKTPDAMK.A |  |
|  |  |  | 6.73E-08 |  | 4.29, 2 | 0.51 | 1574.71558 | 0.02068 | K.PAEDEWGKTPDAMK.A |  |
|  |  |  | 2.00E-07 |  | 3.72, 2 | 0.57 | 1702.81055 | 0.02519 | K.KPAEDEWGKTPDAMK.A |  |
|  |  |  | 3.97E-09 |  | 4.21, 3 | 0.43 | 1719.98645 | 0.01566 | K.KLNQALLDLHALGSAR.T |  |
|  |  |  | 1.45E-10 |  | 6.16, 2 | 0.60 | 1719.98645 | 0.02629 | K.KLNQALLDLHALGSAR.T |  |
|  |  |  | 2.86E-07 |  | 3.69, 3 | 0.48 | 1591.89148 | 0.01242 | K.LNQALLDLHALGSAR.T |  |
|  |  |  | 8.22E-11 |  | 5.14, 2 | 0.61 | 1591.89148 | 0.03215 | K.LNQALLDLHALGSAR.T |  |
|  |  |  | 1.44E-14 |  | 3.38, 3 | 0.49 | 2345.10597 | 0.00301 | R.TDPHLC*DFLETHFLDEEVK.L | Carbamidomethylation (C) |
|  |  |  | 2.00E-06 |  | 5.18, 3 | 0.61 | 1607.80640 | 0.02384 | R.LGGPEAGLGEYLFER.L |  |
|  |  |  | 1.10E-13 |  | 4.15, 2 | 0.56 | 1607.8064 | 0.03948 | R.LGGPEAGLGEYLFER.L |  |
| 24 | *Gfpt1* | gi|205277386| | 1.50E-10 | 11.30 | 50.26 |  |  |  |  |  |
|  |  |  | 1.10E-07 |  | 3.47, 2 | 0.46 | 1694.80540 | -0.15423 | K.EIFEQPESVVNTM#R.G | Oxidation (M) |
|  |  |  | 1.60E08 |  | 3.90, 2 | 0.65 | 1632.89148 | 0.36882 | R.GALTVGITNTVGSSISR.E |  |
|  |  |  | 3.36E-04 |  | 3.23, 3 | 0.35 | 1790.89929 | 1.18741 | K.EITYMHSEGILAGELK.H |  |
|  |  |  | 8.08E-07 |  | 3.01, 2 | 0.51 | 1554.77991 | -0.50349 | R.VNFDDYTVNLGGLK.D |  |
|  |  |  | 1.50E-10 |  | 4.78, 2 | 0.44 | 1732.87524 | 0.58794 | R.VIFLEDDDVAAVVDGR.L |  |
| 25 | *Gpn1* |  | 1.15E-06 | 13.70 | 40.20 |  |  |  |  |  |
|  |  |  |  |  | 3.92, 2 | 0.48 | 1231.60254 | 0.01177 | K.SLANAESQQQR.E |  |
|  |  |  |  |  | 3.42, 2 | 0.31 | 1235.59363 | 0.01348 | K.DMGSVALDAGTAK.D |  |
|  |  |  |  |  | 2.70, 2 | 0.32 | 1076.61725 | 0.01354 | K.LPFIVVM#NK.T | Oxidation (M) |
|  |  |  |  |  | 3.74, 2 | 0.49 | 1762.96973 | 0.02471 | K.DSLSPVLHPSDLILTR.G |  |
|  |  |  |  |  |  |  |  |  |  |  |
| 26 | *Gsn* | gi|4504165| | 1.92E-10 | 9.30 | 40.26 |  |  |  |  |  |
|  |  |  | 7.77E-05 |  | 3.68, 3 | 0.42 | 2166.07470 | 0.91295 | R.VHVSEEGTEPEAM#LQVLGPK.P | Oxidation (M) |
|  |  |  | 2.04E-05 |  | 3.44, 2 | 0.44 | 1319.69543 | -0.14058 | K.AGALNSNDAFVLK.T |  |
|  |  |  | 1.99E-07 |  | 5.12, 2 | 0.65 | 1837.89673 | 0.35185 | K.TPSAAYLWVGTGASEAEK.T |  |
|  |  |  | 1.92E-10 |  | 3.79, 2 | 0.57 | 1829.96436 | 0.32732 | K.QTQVSVLPEGGETPLFK.Q |  |
| 27 | *Gstp1* | P09211 | 8.33E-14 | 66.20 | 140.28 |  |  |  |  |  |
|  |  |  | 1.60E-05 |  | 3.11, 2 | 0.42 | 1136.60739 | -0.01421 | K.ASC*LYGQLPK.F |  |
|  |  |  | 3.12E-07 |  | 3.65, 2 | 0.50 | 1276.63538 | 0.00859 | R.MLLADQGQSWK.E |  |
|  |  |  | 4.85E-05 |  | 4.62, 2 | 0.44 | 1418.72742 | 0.02556 | K.PFETLLSQNQGGK.T |  |
|  |  |  | 1.27E-07 |  | 3.37, 2 | 0.33 | 1292.63033 | 0.02072 | R.M#LLADQGQSWK.E | Oxidation (M) |
|  |  |  | 2.91E-08 |  | 4.09, 3 | 0.45 | 1733.85925 | 0.01987 | K.EEVVTVETWQEGSLK.A |  |
|  |  |  | 3.51E-09 |  | 4.21, 2 | 0.57 | 1534.77881 | 0.01811 | K.YISLIYTNYEAGK.D |  |
|  |  |  | 1.17E-06 |  | 3.84, 2 | 0.49 | 1484.76062 | 0.01604 | -.M#PPYTVVYFPVR.G | Oxidation (M) |
|  |  |  | 8.33E-14 |  | 4.83, 2 | 0.60 | 2126.16040 | 0.003765 | K.ALPGQLKPFETLLSQNQGGK.T |  |
|  |  |  | 9.65E-08 |  | 4.00, 3 | 0.53 | 1883.94971 | 0.02097 | K.FQDGDLTLYQSNTILR.H |  |
|  |  |  | 9.18E-11 |  | 4.48, 3 | 0.54 | 2126.16040 | 0.02146 | K.ALPGQLKPFETLLSQNQGGK.T |  |
|  |  |  | 4.65E-09 |  | 4.37, 2 | 0.50 | 1903.99121 | 0.02678 | K.AFLASPEYVNLPINGNGK.Q |  |
|  |  |  | 1.52E-13 |  | 5.08, 3 | 0.55 | 2132.97644 | 0.02408 | K.DQQEAALVDM#VNDGVEDLR.C |  |
|  |  |  | 1.57E-08 |  | 3.72, 2 | 0.60 | 1468.76575 | 0.02471 | -.MPPYTVVYFPVR.G |  |
|  |  |  | 2.02E-13 |  | 4.67, 2 | 0.53 | 2132.97644 | 0.02532 | K.DQQEAALVDM#VNDGVEDLR.C | Oxidation (M) |
|  |  |  | 6.06E-11 |  | 4.35, 2 | 0.56 | 1733.85225 | 0.03337 | K.EEVVTVETWQEGSLK.A |  |
|  |  |  | 1.59E-12 |  | 4.74, 2 | 0.64 | 2116.98145 | 0.03813 | K.DQQEAALVDMVNDGVEDLR.C |  |
|  |  |  | 1.50E-09 |  | 5.66, 3 | 0.64 | 2116.98145 | 0.02994 | K.DQQEAALVDMVNDGVEDLR.C |  |
|  |  |  | 1.42E-12 |  | 5.04, 2 | 0.52 | 1883.94971 | 0.03484 | K.FQDGDLTLYQSNTILR.H |  |
| 28 | *Habp1/* C1qbp | gi|4502491| | 1.47E-11 | 24.40 | 40.23 |  |  |  |  |  |
|  |  |  | 1.72E-08 |  | 2.89, 2 | 0.49 | 1513.68054 | 0.02068 | R.EVSFQSTGESEWK.D |  |
|  |  |  | 1.02E-08 |  | 2.83, 2 | 0.42 | 1637.74755 | 0.02533 | K.M#SGGWELELNGTEAK.L | Oxidation (M) |
|  |  |  | 1.47E-11 |  | 3.42, 2 | 0.33 | 1621.75269 | 0.01909 | K.MSGGWELELNGTEAK.L |  |
|  |  |  | 4.95E-09 |  | 4.17, 2 | 0.53 | 2287.17041 | 0.05791 | K.VEEQEPELTSTPNFVVEVIK.N |  |
| 29 | *Hist1h4e* | P62805 | 3.68E-07 | 31.10 | 30.20 |  |  |  |  |  |
|  |  |  | 4.29E-06 |  | 2.74, 2 | 0.48 | 1134.54260 | 0.00737 | R.DAVTYTEHAK.R |  |
|  |  |  | 5.30E-06 |  | 3.55, 2 | 0.17 | 1325.75354 | 0.01579 | R.DNIQGITKPAIR.R |  |
|  |  |  | 3.68E-07 |  | 3.83, 2 | 0.45 | 1180.62085 | 0.01128 | R.ISGLIYEETR.G |  |
| 30 | *Hpxel* | gi|564065| | 1.09E-08 | 15.24 | 50.20 |  |  |  |  |  |
|  |  |  | 2.21E-07 |  | 3.78, 2 | 0.56 | 1454.83386 | 0.01958 | K.HVLHVQLNRPNK.R |  |
|  |  |  | 3.80E-05 |  | 2.76, 1 | 0.35 | 801.48285 | 0.00909 | R.AVVISGAGK.M |  |
|  |  |  | 2.03E-06 |  | 2.78, 2 | 0.47 | 801.48285 | 0.00591 | R.AVVISGAGK.M |  |
|  |  |  | 6.22E-05 |  | 3.86, 3 | 0.49 | 1454.83386 | 0.01679 | K.HVLHVQLNRPNK.R |  |
|  |  |  | 8.40E-04 |  | 1.96, 1 | 0.37 | 1056.47941 | -0.01396 | R.EMVEC*FNK.I | Carbamidomethylation (C) |
|  |  |  | 7.30E-08 |  | 2.68, 2 | 0.45 | 1298.63757 | 0.01982 | R.YQETFNVIER.C |  |
|  |  |  | 1.09E-08 |  | 2.91, 2 | 0.50 | 1376.66088 | -0.00788 | R.YC*AQDAFFQVK.E | Carbamidomethylation (C) |
| 31 | *Hspb1* | P04792 | 1.00E-30 | 63.90 | 100.36 |  |  |  |  |  |
|  |  |  | 4.90E-05 |  | 2.84, 2 | 0.50 | 941.50507 | 0.01250 | R.AQLGGPEAAK.S |  |
|  |  |  | 5.55E-08 |  | 3.27, 2 | 0.41 | 1146.63647 | 0.01677 | K.TKDGVVEITGK.H |  |
|  |  |  | 1.29E-04 |  | 2.28, 2 | 0.31 | 960.43225 | 0.01238 | R.DWYPHSR.L |  |
|  |  |  | 3.91E-05 |  | 2.48, 1 | 0.46 | 917.49384 | 0.01691 | K.DGVVEITGK.H |  |
|  |  |  | 2.55E-05 |  | 3.43, 2 | 0.46 | 917.49384 | 0.01586 | K.DGVVEITGK.H |  |
|  |  |  | 5.44E-07 |  | 2.79, 2 | 0.21 | 1075.57422 | 0.01006 | R.QLSSGVSEIR.H |  |
|  |  |  | 7.25E-12 |  | 5.61, 3 | 0.59 | 1878.01196 | 0.01548 | R.PLPPAAIESPAVAAPAYSR.A |  |
|  |  |  | 7.40E-07 |  | 3.53, 3 | 0.56 | 1783.92249 | 0.01548 | R.VSLDVNHFAPDELTVK.T |  |
|  |  |  | 3.33E-15 |  | 4.75, 2 | 0.64 | 1878.01196 | 0.02617 | R.PLPPAAIESPAVAAPAYSR.A |  |
|  |  |  | 1.46E-08 |  | 3.50, 2 | 0.58 | 1905.99158 | 0.00847 | K.LATQSNEITIPVTFESR.A |  |
|  |  |  | 4.69E-05 |  |  |  |  |  | K.LATQSNEITIPVTFESR.A |  |
|  |  |  | 1.00E-30 |  | 7.30, 3 | 0.61 | 3242.65007 | 0.05290 | R.KYTLPPGVDPTQVSSSLSPEGTLTVEAPM#PK.L | Oxidation (M) |
|  |  |  | 4.94E-06 |  | 4.28, 2 | 0.47 | 1163.62073 | 0.00578 | R.LFDQAFGLPR.L |  |
|  |  |  | 1.44E-04 |  | 2.64, 1 | 0.23 | 1163.62073 | 0.01514 | R.LFDQAFGLPR.L |  |
|  |  |  | 2.13E-10 |  | 3.77, 2 | 0.58 | 1783.92249 | 0.02104 | R.VSLDVNHFAPDELTVK.T |  |
| 32 | Hspd1/Hsp60 | gi|77702086 | | 1.11E-15 | 43.65 | 200.31 |  |  |  |  |  |
|  |  |  | 8.41E-07 |  | 3.54, 2 | 0.39 | 1361.69067 | 0.01592 | K.VGGTSDVEVNEKK.D |  |
|  |  |  | 9.11E-06 |  | 3.24, 1 | 0.46 | 1233.59570 | 0.01611 | K.VGGTSDVEVNEK.K |  |
|  |  |  | 8.45E-06 |  | 2.80, 2 | 0.51 | 833.39001 | 0.00572 | K.APGFGDNR.K |  |
|  |  |  | 9.13E-04 |  | 2.83, 2 | 0.12 | 941.61426 | 0.00682 | K.IGIEIIKR.T |  |
|  |  |  | 7.88E-05 |  | 3.27, 2 | 0.35 | 901.53534 | 0.00658 | K.LSDGVAVLK.V |  |
|  |  |  | 5.94E-07 |  | 3.53, 2 | 0.43 | 1153.76672 | 0.00969 | R.LKVGLQVVAVK.A |  |
|  |  |  | 1.51E-06 |  | 3.10, 2 | 0.40 | 1233.59570 | 0.01531 | K.VGGTSDVEVNEK.K |  |
|  |  |  | 1.11E-15 |  | 5.16, 2 | 0.62 | 2560.24854 | 0.04228 | K.LVQDVANNTNEEAGDGTTTATVLAR.S |  |
|  |  |  | 9.84E-05 |  | 3.52, 2 | 0.44 | 1206.60345 | 0.01147 | K.EIGNIISDAM#K.K |  |
|  |  |  | 2.54E-06 |  | 3.58, 2 | 0.41 | 1344.71582 | 0.00774 | R.TVIIEQSWGSPK.V |  |
|  |  |  | 6.85E-06 |  | 3.60, 3 | 0.57 | 2560.24854 | 0.03629 | K.LVQDVANNTNEEAGDGTTTATVLAR.S |  |
|  |  |  | 2.37E-04 |  | 3.56, 2 | 0.28 | 912.58771 | 0.00688 | K.VGLQVVAVK.A |  |
|  |  |  | 7.65E-11 |  | 3.32, 2 | 0.52 | 2556.09382 | 0.05271 | K.IM#QSSSEVGYDAM#AGDFVNM#VEK.G |  |
|  |  |  | 9.99E-15 |  | 5.45, 3 | 0.57 | 2047.16589 | 0.03012 | K.KISSIQSIVPALEIANAHR.K |  |
|  |  |  | 1.12E-11 |  | 5.48, 2 | 0.70 | 2041.04480 | 0.03179 | K.PVTTPEEIAQVATISANGDK.E |  |
|  |  |  | 1.65E-09 |  | 3.45, 2 | 0.47 | 1389.70483 | 0.02397 | R.GYISPYFINTSK.G |  |
|  |  |  | 9.20E-10 |  | 4.71, 2 | 0.55 | 1684.93560 | -0.00550 | R.AAVEEGIVLGGGC*ALLR.C |  |
|  |  |  | 1.35E-07 |  | 5.98, 3 | 0.50 | 1684.93560 | -0.01723 | R.AAVEEGIVLGGGC*ALLR.C |  |
|  |  |  | 3.22E-12 |  | 4.48, 2 | 0.58 | 2038.02271 | 0.03703 | R.IQEIIEQLDVTTSEYEK.E |  |
|  |  |  | 5.73E-09 |  | 4.51, 2 | 0.62 | 1601.78212 | -0.01767 | K.C*EFQDAYVLLSEK.K |  |
|  |  |  | 5.28E-04 |  |  |  |  |  | K.C*EFQDAYVLLSEK.K |  |
|  |  |  | 3.21E-13 |  | 5.12, 3 | 0.60 | 2145.12936 | 0.02387 | R.ALM#LQGVDLLADAVAVTM#GPK.G |  |
|  |  |  | 3.34E-08 |  | 3.06, 3 | 0.44 | 1919.07092 | 0.01389 | K.ISSIQSIVPALEIANAHR.K |  |
| 33 | *Il1rn* | gi|27894319| | 8.88E-15 | 30.50 | 40.30 |  |  |  |  |  |
|  |  |  | 1.69E-10 |  | 4.80, 2 | 0.51 | 1842.99194 | 0.02654 | R.LQLEAVNITDLSENRK.Q |  |
|  |  |  | 7.13E-10 |  | 5.33, 3 | 0.64 | 2100.07202 | 0.02738 | R.NNQLVAGYLQGPNVNLEEK.I |  |
|  |  |  | 9.65E-12 |  | 4.33, 2 | 0.59 | 2012.13281 | 0.03032 | K.IDVVPIEPHALFLGIHGGK.M |  |
|  |  |  | 4.69E-08 |  | 4.95, 3 | 0.60 | 1714.89697 | 0.02433 | R.LQLEAVNITDLSENR.K |  |
|  |  |  | 2.81E-13 |  | 4.09, 2 | 0.67 | 1714.89697 | 0.02361 | R.LQLEAVNITDLSENR.K |  |
|  |  |  | 8.88E-15 |  | 4.87, 2 | 0.53 | 2100.07202 | 0.04546 | R.NNQLVAGYLQGPNVNLEEK.I |  |
| 34 | *Ivl* | gi|44890059| | 6.55E-08 | 16.90 | 770.22 |  |  |  |  |  |
|  |  |  | 5.43E-06 |  | 3.42, 2 | 0.34 | 1209.62219 | 0.00994 | K.HLEQQEGQLK.H |  |
|  |  |  | 2.64E-05 |  | 2.92, 2 | 0.38 | 1473.69437 | -0.00951 | K.GLPEQEC*EQQQK.E | Carbamidomethylation (C) |
|  |  |  | 3.14E-04 |  | 2.70, 2 | 0.25 | 1056.53198 | 0.00994 | K.AENPEQQLK.Q |  |
|  |  |  | 2.79E-05 |  | 3.19, 2 | 0.45 | 1235.62659 | 0.01323 | K.YLEQQEGQLK.H |  |
|  |  |  | 2.38E-05 |  | 3.59, 3 | 0.45 | 1806.98474 | 0.02561 | K.VPVELPVEVPSKQEEK.H |  |
|  |  |  | 3.89E-04 |  | 3.78, 3 | 0.47 | 2393.15796 | 0.04734 | K.HLEQQEGQLEQLEEQEGQLK.H |  |
|  |  |  | 6.55E-08 |  | 3.49, 2 | 0.32 | 1441.78967 | 0.01921 | K.LLDQQLDQELVK.R |  |
| 35 | *Lgals3bp* | gi|5031863| | 5.22E-08 | 11.62 | 60.21 |  |  |  |  |  |
|  |  |  | 5.22E-08 |  | 3.92, 2 | 0.53 | 1059.51770 | 0.02287 | R.LADGGATNQGR.V |  |
|  |  |  | 5.61E-04 |  | 2.73, 2 | 0.45 | 1029.53235 | 0.00981 | R.STHTLDLSR.E |  |
|  |  |  | 9.67E-08 |  | 3.71, 2 | 0.50 | 1326.65356 | 0.01421 | R.ASHEEVEGLVEK.I |  |
|  |  |  | 2.74E-05 |  | 2.90, 2 | 0.44 | 975.57208 | 0.00841 | R.IDITLSSVK.C |  |
|  |  |  | 5.42E-04 |  | 2.73, 2 | 0.38 | 1355.77808 | 0.01592 | R.SDLAVPSELALLK.A |  |
|  |  |  | 9.28E-08 |  | 3.50, 2 | 0.49 | 1592.79150 | 0.02361 | R.ELSEALGQIFDSQR.G |  |
| 36 | *Lima1* | gi|187952247| | 1.92E-08 | 28.88 | 100.24 |  |  |  |  |  |
|  |  |  | 1.19E-06 |  | 3.22, 2 | 0.48 | 1534.69800 | 0.62883 | R.STPAEDDSRDSQVK.S |  |
|  |  |  | 6.63E-04 |  | 2.70, 2 | 0.34 | 1089.54224 | -0.15193 | R.ASSLSESSPPK.A |  |
|  |  |  | 3.36E-04 |  | 3.56, 3 | 0.46 | 1900.98755 | 0.28733 | K.SEVQQPVHPKPLSPDSR.A |  |
|  |  |  | 2.56E-04 |  | 2.71, 2 | 0.19 | 1230.66882 | 0.63579 | K.ELSVEEQIKR.N |  |
|  |  |  | 3.04E-06 |  | 3.64, 2 | 0.33 | 1173.62219 | 0.53081 | K.ISANENSLAVR.S |  |
|  |  |  | 1.92E-08 |  | 3.79, 2 | 0.45 | 1075.58167 | 0.65959 | K.VGVLAASMEAK.A |  |
|  |  |  | 6.24E-06 |  | 2.88, 2 | 0.48 | 1374.68518 | 0.46575 | K.LSLGTYASLHGR.I |  |
|  |  |  | 2.67E-07 |  | 4.58, 3 | 0.55 | 1712.89661 | 1.22123 | R.SRPFTVAASFQSTSVK.S |  |
|  |  |  | 1.03E-05 |  | 3.58, 3 | 0.46 | 1966.99414 | 0.69199 | K.NENEEILERPAQLANAR.E |  |
|  |  |  | 1.14E-06 |  | 4.72, 2 | 0.49 | 1517.74817 | 0.61492 | K.SQDVELWEGEVVK.E |  |
| 37 | *Mmab* | gi|16418349| | 2.81E-07 | 24.00 | 60.19 |  |  |  |  |  |
|  |  |  | 8.26E-06 |  | 3.25, 2 | 0.51 | 1653.78271 | 0.02397 | R.GPQGVEDGDRPQPSSK.T |  |
|  |  |  | 3.47E-05 |  | 3.36, 2 | 0.35 | 1090.57676 | -0.02227 | K.ISSALHFC*R.A | Carbamidomethylation (C) |
|  |  |  | 8.75E-06 |  | 3.12, 2 | 0.55 | 1088.50073 | 0.01018 | K.GFSSTFTGER.R |  |
|  |  |  | 2.81E-07 |  | 2.74, 2 | 0.48 | 1686.87308 | 0.02491 | R.VVPLVQM#GETDANVAK.F | Oxidation (M) |
|  |  |  | 1.43E-06 |  | 3.47, 2 | 0.58 | 1670.87817 | 0.03142 | R.VVPLVQMGETDANVAK.F |  |
|  |  |  | 5.25E-05 |  | 3.36, 2 | 0.32 | 1198.64661 | 0.01641 | R.LSDYLFTLAR.Y |  |
| 38 | *Mtpn* | gi21956645 | 5.43E-09 | 25.40 | 20.23 |  |  |  |  |  |
|  |  |  | 6.24E-08 |  | 3.06, 2 | 0.50 | 1465.71692 | 0.73235 | K.NGDLDEVKDYVAK.G |  |
|  |  |  | 5.43E-09 |  | 4.65, 2 | 0.65 | 1747.84973 | 1.39812 | K.GPDGLTAFEATDNQAIK.A |  |
| 39 | *Ndrg1* | **Q92597** | 5.55E-15 | 31.20 | 156.35 |  |  |  |  |  |
|  |  |  | 1.72E-10 |  | 5.94, 3 | 0.70 | 2376.11743 | 0.02976 | R.SHTSEGAHLDITPNSGAAGNSAGPK.S |  |
|  |  |  | 5.55E-15 |  | 6.52, 2 | 0.67 | 2376.11743 | 0.03667 | R.SHTSEGAHLDITPNSGAAGNSAGPK.S |  |
|  |  |  | 7.72E-10 |  | 3.12, 2 | 0.58 | 1736.79081 | 0.01197 | K.EEM#QSNVEVVHTYR.Q | Oxidation (M) |
|  |  |  | 3.61E-06 |  | 3.15, 1 | 0.47 | 1338.64954 | 0.01526 | R.TASGSSVTSLDGTR.S |  |
|  |  |  | 2.26E-06 |  | 4.30, 3 | 0.47 | 1981.00723 | 0.00953 | K.GNRPVILTYHDIGM#NHK.T | Oxidation (M) |
|  |  |  | 1.18E-09 |  | 3.96, 2 | 0.60 | 1981.00723 | 0.01895 | K.GNRPVILTYHDIGM#NHK.T | Oxidation (M) |
|  |  |  | 8.18E-10 |  | 3.46, 2 | 0.60 | 1720.79590 | 0.00884 | K.EEMQSNVEVVHTYR.Q |  |
|  |  |  | 3.10E-08 |  | 4.57, 2 | 0.50 | 1965.01233 | 0.01543 | K.GNRPVILTYHDIGMNHK.T |  |
|  |  |  | 2.54E-09 |  | 3.20, 2 | 0.53 | 1588.77631 | -0.01381 | K.M#ADC*GGLPQISQPAK.L | Oxidation (M) |
|  |  |  | 2.56E-09 |  | 4.08, 3 | 0.52 | 1965.01233 | 0.01310 | K.GNRPVILTYHDIGMNHK.T |  |
|  |  |  | 6.79E-09 |  | 2.69, 2 | 0.64 | 1873.79171 | 0.01693 | K.YFVQGM#GYM#PSASM#TR.L | Oxidation (M) |
|  |  |  | 3.47E-05 |  | 2.52, 3 | 0.30 | 1588.77631 | -0.02255 | K.M#ADC*GGLPQISQPAK.L |  |
|  |  |  | 8.87E-07 |  | 2.82, 1 | 0.55 | 1572.78141 | 0.00900 | K.MADC*GGLPQISQPAK.L |  |
|  |  |  | 2.13E-04 |  | 3.56, 3 | 0.53 | 1572.78141 | -0.01954 | K.MADC*GGLPQISQPAK.L |  |
|  |  |  | 1.05E-08 |  | 3.26, 2 | 0.54 | 1572.78141 | -0.02428 | K.MADC*GGLPQISQPAK.L |  |
|  |  |  | 1.10E-11 |  | 2.77, 2 | 0.40 | 1857.79681 | 0.02050 | K.YFVQGM#GYM#PSASMTR.L | Oxidation (M) |
|  |  |  | 5.57E-11 |  | 3.10, 2 | 0.33 | 1857.79681 | 0.02221 | K.YFVQGMGYM#PSASM#TR.L | Oxidation (M) |
|  |  |  | 1.14E-10 |  | 2.83, 2 | 0.22 | 1841.80191 | 0.03615 | K.YFVQGM#GYMPSASMTR.L | Oxidation (M) |
|  |  |  | 3.78E-07 |  | 3.82, 3 | 0.55 | 2583.25210 | 0.03810 | R.QHIVNDM#NPGNLHLFINAYNSR.R | Oxidation (M) |
|  |  |  | 3.83E-13 |  | 2.97, 2 | 0.22 | 1841.80191 | 0.02065 | K.YFVQGMGYMPSASM#TR.L | Oxidation (M) |
|  |  |  | 5.98E-10 |  | 3.30, 2 | 0.53 | 1338.64954 | 0.01531 | R.TASGSSVTSLDGTR.S |  |
|  |  |  | 1.44E-14 |  | 3.38, 2 | 0.71 | 1825.80701 | 0.00994 | K.YFVQGMGYMPSASMTR.L |  |
|  |  |  |  |  |  |  |  |  |  |  |
|  |  |  |  |  |  |  |  |  |  |  |
| 40 | *Nit2* | gi79160084 | 1.11E-09 | 31.20 | 80.20 |  |  |  |  |  |
|  |  |  | 8.72E-04 |  | 2.53, 2 | 0.40 | 1227.60378 | 0.01308 | K.RSDLYAVEM#K.K | Oxidation (M) |
|  |  |  | 1.12E-04 |  | 3.43, 2 | 0.35 | 1071.50267 | 0.01227 | R.SDLYAVEM#K.K | Oxidation (M) |
|  |  |  | 5.56E-08 |  | 3.82, 2 | 0.45 | 1561.79688 | 0.01641 | R.AVDNQVYVATASPAR.D |  |
|  |  |  | 7.90E-05 |  | 3.42, 2 | 0.42 | 1055.50781 | 0.01201 | R.SDLYAVEMK.K |  |
|  |  |  | 4.18E-06 |  | 3.89, 2 | 0.24 | 1309.68994 | 0.01482 | R.FAELAQIYAQR.G |  |
|  |  |  | 2.55E-04 |  | 2.71, 2 | 0.52 | 1681.85596 | -0.0042 | K.IVSLPEC*FNSPYGAK.Y | Carbamidomethylation (C) |
|  |  |  | 7.32E-07 |  | 3.87, 2 | 0.58 | 1937.94662 | -0.001322 | K.EC*SIYLIGGSIPEEDAGK.L | Carbamidomethylation (C) |
|  |  |  | 1.11E-09 |  | 3.10, 2 | 0.57 | 1839.96148 | 0.01183 | K.LYNTC*AVFGPDGTLLAK.Y | Carbamidomethylation (C) |
| 41 | *Pdia3* | gi21361657 | 9.55E-14 | 56.30 | 390.29 |  |  |  |  |  |
|  |  |  | 1.03E-06 |  | 2.75, 1 | 0.50 | 1397.63946 | -0.03131 | K.VDC*TANTNTC*NK.Y | Carbamidomethylation (C) |
|  |  |  | 9.52E-05 |  | 3.76, 3 | 0.38 | 1315.66406 | 0.01215 | R.PSHLTNKFEDK.T |  |
|  |  |  | 2.08E-07 |  | 3.41, 2 | 0.35 | 1315.66406 | 0.01933 | R.PSHLTNKFEDK.T |  |
|  |  |  | 4.31E-07 |  | 3.57, 2 | 0.53 | 1397.63946 | -0.03956 | K.VDC*TANTNTC*NK.Y | Carbamidomethylation (C) |
|  |  |  | 6.64E-05 |  | 2.73, 2 | 0.48 | 1707.92761 | 0.02995 | R.EATNPPVIQEEKPKK.K |  |
|  |  |  | 4.31E-04 |  | 3.19, 2 | 0.36 | 1123.65820 | 0.01653 | K.KQAGPASVPLR.T |  |
|  |  |  | 1.11E-07 |  | 4.05, 2 | 0.53 | 1168.66846 | 0.00432 | R.TADGIVSHLKK.Q |  |
|  |  |  | 7.23E-09 |  | 4.47, 2 | 0.50 | 1458.71106 | 0.01213 | K.FEDKTVAYTEQK.M |  |
|  |  |  | 1.66E-06 |  | 5.01, 3 | 0.43 | 1458.71106 | 0.00779 | K.FEDKTVAYTEQK.M |  |
|  |  |  | 2.18E-05 |  | 3.82, 3 | 0.35 | 1347.70154 | 0.00669 | K.RLAPEYEAAATR.L |  |
|  |  |  | 2.61E-06 |  | 2.59, 1 | 0.58 | 1236.51270 | 0.01599 | R.DGEEAGAYDGPR.T |  |
|  |  |  | 1.52E-08 |  | 4.59, 2 | 0.54 | 1347.70154 | 0.00994 | K.RLAPEYEAAATR.L |  |
|  |  |  | 8.69E-05 |  | 2.56, 1 | 0.18 | 1197.72009 | 0.01270 | K.LSKDPNIVIAK.M |  |
|  |  |  | 1.16E-08 |  | 3.34, 2 | 0.53 | 1236.51273 | 0.00774 | R.DGEEAGAYDGPR.T |  |
|  |  |  | 2.34E-04 |  | 1.91, 1 | 0.41 | 995.56323 | 0.01300 | K.QAGPASVPLR.T |  |
|  |  |  | 2.12E-05 |  | 2.77, 1 | 0.48 | 1368.66418 | 0.01672 | K.SEPIPESNDGPVK.V |  |
|  |  |  | 9.78E-12 |  | 3.13, 2 | 0.29 | 1579.83264 | 0.01616 | R.EATNPPVIQEEKPK.K |  |
|  |  |  | 4.58E-09 |  | 5.59, 3 | 0.49 | 1652.76636 | 0.01096 | K.IFRDGEEAGAYDGPR.T |  |
|  |  |  | 8.74E-04 |  | 2.20, 1 | 0.24 | 869.50909 | 0.00897 | K.DPNIVIAK.M |  |
|  |  |  | 9.49E-11 |  | 4.47, 2 | 0.57 | 1652.76636 | 0.01225 | K.IFRDGEEAGAYDGPR.T |  |
|  |  |  | 2.69E-08 |  | 4.66, 3 | 0.52 | 1773.97583 | 0.01621 | K.KFLDAGHKLNFAVASR.K |  |
|  |  |  | 2.60E-07 |  | 3.23, 2 | 0.45 | 1040.57349 | 0.00823 | R.TADGIVSHLK.K |  |
|  |  |  | 2.55E-04 |  | 2.46, 1 | 0.39 | 1084.56738 | 0.01587 | K.YGVSGYPTLK.I |  |
|  |  |  | 6.14E-05 |  | 2.91, 2 | 0.41 | 877.48901 | 0.00463 | K.LNFAVASR.K |  |
|  |  |  | 5.12E-11 |  | 4.30, 2 | 0.45 | 1664.75842 | 0.01506 | K.MDATANDVPSPYEVR.G |  |
|  |  |  | 1.11E-11 |  | 4.69, 2 | 0.50 | 1645.88086 | 0.02324 | K.FLDAGHKLNFAVASR.K |  |
|  |  |  | 1.56E-06 |  | 2.75, 2 | 0.45 | 995.56323 | 0.00768 | K.QAGPASVPLR.T |  |
|  |  |  | 3.00E-06 |  | 2.70, 2 | 0.26 | 1172.54053 | 0.02039 | K.FVMQEEFSR.D |  |
|  |  |  | 1.07E-09 |  | 5.86, 3 | 0.64 | 2991.50081 | 0.03401 | K.KFIQENIFGIC*PHM#TEDNKDLIQGK.D | Carbamidomethylation (C), Oxidation (M) |
|  |  |  | 2.75E-08 |  | 4.17, 2 | 0.50 | 1680.75336 | 0.01829 | K.M#DATANDVPSPYEVR.G | Oxidation (M) |
|  |  |  | 3.22E-09 |  | 3.73, 2 | 0.41 | 1515.75903 | 0.01396 | R.FLQDYFDGNLKR.Y |  |
|  |  |  | 9.40E-08 |  | 2.73, 2 | 0.45 | 1368.66418 | 0.01286 | K.SEPIPESNDGPVK.V |  |
|  |  |  | 5.77E-11 |  | 4.89, 3 | 0.50 | 2863.40585 | 0.00697 | K.FIQENIFGIC*PHM#TEDNKDLIQGK.D | Carbamidomethylation (C), Oxidation (M) |
|  |  |  | 1.34E-10 |  | 3.83, 2 | 0.60 | 1832.90247 | 0.01897 | K.VVVAENFDEIVNNENK.D |  |
|  |  |  | 3.60E-05 |  | 4.06, 3 | 0.53 | 1832.90247 | 0.01639 | K.VVVAENFDEIVNNENK.D |  |
|  |  |  | 2.33E-12 |  | 4.93, 3 | 0.58 | 2847.41095 | 0.01145 | K.FIQENIFGIC*PHMTEDNKDLIQGK.D | Carbamidomethylation (C) |
|  |  |  | 7.13E-07 |  | 3.62, 3 | 0.49 | 2193.04087 | -0.00171 | K.FIQENIFGIC*PHMTEDNK.D | Carbamidomethylation (C) |
|  |  |  | 7.35E-06 |  | 2.81, 1 | 0.34 | 1341.68372 | 0.02148 | R.GFPTIYFSPANK.K |  |
|  |  |  | 5.97E-07 |  | 2.26, 1 | 0.33 | 1359.65796 | 0.02600 | R.FLQDYFDGNLK.R |  |
|  |  |  | 9.55E-14 |  | 4.66, 3 | 0.61 | 2703.39893 | 0.03757 | R.KTFSHELSDFGLESTAGEIPVVAIR.T |  |
|  |  |  | 1.07E-07 |  | 3.42, 2 | 0.46 | 1341.68372 | 0.01253 | R.GFPTIYFSPANK.K |  |
|  |  |  | 6.81E-07 |  | 3.24, 2 | 0.40 | 1359.65796 | 0.01372 | R.FLQDYFDGNLK.R |  |
|  |  |  | 1.36E-09 |  | 4.81, 3 | 0.46 | 2575.30396 | 0.03470 | K.TFSHELSDFGLESTAGEIPVVAIR.T |  |
|  |  |  | 5.26E-05 |  | 2.42, 1 | 0.46 | 1370.69507 | 0.02734 | R.ELSDFISYLQR.E |  |
|  |  |  | 1.12E-05 |  | 4.48, 3 | 0.51 | 1370.69507 | 0.00965 | R.ELSDFISYLQR.E |  |
|  |  |  | 5.09E-09 |  | 3.64, 2 | 0.48 | 1619.78394 | 0.01262 | K.DLLIAYYDVDYEK.N |  |
|  |  |  | 9.16E-06 |  | 2.83, 2 | 0.45 | 1188.53537 | 0.01070 | K.FVM#QEEFSR.D | Oxidation (M) |
|  |  |  | 7.15E-07 |  | 2.79, 2 | 0.42 | 1370.69507 | 0.01348 | R.ELSDFISYLQR.E |  |
|  |  |  | 2.09E-07 |  | 3.37, 2 | 0.50 | 1084.56738 | 0.00920 | K.YGVSGYPTLK.I |  |
|  |  |  | 9.56E-07 |  | 2.71, 2 | 0.45 | 1172.54053 | 0.00945 | K.FVMQEEFSR.D |  |
|  |  |  | 1.09E-07 |  | 2.79, 2 | 0.19 | 1197.72009 | 0.01103 | K.LSKDPNIVIAK.M |  |
| 42 | *Pgm2* | gi62898035 | 2.20E-12 | 28.10 | 220.24 |  |  |  |  |  |
|  |  |  | 1.54E-04 |  | 2.97, 2 | 0.36 | 1399.79041 | 0.51799 | R.LIAEGNKEELRK.C |  |
|  |  |  | 3.96E-10 |  | 4.37, 2 | 0.40 | 1582.72314 | 1.31658 | R.DLTTGYDDSQPDKK.A |  |
|  |  |  | 1.19E-04 |  | 3.08, 2 | 0.24 | 1130.65283 | -0.04097 | K.NSLTLEAVKR.L |  |
|  |  |  | 1.59E-06 |  | 3.18, 2 | 0.36 | 1198.60620 | 0.79131 | R.IVLANDPDADR.L |  |
|  |  |  | 5.13E-06 |  | 3.97, 2 | 0.52 | 1454.62817 | 0.08257 | R.DLTTGYDDSQPDK.K |  |
|  |  |  | 6.05E-05 |  | 2.99, 2 | 0.33 | 997.47713 | -0.05862 | R.M#EFGTAGLR.A | Oxidation (M) |
|  |  |  | 1.95E-05 |  | 3.47, 2 | 0.59 | 1334.61441 | 0.21108 | K.DTYM#LSSTVSSK.I | Oxidation (M) |
|  |  |  | 2.41E-07 |  | 4.02, 3 | 0.48 | 1733.86258 | 1.12543 | R.SALKDTYM#LSSTVSSK.I | Oxidation (M) |
|  |  |  | 3.07E-09 |  | 4.40, 2 | 0.60 | 1733.86258 | 0.38454 | R.SALKDTYM#LSSTVSSK.I | Oxidation (M) |
|  |  |  | 6.07E-04 |  | 2.63, 2 | 0.31 | 974.55170 | 0.14208 | K.NSLTLEAVK.R |  |
|  |  |  | 1.86E-04 |  | 3.43, 3 | 0.43 | 1717.86768 | -0.10659 | R.SALKDTYMLSSTVSSK.I |  |
|  |  |  | 2.08E-06 |  | 4.33, 2 | 0.61 | 1717.86768 | 0.45781 | R.SALKDTYMLSSTVSSK.I |  |
|  |  |  | 2.10E-07 |  | 4.06, 2 | 0.52 | 1318.61951 | 0.75493 | K.DTYMLSSTVSSK.I |  |
|  |  |  | 9.76E-04 |  | 2.78, 2 | 0.21 | 1456.74707 | 1.43694 | K.AIYVEYGYHITK.A |  |
|  |  |  | 1.72E-05 |  | 2.77, 2 | 0.38 | 1144.55212 | -0.14790 | K.DPDPEFPTVK.Y |  |
|  |  |  | 6.14E-08 |  | 4.30, 2 | 0.48 | 1809.97046 | 1.28337 | R.IVLANDPDADRLAVAEK.Q |  |
|  |  |  | 1.22E-04 |  | 3.05, 2 | 0.34 | 981.48224 | 0.46105 | R.MEFGTAGLR.A |  |
|  |  |  | 8.92E-07 |  | 3.75, 2 | 0.30 | 1403.75293 | 0.58257 | R.WDKNSLTLEAVK.R |  |
|  |  |  | 2.20E-10 |  | 4.58, 2 | 0.60 | 1742.84961 | 0.37944 | K.VYWDNGAQIISPHDK.G |  |
|  |  |  | 3.88E-06 |  | 3.73, 2 | 0.41 | 1259.63782 | 0.00322 | R.LDQETAQWLR.W |  |
|  |  |  | 7.96E-05 |  | 2.78, 2 | 0.50 | 977.54144 | 0.60491 | K.GIVISFDAR.A |  |
|  |  |  | 3.66E-06 |  | 2.83, 2 | 0.50 | 1539.83530 | 0.45598 | K.AFDLVPPEAVPEQK.D |  |
|  |  |  | 3.19E-07 |  | 3.51, 3 | 0.46 | 1539.83530 | 0.12375 | K.AFDLVPPEAVPEQK.D |  |
|  |  |  | 3.91E-04 |  | 3.91, 2 | 0.43 | 1234.70422 | 0.60259 | K.GVLTLSFALADK.T |  |
| 43 | *Phb* | gi4505773 | 5.52E-11 | 51.80 | 150.31 |  |  |  |  |  |
|  |  |  | 1.76E-05 |  | 2.74, 2 | 0.46 | 1058.52246 | 0.00762 | K.QVAQQEAER.A |  |
|  |  |  | 6.40E-10 |  | 3.93, 2 | 0.58 | 1189.64233 | 0.00676 | K.KAAIISAEGDSK.A |  |
|  |  |  | 1.51E-06 |  | 3.25, 1 | 0.55 | 1061.54736 | 0.01257 | K.AAIISAEGDSK.A |  |
|  |  |  | 5.38E-08 |  | 2.87, 2 | 0.28 | 1061.54736 | 0.00627 | K.AAIISAEGDSK.A |  |
|  |  |  | 8.24E-05 |  | 2.85, 2 | 0.29 | 867.50470 | 0.00511 | R.PVASQLPR.I |  |
|  |  |  | 1.24E-06 |  | 2.44, 1 | 0.37 | 914.53058 | 0.00562 | R.NVPVITGSK.D |  |
|  |  |  | 8.56E-05 |  | 2.35, 1 | 0.41 | 1023.49933 | 0.01294 | K.EFTEAVEAK.Q |  |
|  |  |  | 3.45E-04 |  | 2.76, 2 | 0.37 | 920.49639 | -0.02686 | K.PIIFDC*R.S | Carbamidomethylation (C) |
|  |  |  | 3.13E-04 |  | 2.89, 1 | 0.24 | 867.50470 | 0.00952 | R.PVASQLPR.I |  |
|  |  |  | 4.49E-08 |  | 3.44, 2 | 0.26 | 1396.84299 | 0.00810 | R.ILFRPVASQLPR.I |  |
|  |  |  | 5.39E-06 |  | 2.29, 1 | 0.36 | 1444.65906 | 0.03210 | R.IFTSIGEDYDER.V |  |
|  |  |  | 1.07E-09 |  | 2.87, 2 | 0.59 | 1444.65906 | 0.01152 | R.IFTSIGEDYDER.V |  |
|  |  |  | 6.03E-04 |  | 1.80, 1 | 0.21 | 1149.58984 | 0.01611 | R.FDAGELITQR.E |  |
|  |  |  | 1.01E-06 |  | 4.73, 3 | 0.46 | 1606.84351 | 0.01187 | R.KLEAAEDIAYQLSR.S |  |
|  |  |  | 5.52E-11 |  | 5.79, 2 | 0.59 | 1606.84351 | 0.01885 | R.KLEAAEDIAYQLSR.S |  |
|  |  |  | 3.18E-07 |  | 3.51, 2 | 0.61 | 1478.74854 | 0.01091 | K.LEAAEDIAYQLSR.S |  |
|  |  |  | 7.80E-11 |  | 5.30, 2 | 0.60 | 1998.08655 | 0.04131 | K.AAELIANSLATAGDGLIELR.K |  |
|  |  |  | 5.70E-09 |  | 3.70, 3 | 0.51 | 1998.08655 | 0.02677 | K.AAELIANSLATAGDGLIELR.K |  |
|  |  |  | 4.91E-10 |  | 4.05, 2 | 0.65 | 1855.03235 | 0.02690 | R.NITYLPAGQSVLLQLPQ |  |
|  |  |  | 8.03E-06 |  | 3.45, 2 | 0.43 | 1185.65857 | 0.01262 | K.DLQNVNITLR.I |  |
|  |  |  | 5.79E-07 |  | 3.75, 2 | 0.35 | 1149.58984 | 0.01055 | R.FDAGELITQR.E |  |
| 44 | *Ppa2* | gi11526789 | 5.51E-13 | 37.13 | 100.24 |  |  |  |  |  |
|  |  |  | 1.65E-05 |  | 2.67, 2 | 0.34 | 1116.56836 | 0.01079 | K.FHDIDDVKK.F |  |
|  |  |  | 4.86E-05 |  | 2.61, 2 | 0.41 | 988.47345 | 0.01109 | K.FHDIDDVK.K |  |
|  |  |  | 4.45E-05 |  | 3.74, 2 | 0.51 | 1233.63208 | 0.01091 | R.SLVESVSSSPNK.E |  |
|  |  |  | 1.05E-06 |  | 3.02, 2 | 0.45 | 1254.71801 | -0.01740 | K.ILSC*GEVIHVK.I | Carbamidomethylation (C) |
|  |  |  | 1.27E-08 |  | 4.02, 2 | 0.58 | 1355.71655 | 0.01689 | K.LIAINANDPEASK.F |  |
|  |  |  | 2.02E-10 |  | 3.93, 3 | 0.47 | 1923.92358 | 0.02469 | K.VPDGKPENQFAFNGEFK.N |  |
|  |  |  | 5.51E-13 |  | 3.92, 2 | 0.63 | 1923.92358 | 0.04070 | K.VPDGKPENQFAFNGEFK.N |  |
|  |  |  | 6.63E-10 |  | 4.74, 3 | 0.58 | 1837.95959 | 0.02579 | K.NVTGHYISPFHDIPLK.V |  |
|  |  |  | 8.28E-09 |  | 3.06, 2 | 0.30 | 2110.00512 | -0.04121 | K.C*NGGAINC*TNVQISDSPFR.C | Carbamidomethylation (C) |
|  |  |  | 7.70E-04 |  | 2.92, 2 | 0.39 | 890.53461 | 0.00749 | K.AFALEVIK.S |  |
|  |  |  | 8.31E-09 |  | 2.76, 2 | 0.51 | 2127.95683 | 0.00537 | K.STNC*FGDNDPIDVC*EIGSK.I | Carbamidomethylation (C) |
| 45 | *Ppp2r1a* | gi119592472 | 1.16E-07 | 10.34 | 60.18 |  |  |  |  |  |
|  |  |  | 2.35E-04 |  | 2.71, 2 | 0.40 | 1239.63281 | 0.01921 | K.ELVSDANQHVK.S |  |
|  |  |  | 2.08E-04 |  | 2.92, 2 | 0.48 | 1160.57935 | 0.01555 | K.LTQDQDVDVK.Y |  |
|  |  |  | 3.30E-07 |  | 3.05, 2 | 0.35 | 1029.51465 | 0.01348 | R.MAGDPVANVR.F |  |
|  |  |  | 5.46E-04 |  | 2.70, 2 | 0.30 | 929.53021 | 0.00896 | K.VLELDNVK.S |  |
|  |  |  | 1.16E-07 |  | 3.58, 2 | 0.48 | 1109.53748 | 0.01567 | R.LAGGDWFTSR.T |  |
|  |  |  | 3.14E-06 |  | 2.77, 2 | 0.53 | 1392.71915 | 0.02467 | K.TDLVPAFQNLM#K.D | Oxidation (M) |
| 46 | *Prdx1* | gi55959887 | 5.83E-07 | 32.70 | 50.19 |  |  |  |  |  |
|  |  |  | 1.05E-05 |  | 2.19, 2 | 0.21 | 980.53125 | 0.00603 | K.IGHPAPNFK.A |  |
|  |  |  | 4.47E-05 |  | 2.41, 2 | 0.46 | 1164.57178 | 0.01579 | K.ATAVMPDGQFK.D |  |
|  |  |  | 1.83E-06 |  | 3.10, 2 | 0.49 | 1107.60449 | 0.01079 | R.TIAQDYGVLK.A |  |
|  |  |  | 5.83E-07 |  | 3.89, 2 | 0.45 | 1196.63098 | 0.01055 | R.LVQAFQFTDK.H |  |
|  |  |  | 2.52E-06 |  | 3.66, 2 | 0.54 | 1638.85195 | 0.02541 | K.QGGLGPM#NIPLVSDPK.R | Oxidation (M) |
| 47 | *Prmt5* | gi88900507 | 5.03E09 | 13.20 | 60.24 |  |  |  |  |  |
|  |  |  | 1.46E-04 |  | 3.58, 2 | 0.31 | 1291.63171 | 0.38054 | K.YSQYQQAIYK.C |  |
|  |  |  | 2.34E-06 |  | 2.69, 2 | 0.40 | 926.54181 | 0.30089 | R.GPLVNASLR.A |  |
|  |  |  | 1.30E-08 |  | 3.54, 3 | 0.50 | 2277.99927 | 1.34665 | R.DDIIENAPTTHTEEYSGEEK.T |  |
|  |  |  | 3.88E-06 |  | 3.11, 2 | 0.51 | 1108.63611 | 0.64079 | R.VPLVAPEDLR.D |  |
|  |  |  | 3.65E-05 |  | 4.80, 3 | 0.35 | 2200.12779 | 0.85777 | R.VPEEEKDTNVQVLM#VLGAGR.G | Oxidation (M) |
|  |  |  | 5.03E-09 |  | 3.56, 2 | 0.53 | 1388.81482 | 0.62444 | K.AAILPTSIFLTNK.K |  |
| 48 | *Psmb6* | gi23110925 | 7.01E-08 | 20.10 | 40.19 |  |  |  |  |  |
|  |  |  | 7.87E-06 |  | 2.88, 2 | 0.39 | 1083.54297 | 0.01116 | R.TTTGSYIANR.V |  |
|  |  |  | 7.01E-08 |  | 2.89, 2 | 0.44 | 1115.60547 | 0.00957 | R.LAAIAESGVER.Q |  |
|  |  |  | 3.35E-05 |  | 2.76 2 | 0.33 | 1110.65173 | 0.01262 | R.QVLLGDQIPK.F |  |
|  |  |  | 3.89E-07 |  | 2.78, 2 | 0.48 | 1982.96154 | 0.01055 | K.EEC*LQFTANALALAM#ER.D | Carbamidomethylation (C), Oxidation (M) |
| 49 | *Rnh1* | gi42822866 | 1.12E-08 | 16.70 | 60.26 |  |  |  |  |  |
|  |  |  | 2.44E-07 |  | 3.47, 2 | 0.60 | 1397.63946 | -0.04481 | K.LESC*GVTSDNC*R.D | Carbamidomethylation (C) |
|  |  |  | 4.71E-06 |  | 3.60, 2 | 0.42 | 1149.55100 | -0.02410 | R.LDDC*GLTEAR.C | Carbamidomethylation (C) |
|  |  |  | 1.12E-08 |  | 4.05, 2 | 0.52 | 1630.80310 | 0.01616 | K.ELTVSNNDINEAGVR.V |  |
|  |  |  | 3.26E-05 |  | 3.26, 2 | 0.42 | 1513.80967 | -0.01068 | R.ELC*QGLGQPGSVLR.V | Carbamidomethylation (C) |
|  |  |  | 1.06E-07 |  | 5.13, 2 | 0.51 | 1530.73950 | 0.02275 | K.ELSLAGNELGDEGAR.L |  |
|  |  |  | 1.29E-06 |  | 2.86, 2 | 0.61 | 1209.69495 | 0.01665 | R.VNPALAELNLR.S |  |
| 50 | *S100a16* | gi17933772 | 7.13E-07 | 22.30 | 20.19 |  |  |  |  |  |
|  |  |  | 7.13E-07 |  | 2.95, 2 | 0.55 | 1365.68701 | 0.02544 | K.LIQNLDANHDGR.I |  |
|  |  |  | 1.46E-06 |  | 3.79, 2 | 0.48 | 1294.74060 | 0.02971 | K.AVIVLVENFYK.Y |  |
| 51 | *Serpinb5* | gi4505789 | 6.66E-15 | 62.70 | 230.33 |  |  |  |  |  |
|  |  |  | 2.61E-07 |  | 4.28, 2 | 0.50 | 1473.79077 | 0.01824 | K.LEETKGQINNSIK.D |  |
|  |  |  | 5.49E-08 |  | 4.80, 2 | 0.44 | 1716.91260 | 0.01689 | K.DKLEETKGQINNSIK.D |  |
|  |  |  | 2.56E-08 |  | 2.60, 1 | 0.44 | 1221.54810 | 0.00671 | K.DVEDESTGLEK.I |  |
|  |  |  | 5.74E-07 |  | 3.32, 2 | 0.48 | 1223.61536 | 0.00090 | K.ELETVDFKDK.L |  |
|  |  |  | 6.82E-07 |  | 2.89, 1 | 0.34 | 1223.61536 | 0.01245 | K.ELETVDFKDK.L |  |
|  |  |  | 2.47E-06 |  | 3.21, 2 | 0.59 | 1221.54810 | 0.00810 | K.DVEDESTGLEK.I |  |
|  |  |  | 2.08E-06 |  | 3.48, 2 | 0.45 | 1591.76978 | 0.01470 | K.DVEDESTGLEKIEK.Q |  |
|  |  |  | 4.80E-09 |  | 2.90, 2 | 0.69 | 1933.81200 | 0.00763 | K.HIFSEDTSDFSGM#SETK.G | Oxidation (M) |
|  |  |  | 2.18E-09 |  | 4.49, 3 | 0.71 | 1933.81200 | 0.00823 | K.HIFSEDTSDFSGM#SETK.G | Oxidation (M) |
|  |  |  | 9.92E-06 |  | 2.36, 1 | 0.39 | 980.49353 | 0.00604 | K.ELETVDFK.D |  |
|  |  |  | 4.65E-08 |  | 4.07, 2 | 0.55 | 1823.92725 | 0.00908 | K.ELETVDFKDKLEETK.G |  |
|  |  |  | 6.66E-15 |  | 2.79, 2 | 0.64 | 1917.81714 | 0.01506 | K.HIFSEDTSDFSGMSETK.G |  |
|  |  |  | 2.09E-09 |  | 5.06, 3 | 0.61 | 1917.81714 | 0.00388 | K.HIFSEDTSDFSGMSETK.G |  |
|  |  |  | 6.48E-08 |  | 5.10, 3 | 0.62 | 2335.25562 | 0.01914 | R.ILQHKDELNADHPFIYIIR.H |  |
|  |  |  | 1.26E-09 |  | 5.86, 2 | 0.57 | 2335.25562 | 0.01885 | R.ILQHKDELNADHPFIYIIR.H |  |
|  |  |  | 2.87E-09 |  | 4.64, 2 | 0.65 | 2223.03462 | 0.02109 | K.QLNSESLSQWTNPSTM#ANAK.V | Oxidation (M) |
|  |  |  | 2.93E-11 |  | 5.01, 4 | 0.60 | 2335.25562 | 0.01284 | R.ILQHKDELNADHPFIYIIR.H |  |
|  |  |  | 6.77E-07 |  | 3.38, 1 | 0.39 | 1426.74243 | 0.01685 | K.SLNLSTEFISSTK.R |  |
|  |  |  | 1.34E-06 |  | 5.22, 3 | 0.62 | 2207.03979 | 0.00913 | K.QLNSESLSQWTNPSTMANAK.V |  |
|  |  |  | 1.10E-12 |  | 3.71, 2 | 0.58 | 2016.98480 | -0.02503 | K.VC*LEITEDGGDSIEVPGAR.I | Carbamidomethylation (C) |
|  |  |  | 2.57E-09 |  | 3.51, 3 | 0.50 | 2016.98480 | -0.01656 | K.VC*LEITEDGGDSIEVPGAR.I | Carbamidomethylation (C) |
|  |  |  | 8.97E-04 |  | 2.92, 1 | 0.31 | 1293.75659 | 0.01917 | K.ILVVNAAYFVGK.W |  |
|  |  |  | 2.57E-07 |  | 5.26, 3 | 0.43 | 2346.08447 | 0.01676 | K.DLTDGHFENILADNSVNDQTK.I |  |
|  |  |  | 1.87E-06 |  | 3.13, 2 | 0.38 | 1198.70166 | 0.00615 | K.HLSMFILLPK.D |  |
|  |  |  | 3.16E-06 |  | 4.34, 2 | 0.53 | 2207.03979 | 0.00981 | K.QLNSESLSQWTNPSTMANAK.V |  |
|  |  |  | 1.37E-09 |  | 4.05, 2 | 0.55 | 1426.74243 | 0.00725 | K.SLNLSTEFISSTK.R |  |
|  |  |  | 9.16E-05 |  | 1.89, 1 | 0.39 | 838.48218 | 0.00610 | R.NIIFFGK.F |  |
|  |  |  | 8.10E-06 |  | 2.91,2 | 0.31 | 1017.57028 | -0.02464 | K.AC*LENLGLK.H | Carbamidomethylation (C) |
|  |  |  | 1.28E-08 |  | 3.84, 2 | 0.64 | 2601.42619 | 0.00037 | K.EPLGNVLFSPIC*LSTSLSLAQVGAK.G | Carbamidomethylation (C) |
|  |  |  | 1.27E-13 |  | 4.53, 2 | 0.52 | 1870.92932 | 0.01482 | K.GDTANEIGQVLHFENVK.D |  |
|  |  |  | 1.23E-11 |  | 3.57, 2 | 0.48 | 1715.87512 | 0.01250 | K.DELNADHPFIYIIR.H |  |
|  |  |  | 1.09E-10 |  | 4.09, 2 | 0.70 | 1293.75659 | 0.00701 | K.ILVVNAAYFVGK.W |  |
|  |  |  | 3.39E-06 |  | 2.51, 2 | 0.11 | 1101.63025 | 0.00444 | K.IIELPFQNK.H |  |
| 52 | *Slc25a24* | gi148491091 | 6.27E-11 | 29.80 | 130.25 |  |  |  |  |  |
|  |  |  | 1.14E-04 |  | 3.20, 2 | 0.50 | 1294.66370 | 0.01506 | K.IFTTGDVNKDGK.L |  |
|  |  |  | 6.34E-06 |  | 3.59, 2 | 0.58 | 1362.62998 | -0.02104 | K.TGQYSGIYDC*AK.K | Carbamidomethylation (C) |
|  |  |  | 3.50E-05 |  | 2.76, 2 | 0.41 | 1287.61502 | 0.01235 | K.SDKM#NIFGGFR.Q | Oxidation (M) |
|  |  |  | 6.81E-09 |  | 3.42, 2 | 0.55 | 1383.71143 | 0.01531 | R.NLGIPLGQDAEEK.I |  |
|  |  |  | 6.27E-11 |  | 4.56, 2 | 0.61 | 1212.70593 | 0.01140 | R.QLLAGGIAGAVSR.T |  |
|  |  |  | 2.34E-04 |  | 2.70, 2 | 0.22 | 957.45108 | 0.00690 | K.M#NIFGGFR.Q | Oxidation (M) |
|  |  |  | 1.86E-10 |  | 5.02, 3 | 0.46 | 2428.12327 | 0.02550 | R.FISGSM#AGATAQTFIYPM#EVM#K.T | Oxidation (M) |
|  |  |  | 1.68E-09 |  | 4.17, 2 | 0.55 | 1670.83435 | 0.02507 | R.NGDGVVDIGELQEGLR.N |  |
|  |  |  | 5.89E-05 |  | 2.72, 2 | 0.46 | 1143.54688 | 0.01213 | K.SYWLDNFAK.D |  |
|  |  |  | 1.12E-11 |  | 3.40, 2 | 0.52 | 1797.94552 | 0.02755 | K.VLPAVGISYVVYENM#K.Q | Oxidation (M) |
|  |  |  | 3.39E08 |  | 3.13, 2 | 0.42 | 1255.63171 | 0.01189 | R.YETLFQALDR.N |  |
|  |  |  | 3.27E-10 |  | 2.88, 2 | 0.59 | 1781.95056 | 0.02397 | K.VLPAVGISYVVYENMK.Q |  |
| 53 | *Tkt* | gi14250367 | 2.11E-14 | 29.40 | 90.31 |  |  |  |  |  |
|  |  |  | 7.88E-04 |  | 2.88, 2 | 0.26 | 949.54657 | 0.70171 | R.KAYGQALAK.L |  |
|  |  |  | 6.41E-05 |  | 2.70, 2 | 0.43 | 916.50983 | 0.11699 | K.AVELAANTK.G |  |
|  |  |  | 1.58E-04 |  | 2.73, 2 | 0.22 | 915.56219 | 0.45964 | R.KLILDSAR.A |  |
|  |  |  | 5.93E-05 |  | 2.82, 2 | 0.30 | 1200.62585 | 0.45268 | K.NSTFSEIFKK.E |  |
|  |  |  | 2.11E-14 |  | 6.15, 3 | 0.37 | 2508.21143 | 0.71708 | R.TSRPENAIIYNNNEDFQVGQAK.V |  |
|  |  |  | 2.38E-11 |  | 3.19, 2 | 0.60 | 1884.92261 | -0.29170 | R.SVPTSTVFYPSDGVATEK.A |  |
|  |  |  | 2.11E-14 |  | 4.28, 2 | 0.60 | 2020.07092 | 0.34966 | K.ILATPPQEDAPSVDIANIR.M |  |
|  |  |  | 1.08E-05 |  | 2.90, 2 | 0.45 | 1413.81006 | -0.12007 | R.VLDPFTIKPLDR.K |  |
|  |  |  | 4.79E-12 |  | 4.65, 3 | 0.59 | 2020.07092 | 0.11649 | K.ILATPPQEDAPSVDIANIR.M |  |
| 54 | *Tom1* | O60784 | 2.78E-14 |  | 130.38 |  |  |  |  |  |
|  |  |  | 3.36E-06 |  | 2.76, 2 | 0.37 | 1134.59021 | 0.00823 | K.NNPPTIVHDK.V |  |
|  |  |  | 3.00E-05 |  | 3.59, 2 | 0.47 | 1236.58886 | 0.01372 | R.SELEM#VSGNVR.V | Oxidation (M) |
|  |  |  | 1.13E-06 |  | 4.13, 2 | 0.32 | 1088.56946 | 0.00896 | R.AGLQSLEASGR.L |  |
|  |  |  | 1.24E-11 |  | 7.70, 3 | 0.58 | 2495.26367 | 0.03135 | K.AADRLPNLSSPSAEGPPGPPSGPAPR.K |  |
|  |  |  | 2.78E-14 |  | 4.92, 2 | 0.67 | 2082.06152 | 0.04375 | R.LPNLSSPSAEGPPGPPSGPAPR.K |  |
|  |  |  | 1.71E-10 |  | 4.73, 3 | 0.39 | 1718.83435 | 0.01712 | K.YEAPQATDGLAGALDAR.Q |  |
|  |  |  | 9.54E-12 |  | 4.58, 2 | 0.63 | 1718.83435 | 0.02678 | K.YEAPQATDGLAGALDAR.Q |  |
|  |  |  | 4.80E-10 |  | 4.04, 2 | 0.52 | 1685.80176 | 0.02214 | K.GVTSEEFDKFLEER.A |  |
|  |  |  | 9.52E-07 |  | 2.74, 2 | 0.40 | 1441.65152 | 0.02064 | K.TQEKDDDM#LFAL | Oxidation (M) |
|  |  |  | 5.45E-05 |  | 2.76, 2 | 0.48 | 1502.68316 | 0.01501 | R.LEDEFDM#FALTR.G | Oxidation (M) |
|  |  |  | 5.03E-07 |  | 3.63, 3 | 0.54 | 3829.78546 | 0.10856 | K.APSEAEPAADLIDM#GPDPAATGNLSSQLAGM#NLGSSSVR.A | Oxidation (M) |
|  |  |  | 7.03E-05 |  | 2.95, 2 | 0.45 | 1486.68823 | 0.01201 | R.LEDEFDMFALTR.G |  |
|  |  |  | 4.12E-10 |  | 3.98, 2 | 0.64 | 1764.90137 | 0.02434 | R.SSPDLTGVVTIYEDLR.R |  |
| 55 | *Trap1/Hsp90l* | gi155722983 | 1.89E-14 | 33.40 | 260.31 |  |  |  |  |  |
|  |  |  | 9.86E-07 |  | 3.57, 2 | 0.61 | 1184.61707 | -0.52180 | R.YVAQAHDKPR.Y |  |
|  |  |  | 6.21E-05 |  | 3.16, 2 | 0.42 | 989.46869 | 0.09270 | K.HEFQAETK.K |  |
|  |  |  | 7.48E-10 |  | 4.15, 2 | 0.43 | 1575.72607 | 1.43804 | R.HLAEHSPYYEAMK.K |  |
|  |  |  | 3.10E-05 |  | 3.58, 2 | 0.54 | 1303.67395 | 0.32567 | R.EGIVTATEQEVK.E |  |
|  |  |  | 2.30E-04 |  | 1.80, 1 | 0.37 | 1303.67395 | -0.19922 | R.EGIVTATEQEVK.E |  |
|  |  |  | 1.75E-05 |  | 3.54, 2 | 0.47 | 1843.90405 | 0.35039 | R.LDTHPAM#VTVLEM#GAAR.H | Oxidation (M) |
|  |  |  | 2.32E-07 |  | 5.18, 3 | 0.42 | 1843.90405 | -0.51266 | R.LDTHPAM#VTVLEM#GAAR.H | Oxidation (M) |
|  |  |  | 6.78E-05 |  | 3.11, 2 | 0.48 | 1327.56567 | 1.45489 | K.ETEELM#AWM#R.N | Oxidation (M) |
|  |  |  | 3.04E-06 |  | 3.20, 2 | 0.41 | 1181.61609 | 0.06194 | R.ELGSSVALYSR.K |  |
|  |  |  | 1.72E-09 |  | 3.53, 3 | 0.36 | 2339.15474 | 1.48830 | K.LVSDGQALPEM#EIHLQTNAEK.G | Oxidation (M) |
|  |  |  | 2.71E-06 |  | 3.64, 2 | 0.51 | 1289.65833 | 0.30486 | R.ELISNASDALEK.L |  |
|  |  |  | 9.31E-10 |  | 3.28, 2 | 0.43 | 2339.15474 | -0.06045 | K.LVSDGQALPEM#EIHLQTNAEK.G | Oxidation (M) |
|  |  |  | 7.19E-07 |  | 4.50, 3 | 0.38 | 1827.90915 | 1.25311 | R.LDTHPAM#VTVLEMGAAR.H | Oxidation (M) |
|  |  |  | 2.60E-05 |  | 3.44, 2 | 0.36 | 1827.90915 | 0.58284 | R.LDTHPAM#VTVLEMGAAR.H | Oxidation (M) |
|  |  |  | 5.13E-07 |  | 4.10, 2 | 0.36 | 1827.90915 | 0.38972 | R.LDTHPAMVTVLEM#GAAR.H | Oxidation (M) |
|  |  |  | 4.30E-05 |  | 5.35, 3 | 0.42 | 1827.90915 | 1.49829 | R.LDTHPAMVTVLEM#GAAR.H | Oxidation (M) |
|  |  |  | 5.54E-05 |  | 2.76, 2 | 0.43 | 1311.75077 | -0.27751 | K.ETEELMAWM#R.N | Oxidation (M) |
|  |  |  | 2.61E-08 |  | 3.66, 3 | 0.27 | 2323.15991 | 0.77750 | K.LVSDGQALPEMEIHLQTNAEK.G |  |
|  |  |  | 2.26E-05 |  | 3.48, 3 | 0.45 | 1492.84814 | 0.58182 | R.AQLLQPTLEINPR.H |  |
|  |  |  | 1.19E-06 |  | 3.94, 2 | 0.44 | 1492.84814 | 0.38561 | R.AQLLQPTLEINPR.H |  |
|  |  |  | 2.04E-05 |  | 4.97, 2 | 0.56 | 1626.73240 | 0.25226 | R.M#NTLQAIWM#M#DPK.D | Oxidation (M) |
|  |  |  | 1.14E-09 |  | 5.10, 2 | 0.58 | 1592.79150 | -0.08345 | K.AFLDALQNQAEASSK.I |  |
|  |  |  | 1.58E-06 |  | 4.74, 2 | 0.46 | 1527.80127 | 0.74907 | R.GVVDSEEIPLNLSR.E |  |
|  |  |  | 3.52E-08 |  | 4.92, 3 | 0.43 | 1811.91431 | 1.36814 | R.LDTHPAMVTVLEMGAAR.H |  |
|  |  |  | 2.76E-05 |  | 3.52, 3 | 0.43 | 1592.79150 | 0.92057 | K.AFLDALQNQAEASSK.I |  |
|  |  |  | 3.04E-08 |  | 4.35, 2 | 0.53 | 1811.91431 | 1.41216 | R.LDTHPAMVTVLEMGAAR.H |  |
|  |  |  | 1.89E-14 |  | 6.17, 2 | 0.68 | 2146.02979 | 1.20220 | R.YESSALPSGQLTSLSEYASR.M |  |
|  |  |  | 2.77E-10 |  | 4.18, 3 | 0.62 | 2146.02979 | -0.14944 | R.YESSALPSGQLTSLSEYASR.M |  |
|  |  |  | 1.69E-07 |  | 3.26, 2 | 0.52 | 1295.57593 | 0.72740 | K.ETEELMAWMR.N |  |
|  |  |  | 3.13E-06 |  | 3.06, 2 | 0.45 | 1340.59797 | 0.46946 | K.FFEDYGLFM#R.E | Oxidation (M) |
|  |  |  | 8.03E-07 |  | 3.31, 2 | 0.60 | 1324.60303 | 0.33086 | K.FFEDYGLFMR.E |  |
|  |  |  | 1.41E-04 |  | 2.89, 2 | 0.24 | 1171.66809 | 0.19890 | R.ELLQESALIR.K |  |
| 56 | *Tubb4* | gi21361322 | 9.14E-15 |  | 60.21 |  |  |  |  |  |
|  |  |  | 4.42E-07 |  | 4.00, 2 | 0.55 | 1335.69768 | 0.01232 | R.IM#NTFSVVPSPK.V |  |
|  |  |  | 3.00E-06 |  | 3.51, 2 | 0.52 | 1245.59322 | 0.00900 | R.ISEQFTAM#FR.R |  |
|  |  |  | 1.34E-04 |  | 3.80, 2 | 0.47 | 1319.70276 | 0.01360 | R.IMNTFSVVPSPK.V |  |
|  |  |  | 9.14E-12 |  | 3.89, 2 | 0.57 | 1631.83088 | 0.01816 | R.AILVDLEPGTM#DSVR.S |  |
|  |  |  | 6.51E-09 |  | 3.09, 2 | 0.24 | 1707.86218 | 0.02043 | R.ALTVPELTQQM#FDAK.N |  |
|  |  |  | 6.05E-10 |  | 3.72, 2 | 0.56 | 1615.83594 | 0.02239 | R.AILVDLEPGTMDSVR.S |  |
| 57 | *Tunp* | P61978 | 1.33E-13 | 22.60 | 80.25 |  |  |  |  |  |
|  |  |  | 1.45E-06 |  | 3.11, 2 | 0.49 | 1549.71330 | -0.04601 | K.LFQEC*C*PHSTDR.V | Carbamidomethylation (C) |
|  |  |  | 2.31E-06 |  | 2.94, 2 | 0.37 | 1053.64148 | 0.00640 | R.VVLIGGKPDR.V |  |
|  |  |  | 8.50E-08 |  | 3.77, 2 | 0.52 | 1259.57495 | 0.01042 | K.IDEPLEGSEDR.I |  |
|  |  |  | 1.33E-13 |  | 2.96, 2 | 0.55 | 1780.79846 | 0.02251 | R.TDYNASVSVPDSSGPER.I |  |
|  |  |  | 1.19E-04 |  | 3.11, 2 | 0.35 | 1106.51465 | 0.00713 | R.NTDEMVELR.I |  |
|  |  |  | 7.40E-10 |  | 4.22, 2 | 0.64 | 1518.93665 | 0.01702 | R.LLIHQSLAGGIIGVK.G |  |
|  |  |  | 3.65E-11 |  | 4.76, 2 | 0.56 | 1917.03271 | 0.02471 | R.GSYGDLGGPIITTQVTIPK.D |  |
|  |  |  | 1.61E-07 |  | 3.90, 2 | 0.54 | 1340.80359 | 0.01592 | K.IILDLISESPIK.G |  |
| 58 | *Vil2/Ezr* | P15311 | 8.51E-11 |  | 108.25 |  |  |  |  |  |
|  |  |  | 1.20E-04 |  | 3.69, 3 | 0.33 | 1488.78387 | 0.25037 | R.RKPDTIEVQQM#K.A | Oxidation (M) |
|  |  |  | 1.28E-04 |  | 3.35, 2 | 0.38 | 1116.58948 | 1.18572 | R.AKEAQDDLVK.T |  |
|  |  |  | 1.13E-04 |  | 4.10, 3 | 0.37 | 1640.77759 | 0.18320 | R.RKEDEVEEWQHR.A |  |
|  |  |  | 9.85E-04 |  | 3.52, 3 | 0.13 | 1472.78894 | 0.98447 | R.RKPDTIEVQQMK.A |  |
|  |  |  | 1.45E-04 |  | 2.75, 2 | 0.38 | 898.42651 | 0.16893 | K.SGYLSSER.L |  |
|  |  |  | 3.64E-04 |  | 2.77, 2 | 0.31 | 914.53058 | 0.99657 | K.IALLEEAR.R |  |
|  |  |  | 5.75E-08 |  | 4.31, 2 | 0.47 | 2082.00595 | 0.92354 | R.VTTM#DAELEFAIQPNTTGK.Q | Oxidation (M) |
|  |  |  | 3.05E-07 |  | 3.92, 2 | 0.50 | 1445.79578 | 0.13890 | R.QLLTLSSELSQAR.D |  |
|  |  |  | 8.51E-11 |  | 5.04, 2 | 0.63 | 1651.81738 | 0.42095 | K.SQEQLAAELAEYTAK.I |  |
|  |  |  | 3.23E-06 |  | 3.89, 2 | 0.56 | 1182.59424 | 0.68047 | K.APDFVFYAPR.L |  |
|  |  |  | 1.29E-06 |  | 4.89, 3 | 0.52 | 1651.81738 | 0.87589 | K.SQEQLAAELAEYTAK.I |  |
|  |  |  | 8.24E-06 |  | 3.13, 2 | 0.37 | 1104.58362 | 0.49480 | K.IGFPWSEIR.N |  |
| 59 | *Vdac2* | gi119574957 | 2.41E-12 | 47.44 | 110.29 |  |  |  |  |  |
|  |  |  | 5.11E-12 |  | 3.28, 2 | 0.71 | 1907.82288 | 0.00505 | K.SC*SGVEFSTSGSSNTDTGK.V | Carbamidomethylation (C) |
|  |  |  | 1.53E-05 |  | 3.51, 3 | 0.58 | 1907.82288 | -0.00619 | K.SC*SGVEFSTSGSSNTDTGK.V | Carbamidomethylation (C) |
|  |  |  | 5.12E-05 |  | 2.91, 2 | 0.42 | 940.46356 | 0.00725 | R.NNFAVGYR.T |  |
|  |  |  | 3.82E-06 |  | 2.94, 2 | 0.43 | 1464.71667 | -0.00544 | R.PM#C*IPPSYADLGK.A | Oxidation (M), Carbamidomethylation (C) |
|  |  |  | 4.39E-07 |  | 3.28, 2 | 0.47 | 1293.66846 | 0.01811 | K.YQLDPTASISAK.V |  |
|  |  |  | 2.41E-12 |  | 5.00, 2 | 0.41 | 2103.15576 | 0.04521 | K.VNNSSLIGVGYTQTLRPGVK.L |  |
|  |  |  | 2.62E-12 |  | 5.88, 3 | 0.66 | 2528.16870 | 0.04850 | R.TGDFQLHTNVNDGTEFGGSIYQK.V |  |
|  |  |  | 3.68E-06 |  | 2.99, 2 | 0.42 | 1448.72177 | 0.00411 | R.PMC*IPPSYADLGK.A | Carbamidomethylation (C) |
|  |  |  | 1.70E-10 |  | 4.72, 2 | 0.49 | 1721.91809 | 0.03081 | K.VNNSSLIGVGYTQTLR.P |  |
|  |  |  | 2.43E-06 |  | 3.74, 2 | 0.59 | 1428.70056 | 0.01885 | K.LTFDTTFSPNTGK.K |  |
|  |  |  | 2.01E-06 |  | 3.22, 2 | 0.46 | 1016.59863 | 0.00890 | K.LTLSALVDGK.S |  |
|  |  |  | 1.01E-04 |  | 3.58, 3 | 0.41 | 2399.12127 | 0.00272 | K.VC*EDLDTSVNLAWTSGTNC*TR.F | Carbamidomethylation (C) |
|  |  |  | 6.11E-07 |  | 3.64, 2 | 0.69 | 2399.12127 | 0.02526 | K.VC*EDLDTSVNLAWTSGTNC*TR.F | Carbamidomethylation (C) |
| 60 | *Vim* | P08670 | 4.44E-15 | 62.40 | 388.29 |  |  |  |  |  |
|  |  |  | 3.59E-06 |  | 3.70, 2 | 0.39 | 1216.62805 | 0.00542 | R.RQVDQLTNDK.A |  |
|  |  |  | 4.15E-05 |  | 3.21, 2 | 0.24 | 1060.56335 | 0.00432 | R.KLLEGEESR.I |  |
|  |  |  | 7.32E-04 |  | 1.75, 1 | 0.35 | 1060.52698 | 0.01184 | R.QVDQLTNDK.A |  |
|  |  |  | 9.54E-05 |  | 2.70, 2 | 0.48 | 1766.80473 | 0.01587 | R.LQDEIQNM#KEEM#AR.H | Oxidation (M) |
|  |  |  | 5.62E-06 |  | 2.77, 2 | 0.30 | 1134.54594 | 0.00746 | R.LQDEIQNM#K.E | Oxidation (M) |
|  |  |  | 2.26E-04 |  | 2.75, 2 | 0.35 | 932.46832 | 0.00340 | K.LLEGEESR.I |  |
|  |  |  | 1.14E-05 |  | 3.38, 2 | 0.29 | 1319.66236 | 0.00858 | R.EKLQEEM#LQR.E | Oxidation (M) |
|  |  |  | 9.38E-07 |  | 2.99, 2 | 0.36 | 1088.53308 | 0.00554 | R.QDVDNASLAR.L |  |
|  |  |  | 5.74E-04 |  | 3.07, 2 | 0.28 | 1062.52481 | 0.00600 | K.LQEEM#LQR.E | Oxidation (M) |
|  |  |  | 5.48E-07 |  | 3.57, 2 | 0.50 | 1750.80983 | 0.01358 | R.LQDEIQNM#KEEMAR.H | Oxidation (M) |
|  |  |  | 4.44E-15 |  | 5.78, 3 | 0.60 | 2423.10693 | 0.01688 | K.TVETRDGQVINETSQHHDDLE |  |
|  |  |  | 2.72E-10 |  | 2.74, 2 | 0.53 | 1836.79944 | 0.01311 | R.DGQVINETSQHHDDLE |  |
|  |  |  | 4.65E-07 |  | 3.21, 2 | 0.26 | 1750.80983 | 0.02506 | R.LQDEIQNMKEEM#AR.H | Oxidation (M) |
|  |  |  | 9.67E-06 |  | 2.95, 2 | 0.35 | 1303.66748 | 0.00762 | R.EKLQEEMLQR.E |  |
|  |  |  | 1.15E-04 |  | 3.08, 2 | 0.34 | 1118.55103 | 0.00530 | R.LQDEIQNMK.E |  |
|  |  |  | 5.61E-04 |  | 2.60, 1 | 0.19 | 870.43561 | 0.00415 | R.FANYIDK.V |  |
|  |  |  | 1.41E-06 |  | 3.24, 2 | 0.36 | 1125.60510 | 0.00102 | R.FANYIDKVR.F |  |
|  |  |  | 2.93E-06 |  | 2.21, 1 | 0.33 | 1093.52722 | 0.00623 | K.FADLSEAANR.N |  |
|  |  |  | 5.15E-07 |  | 3.03, 2 | 0.41 | 1270.56198 | 0.00643 | R.LGDLYEEEM#R.E | Oxidation (M) |
|  |  |  | 8.38E-07 |  | 3.75, 2 | 0.37 | 1093.52722 | 0.00469 | K.FADLSEAANR.N |  |
|  |  |  | 9.20E-11 |  | 4.08, 3 | 0.47 | 1704.82211 | 0.00532 | R.VEVERDNLAEDIM#R.L | Oxidation (M) |
|  |  |  | 2.64E-08 |  | 3.24, 2 | 0.50 | 1824.94507 | 0.01482 | R.ETNLDSLPLVDTHSKR.T |  |
|  |  |  | 1.07E-06 |  | 4.03, 2 | 0.42 | 1346.88357 | -0.01743 | R.RQVQSLTC*EVDALK.G | Carbamidomethylation (C) |
|  |  |  | 2.27E-07 |  | 3.84, 2 | 0.40 | 1734.81494 | 0.00957 | R.LQDEIQNMKEEMAR.H |  |
|  |  |  | 3.76E-06 |  | 3.23, 2 | 0.15 | 1439.75626 | 0.00380 | K.M#ALDIEIATYRK.L | Oxidation (M) |
|  |  |  | 4.43E-05 |  | 2.55, 1 | 0.41 | 1254.56714 | 0.01062 | R.LGDLYEEEMR.E |  |
|  |  |  | 9.59E-08 |  | 3.01, 2 | 0.44 | 1497.70020 | 0.00774 | K.SRLGDLYEEEMR.E |  |
|  |  |  | 3.13E-07 |  | 3.51, 2 | 0.43 | 1668.84387 | 0.00408 | R.ETNLDSLPLVDTHSK.R |  |
|  |  |  | 1.12E-04 |  | 2.39, 1 | 0.27 | 1121.58374 | 0.01184 | R.EYQDLLNVK.M |  |
|  |  |  | 9.84E-05 |  | 2.23, 1 | 0.21 | 1309.60596 | 0.01501 | K.NLQEAEEWYK.S |  |
|  |  |  | 2.59E-08 |  | 4.16, 3 | 0.42 | 1688.82727 | 0.00589 | R.VEVERDNLAEDIMR.L |  |
|  |  |  | 7.97E-06 |  | 3.07, 2 | 0.37 | 1688.82727 | 0.00847 | R.VEVERDNLAEDIMR.L |  |
|  |  |  | 5.76E-07 |  | 3.00, 2 | 0.47 | 1309.60596 | 0.00408 | K.NLQEAEEWYK.S |  |
|  |  |  | 1.49E-08 |  | 5.58, 3 | 0.46 | 1661.94727 | 0.00895 | R.KVESLQEEIAFLKK.L |  |
|  |  |  | 2.26E-09 |  | 5.39, 2 | 0.50 | 1661.94727 | 0.00774 | R.KVESLQEEIAFLKK.L |  |
|  |  |  | 1.34E-05 |  | 3.46, 2 | 0.46 | 1490.78246 | -0.02106 | R.QVQSLTC*EVDALK.G | Carbamidomethylation (C) |
|  |  |  | 1.26E-10 |  | 4.79, 2 | 0.49 | 1539.91052 | 0.00664 | K.ILLAELEQLKGQGK.S |  |
|  |  |  | 5.97E-05 |  | 2.59, 1 | 0.47 | 1076.50403 | 0.00964 | R.DNLAEDIMR.L |  |
|  |  |  | 7.18E-04 |  | 2.75, 2 | 0.34 | 1076.50403 | 0.00408 | R.DNLAEDIMR.L |  |
|  |  |  | 8.56E-08 |  | 5.58, 2 | 0.47 | 1533.85229 | 0.00652 | R.KVESLQEEIAFLK.K |  |
|  |  |  | 2.26E-07 |  | 5.39, 3 | 0.45 | 1533.85229 | 0.00516 | R.KVESLQEEIAFLK.K |  |
|  |  |  | 1.50E-07 |  | 3.99, 2 | 0.18 | 1295.66638 | 0.00823 | K.MALDIEIATYR.K |  |
|  |  |  | 4.41E-08 |  | 3.69, 2 | 0.44 | 1405.75732 | 0.00762 | K.VESLQEEIAFLK.K |  |
|  |  |  | 8.71E-06 |  | 3.62, 2 | 0.23 | 1169.71399 | 0.00640 | K.ILLAELEQLK.G |  |
|  |  |  | 1.45E-06 |  | 2.96, 2 | 0.46 | 1254.56714 | 0.00725 | R.LGDLYEEEMR.E |  |
|  |  |  | 7.13E-10 |  | 2.80, 2 | 0.36 | 1570.89514 | 0.01116 | R.ISLPLPNFSSLNLR.E |  |
| 61 | *Xrcc5* | gi10863945 | 2.17E-07 |  | 90.21 |  |  |  |  |  |
|  |  |  | 6.40E-07 |  | 3.04, 2 | 0.45 | 1522.72314 | 0.56377 | K.EEASGSSVTAEEAKK.F |  |
|  |  |  | 2.17E-07 |  | 4.18, 2 | 0.51 | 1394.62817 | 0.61821 | K.EEASGSSVTAEEAK.K |  |
|  |  |  | 8.46E-04 |  | 2.78, 2 | 0.41 | 1109.61023 | -0.36336 | R.LGGHGPSFPLK.G |  |
|  |  |  | 1.00E-04 |  | 2.73, 2 | 0.45 | 1099.56046 | 0.27528 | R.FFM#GNQVLK.V | Oxidation (M) |
|  |  |  | 2.39E05 |  | 3.40, 3 | 0.44 | 2243.02100 | 0.46811 | K.DQVTAQEIFQDNHEDGPTAK.K |  |
|  |  |  | 2.14E-04 |  | 3.12, 2 | 0.40 | 993.55499 | 0.43993 | K.VITMFVQR.Q |  |
|  |  |  | 1.16E-05 |  | 3.68, 2 | 0.58 | 1112.56226 | 0.48039 | R.YGSDIVPFSK.V |  |
|  |  |  | 2.51E-04 |  | 2.51, 2 | 0.33 | 977.50507 | 0.58849 | K.EDIIQGFR.Y |  |
|  |  |  | 9.65E-04 |  | 3.56, 2 | 0.49 | 1380.68933 | 0.63542 | K.TDTLEDLFPTTK.I |  |
|  |  |  |  |  |  |  |  |  |  |  |

P (pro): The protein probability is the highest peptide probability that was found

P (pep): The peptide probability that the search could have produced a better match using a random sequence.

Coverage: The percentage of identified peptides

Score: This is the original Eng-Yates-Scoring, in which a primary match receives 10 points, a secondary match receives 8 points, a tertiary match gets 6 points, 4 points, 2 points, etc. The points for all the peptides are summed together. This was the original way of sorting a consensus report.

Xc: The cross correlation of the primary match. Xc (Cross Correlation) is a measure of the “goodness of fit” of a theoretical spectra created from the sequences b + y ions, along with water and amine losses compared to the actual MS/MS spectra acquired by the instrument.

ΔCn: The delta correlation of the primary match being compared to the secondary match.

ΔM: The delta mass is the difference between the mass theoretical mass of the peptide identified and the actual mass measurement of the instrument.
